# All Visible Light Photoswitch Based on the Dimethyldihydropyrene Unit Operating in Aqueous Solutions with High Quantum Yields

**DOI:** 10.1021/jacsau.2c00552

**Published:** 2022-12-21

**Authors:** Zakaria Ziani, Saioa Cobo, Frédérique Loiseau, Damien Jouvenot, Elise Lognon, Martial Boggio-Pasqua, Guy Royal

**Affiliations:** † Univ. Grenoble Alpes, CNRS, DCM, Grenoble38000, France; ‡ LCPQ UMR 5626, CNRS et Université Toulouse III − Paul Sabatier, 118 route de Narbonne, Toulouse31062, France

**Keywords:** photochromism, dimethyldihydropyrene, spin-flip TD-DFT, spectroscopy, electrochemistry

## Abstract

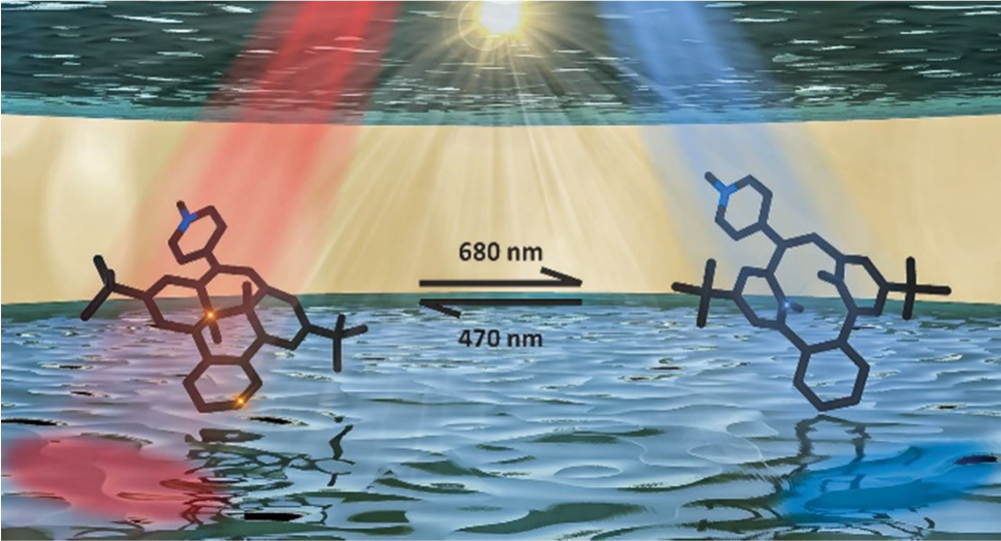

Molecular systems and devices whose properties can be modulated using light as an external stimulus are the subject of numerous research studies in the fields of materials and life sciences. In this context, the use of photochromic compounds that reversibly switch upon light irradiation is particularly attractive. However, for many envisioned applications, and in particular for biological purposes, illumination with harmful UV light must be avoided and these photoactivable systems must operate in aqueous media. In this context, we have designed a benzo[*e*]-fused dimethyldihydropyrene compound bearing a methyl-pyridinium electroacceptor group that meets these requirements. This compound (closed state) is able to reversibly isomerize under aerobic conditions into its corresponding cyclophanediene form (open isomer) through the opening of its central carbon–carbon bond. Both the photo-opening and the reverse photoclosing processes are triggered by visible light illumination and proceed with high quantum yields (respectively 14.5% yield at λ = 680 nm and quantitative quantum yield at λ = 470 nm, in water). This system has been investigated by nuclear magnetic resonance and absorption spectroscopy, and the efficient photoswitching behavior was rationalized by spin-flip time-dependent density functional theory calculations. In addition, it is demonstrated that the isomerization from the open to the closed form can be electrocatalytically triggered.

## Introduction

Nowadays, thanks to the wide availability of different light sources (LEDs, lasers, etc.) as well as optical fibers and devices, the ability to control the properties of molecules and materials using light is the subject of numerous research studies in the fields of smart materials, (photo)catalysis, energy, molecular electronics, or life sciences.^1,2^ In this context, an attractive strategy is to use photoswitches (photochromic compounds) that can act as key components for such applications.^3^ These compounds can be interconverted between two (or more) isomeric forms upon absorption of electromagnetic radiations, and their isomers usually display distinct properties such as polarizability, solubility, luminescence, shape, or conductivity. It is thus possible to optically modulate their properties, activities, and interactions with their environment. For these reasons, numerous applications involving photoswitches are now envisioned in the fields of photopharmacology and biology by combining noninvasive inputs (light) with biocompatible photoswitchable molecules or molecular materials.^4−7^ For instance, fascinating photoswitches have been reported for drug delivery,^8−10^ oncology,^11,12^ regenerative medicine^13−15^ or super-resolution imaging.^16−18^

The efficiency of a photochromic compound is crucial for aforementioned applications and can be mainly described by four parameters: (i) the photoisomerization quantum yield (Φ) that quantifies the number of photoisomerized molecules per number of absorbed photons; (ii) the photostationary state (PSS), which describes the equilibrium between the different isomers under light irradiation; (iii) the thermal stability of the isomers; and (iv) the fatigue resistance. For most applications, an efficient photoswitch usually requires an almost complete interconversion between the two isomers, associated with high quantum yields and a good fatigue resistance. These conditions have been satisfied with several photochromic families such as azobenzenes, spiropyrans, or dithienylethenes, opening the door to a great variety of applications as functional materials,^19−21^ from optoelectronics^22−24^ to information storage purposes.^25,26^ However, their use in life sciences still remains a challenge due to the inherent poor water solubility of organic photochromic derivatives preventing their application for in vitro and in vivo conditions. In addition, the photoisomerization process is often induced by harmful UV light, and the use of photoswitches that can be reversibly activated by nondamaging visible light is thus required.^27−33^ As a result, the development of robust and water-compatible photoswitchable compounds for biological applications stands as a highly desirable and challenging task.

Several strategies can be used to solubilize organic photochromic molecules in aqueous media. For example, photoswitches relying on double-bond photoisomerization (azobenzenes or stilbenes) have been described as water-soluble, and their interactions with solvent molecules as well as their solubilities were modulated due to the polarity difference between the two isomers (*Z*/*E*).^34,35^ However, the main strategies that are currently used for the solubilization of organic photoswitches in water are based on their encapsulation in water-soluble hosts or the introduction of solubilizing groups onto the photochromic core, such as hydrophilic chains and/or ionic units. Among the solubilizing ionic groups, carboxylate, phosphonates, sulfonates, and ammoniums are usually employed.^34,35^ The hydrophilicity of several dithienylethene compounds was also improved upon incorporation of pyridinium groups.^36−38^

**Scheme 1:**
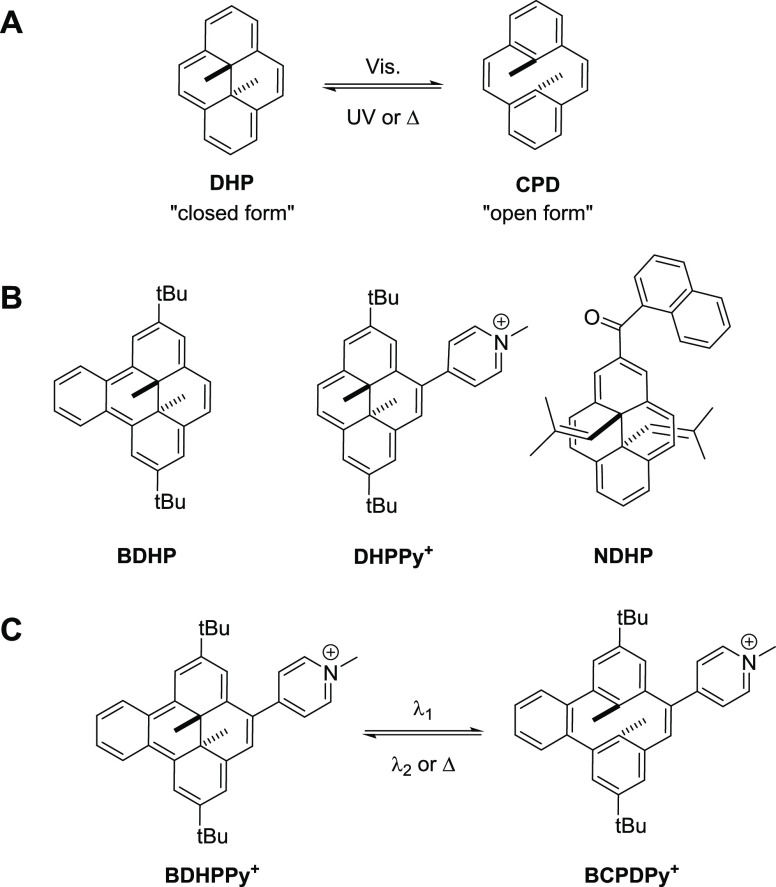
(A) Dimethyldihydropyrene (**DHP**)/Cyclophanediene (**CPD**) Isomerization; (B) Structures of **BDHP**, **DHPPy^+^**, and **NDHP**; (C) Representation of the Investigated **BDHPPy^+^**/**BCPDPy^+^** Couple

In this context, the goal of this work was to design a photoswitch that can be reversibly converted (i) with high quantum yields in both directions, (ii) by illumination with visible light, and (iii) in aqueous media. To reach such objectives, the *trans*-dimethyldihydropyrene/cyclophanediene photochromic couple (**DHP**/**CPD**, Scheme 1A) appeared as a promising candidate. The green dimethyldihydropyrene unit (**DHP**; *trans*-10*b*,10*c*-dimethyl-10*b*,10*c*-dihydropyrene; closed form) contains a rigid extended 14 π-electron system with two methyl groups pointing in opposite directions on either side of the aromatic plane and can be converted with a high PSS to its corresponding colorless cyclophanediene isomer (**CPD**; open form) upon illumination with visible light, through the opening of the central carbon–carbon bond.

The reverse process (**CPD** → **DHP**; open to closed form) is usually obtained by UV light irradiation or thermally. This system, which is a rare example of negative photochromic couple (i.e., the colored state is the thermodynamically more stable form), has been much less investigated than other well-known photoswitches, mainly because of its low quantum yield of photo-opening (0.6% when illuminated at 380 nm in cyclohexane).^39^ However, the DHP skeleton constitutes a true molecular platform that may be chemically functionalized in many different ways^40^ in order to finely tune and improve its (photochromic) properties, and in particular to address these systems in the visible region while increasing their quantum yields of photoisomerization. In this context, among different possible approaches, a successful strategy to shift the wavelengths needed for the photoactivation of dimethyldihydropyrene systems toward lower energies is to incorporate opposite donor–acceptor substituents onto the DHP core.^41−43^ For example, switchable donor–acceptor DHPs working with near-infrared light were recently designed by Hecht,^44,45^ although the reverse reaction (from CPD to DHP forms) was done thermally due to the moderate thermal lifetime of the open states.

Subtle chemical functionalization of the DHP moiety can also dramatically increase its quantum yields of isomerization. In particular, Mitchell and co-workers have demonstrated that the photoconversion rate of DHPs can be significantly improved using annelated benzenoid-DHP derivatives and in particular the benzo[*e*]-DHP compound (**BDHP**, Scheme 1B). Indeed, this compound can be isomerized with a photo-ring-opening quantum yield of Φ_c–o_ = 7.4% upon illumination at λ_ex_ = 389 nm in toluene.^46^ In 2011, the naphthoyl-substituted DHP compound (**NDHP**, Scheme 1B), in which internal methyl groups have been replaced by isobutenyl units in order to stabilize the CPD form, could be converted in toluene by illumination at λ_ex_ = 551 nm with a remarkable quantum yield (Φ_c–o_) of 66%.^46^ More recently, we have reported that a suitable functionalization of the DHP core by pyridinium group(s) may drastically enhance the quantum yield of the photo-ring-opening reaction while lowering the energy of the incident wavelength.^47−50^ In particular, the monosubstituted compound **DHPPy^+^** (Scheme 1B) can be isomerized with a quantum yield Φ_c–o_ = 9.3% at λ_ex_ = 660 nm in CH_3_CN. Such performance was explained by the electron-withdrawing character of the pyridinium group inducing a charge transfer character to the excited states thus allowing a photoisomerization at lower energies, directly from the lowest singlet excited state (S_1_).^51^ These few examples have demonstrated that DHP/CPD derivatives can act as competitive photochromic couples. However, in all the above-cited examples, the ring-closure reactions (CPD → DHP) were achieved using UV light illumination.

Following these previous studies, and driven by preliminary calculations, we chose to rationally synthesize and investigate the benzo-fused pyridinium dimethyldihydropyrene derivative **BDHPPy**^+^ (Scheme 1C), associating both a benzo[*e*]-fused DHP and a methylpyridinium unit. Indeed, such a donor–acceptor system was expected to operate using illumination at low energies while improving the photo-ring-opening process. In addition, if the pyridinium group was introduced because of its electronic effects, its second important role was to increase the hydrophilicity of the system due to its positive charge.

## Results and Discussion

### Syntheses

**Scheme 2:**
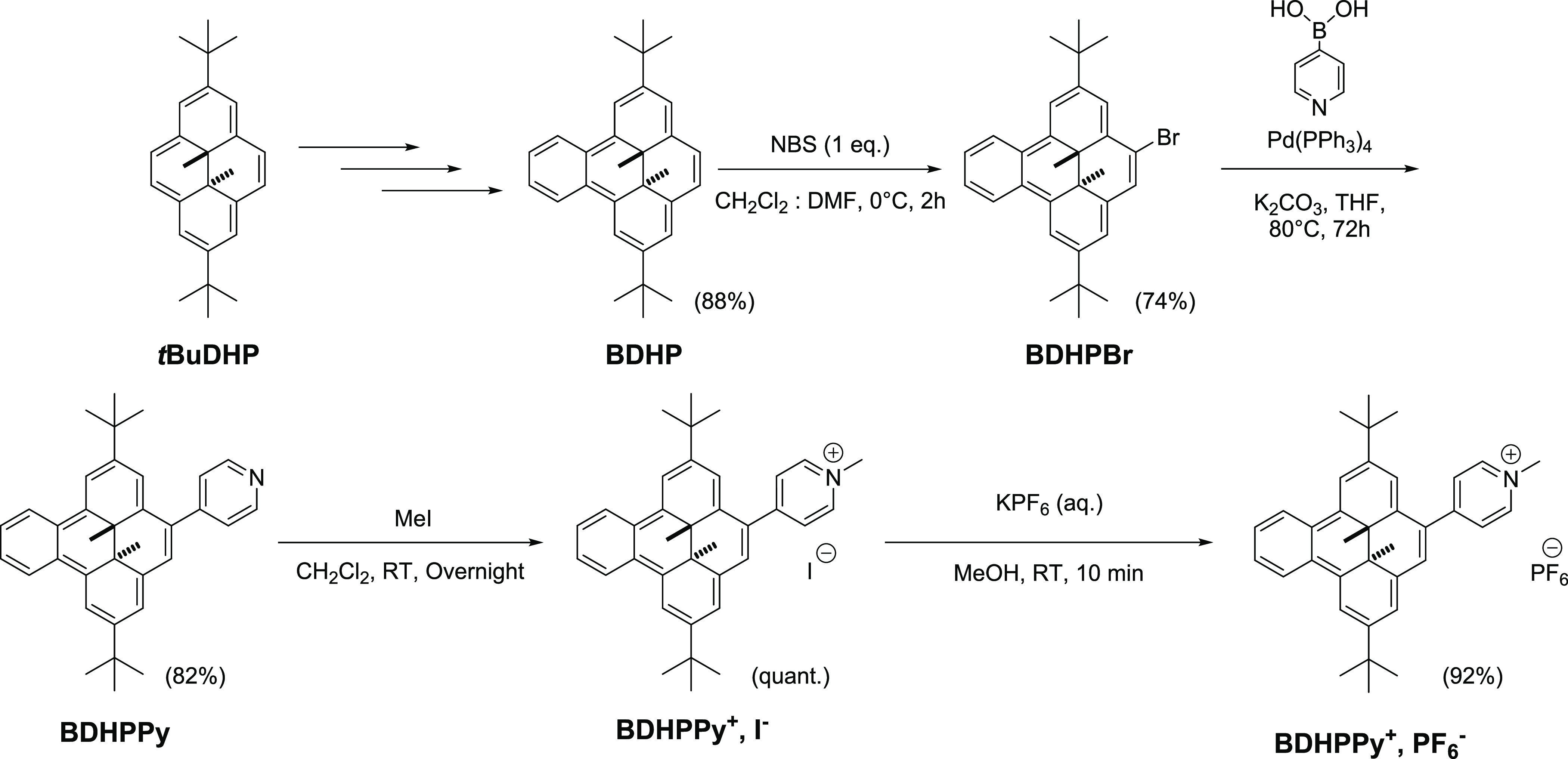
Synthetic Procedure for the Preparation of **BDHPPy^+^**, **I^–^** and **BDHPPy^+^**, **PF_6_^–^**

The target photochromic compound **BDHPPy^+^**, as its **I^–^** and **PF_6_^–^** salts, was prepared following the synthetic route summarized in Scheme 2. The benzo[*e*]-fused compound (**BDHP**) was first prepared in three steps from 2,7-di-*t*-butyl-*trans*-10*b*,10*c*-dimethyl-10*b*,10*c*-dihydropyrene (***t*BuDHP**), following the procedure reported by Mitchell and Ward.^52^**BDHP** was then brominated by the reaction of one molar equivalent of *N*-bromosuccinimide (NBS) in a mixture of DMF and CH_2_Cl_2_ (yield: 74%).^53^ The mono-brominated compound **BDHPBr** was then subjected to a Suzuki–Miyaura coupling reaction in the presence of 4-pyridylboronic acid and tetrakistriphenylphosphinepalladium(0) as a catalyst, and **BDHPPy** was obtained in 82% yields. This compound was then methylated using an excess of methyl iodide providing quantitatively **BDHPPy^+^**, **I^–^** that was isolated as a red-brown powder. The latter compound could then be subjected to an anion metathesis using a saturated aqueous solution of KPF_6_ to afford **BDHPPy^+^**, **PF_6_^–^** (92%).

The structures of these compounds were confirmed by ^1^H and ^13^C nuclear magnetic resonance (NMR) spectroscopy methods as well as mass spectrometry analyses (see the Experimental Section and the Supporting Information).

### Characterization of **BDHPPy^+^** by ^1^H NMR and UV/Vis Spectroscopy and Theoretical Calculations

The photochromic properties of **BDHPPy^+^** were investigated with **I^–^** and **PF_6_^–^** as counter-anions. Indeed, the two salts exhibit different solubilities: whereas **BDHPPy^+^**, **PF_6_^–^** was lipophilic and soluble in a wide range of organic solvents, **BDHPPy^+^**, **I^–^** was found to be water-soluble. Using this interesting feature, the photochromic properties of the **BDHPPy^+^** moiety could be investigated both in organic solvents and in water.

**Figure 1:**
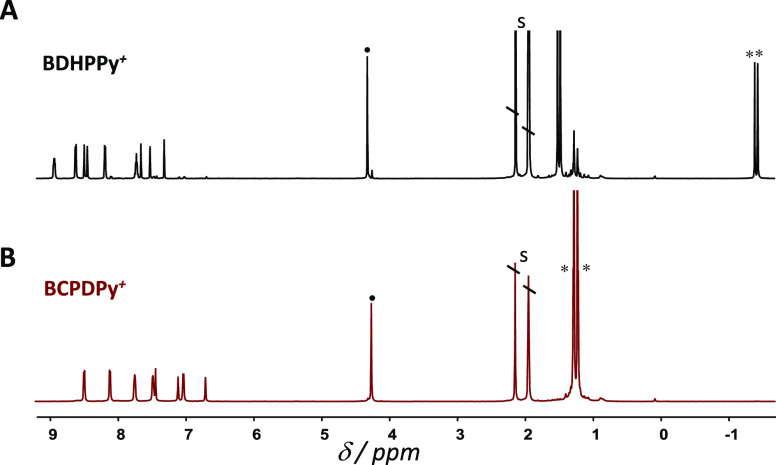
^1^H-NMR spectra of (A) **BDHPPy^+^** and (B) **BCPDPy^+^** in CD_3_CN. ^•^ and * indicate the signals of N^+^–CH_3_ and internal methyl groups, respectively. S: solvent peaks.

The proton NMR spectrum of **BDHPPy^+^**, **PF_6_^–^** recorded in CD_3_CN (Figure 1A), corroborates the structure of the closed isomer. Resonances of the aromatic protons of the DHP skeleton are seen at low field, between 7 and 9 ppm, and the signal of the N^+^–CH_3_ group appears as a singlet at 4.3 ppm. However, the more relevant signal showing that the system is in its closed state corresponds to the two singlets of the internal methyl protons that are seen at −1.39 and −1.44 ppm. Such shift is attributed to the ring current of the delocalized π-electrons of the DHP unit and constitutes a real signature of the closed isomer.^54,55^

**Figure 2:**
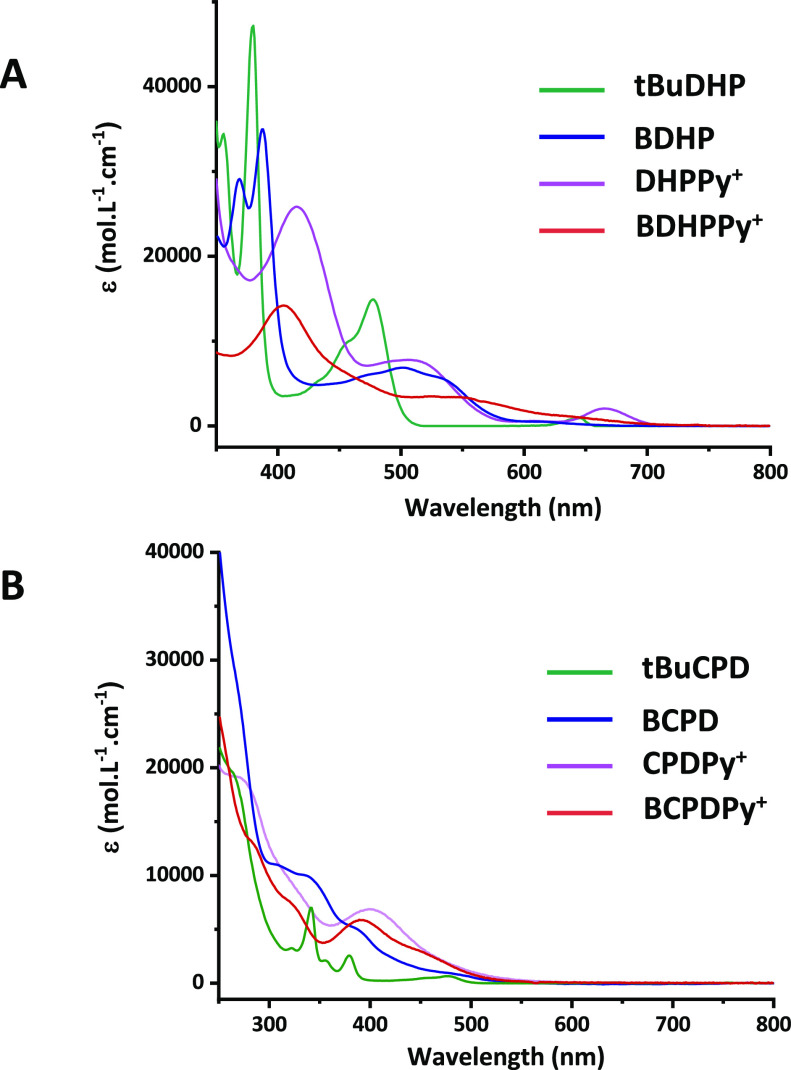
UV/vis spectra of (A) ***t*BuDHP** (green curve, in cyclohexane), **BDHP** (blue curve, in cyclohexane), **DHPPy^+^** (pink curve, in CH_3_CN), and **BDHPPy^+^**, **PF_6_^–^** (red curve, in CH_3_CN) and (B) their corresponding open isomers (with the same color code). Note: traces of ***t*BuDHP** are remaining in the spectrum of ***t*BuCPD** because of the thermal return reaction.

The UV/vis absorption properties of **BDHPPy^+^** under its PF_6_^–^ and I^–^ forms have been measured in acetonitrile and water, respectively, and were compared to those of its parent derivatives, i.e., ***t*BuDHP**, **BDHP**, and **DHPPy^+^** (experiments were performed in cyclohexane or acetonitrile, depending on the solubilities of the compounds). Data are given in Figure 2A (see also Figure S15). The different compounds absorb in the visible region and their absorption spectra exhibit main bands attributed to π–π* transitions. Compared to ***t*BuDHP**, the spectra of **BDHP**, **DHPPy^+^,** and **BDHPPy^+^** appeared bathochromically shifted. In particular, **BDHPPy^+^** absorbs in all the visible range up to λ = ∼700 nm, which is unusual for benzo[*e*]-fused DHP derivatives and can be explained by the presence of the pyridinium unit. Importantly, the visible ranges of the absorption spectra of **BDHPPy^+^** were very close in organic solvents and water. In particular, no significant solvatochromic effects were observed suggesting that the nature of the solvent and of the counter-anion should not play an important role in the photochromic properties of the system.

Time-dependent-density functional theory (TD-DFT) calculations of vertical transition energies for **BDHP** and **BDHPPy^+^** provided results that are in line with the experimental observations. As illustrated by the natural transition orbitals displayed in Table 1, the S_0_ → S_1_ electronic transition is characterized by a significant charge transfer character from the benzo[*e*]-fused-DHP core to the pyridinium electron-withdrawing group in **BDHPPy^+^**. As a result, the transition energy decreases by 0.22 eV compared to the **BDHP** compound, accounting for the red-shift of the absorption observed experimentally upon introducing the pyridinium group (see also Figure S16 for the simulated spectra).

**Table 1:**
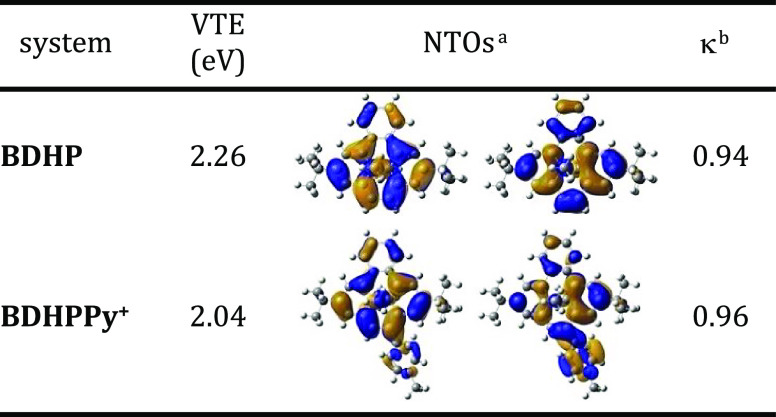
S_0_ → S_1_ Vertical Transition Energies in eV Computed at TD-DFT Level and Pair of Natural Transition Orbitals (NTOs) Characterizing the Electronic Transitions

a Main pair of natural transition orbitals reflecting the dominant particle-hole excitation character.

b Associated natural transition orbital eigenvalue.

### Investigation of the Photochromic Properties of **BDHPPy^+^**

**Figure 3:**
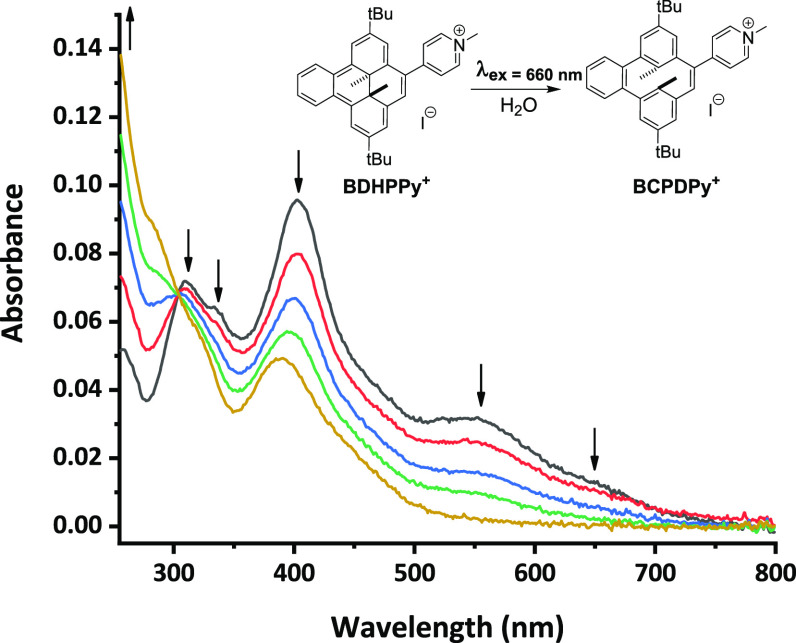
UV/vis absorption spectra evolution of a solution of **BDHPPy^+^**, **I^–^** in H_2_O during irradiation with visible light (λ_ex_ = 660 nm). Time between two consecutive spectra: 30 s. The isomerization of **BDHPPy^+^**, **I^–^** to **BCPDPy^+^**, **I^–^** is observed.

The possibility of photoisomerization of **BDHPPy^+^** to its corresponding cyclophanediene form was demonstrated by absorption spectroscopy, both in organic and in aqueous solvents. When a solution of **BDHPPy^+^** was irradiated at λ_ex_ = 660 nm, a color change from red to bright yellow was rapidly observed. This effect corresponds to a disappearance of the visible absorption bands of the **BDHPPy^+^** isomer which is accompanied by the growth of new bands in the UV range (around 280 nm), combined with a remaining absorption at ∼402 nm that extends up to 500 nm. This evolution is given in Figure 3, and the absorption spectra of the other investigated open isomers are given in Figure 2B for comparison. The open **BCPDPy^+^** isomer absorbs at lower energies compared to the cyclophanediene forms that do not contain pyridinium group, which may indicate that its photoclosing process (open to closed form) can be envisaged using irradiation with visible light.

In the same manner, when the NMR tube containing **BDHPPy^+^** was illuminated with visible light at λ_ex_ = 660 nm using monochromatic LEDs, a clean and fast photoconversion to the corresponding open form was observed. Indeed, during irradiation, NMR signals of the starting product progressively disappeared in favor of new NMR peaks attributed to the **BCPDPy^+^** isomer (Figures 1B and S13). In particular, signals of the internal methyl protons were strongly shifted at lower fields (from ∼−1.4 to ∼+1.2 ppm), in accordance with the breaking of the central C–C bond. At the end of the experiment (upon few minutes of light exposure under our experimental conditions), the closed form was quantitatively converted.

The reversibility of the photoisomerization process was then investigated. A solution of the open isomer **BCPDPy^+^** was prepared and exposed to different wavelengths in order to find the best experimental conditions. It was found that a fast photo-ring-closing reaction could be reached by irradiation with blue light at λ_ex_ = 470 nm (vide infra). During illumination, a color change from yellow to red was observed, and the UV/vis absorption and proton NMR spectra of the closed isomer were rapidly recovered. At the end of the experiment, upon few minutes of illumination, a 95% conversion was recorded (determined by ^1^H-NMR, see Figure S14). Again, similar behaviors were observed in CH_3_CN (PF_6_^–^ form) and in H_2_O (I^–^ salt) as solvents.

**Figure 4:**
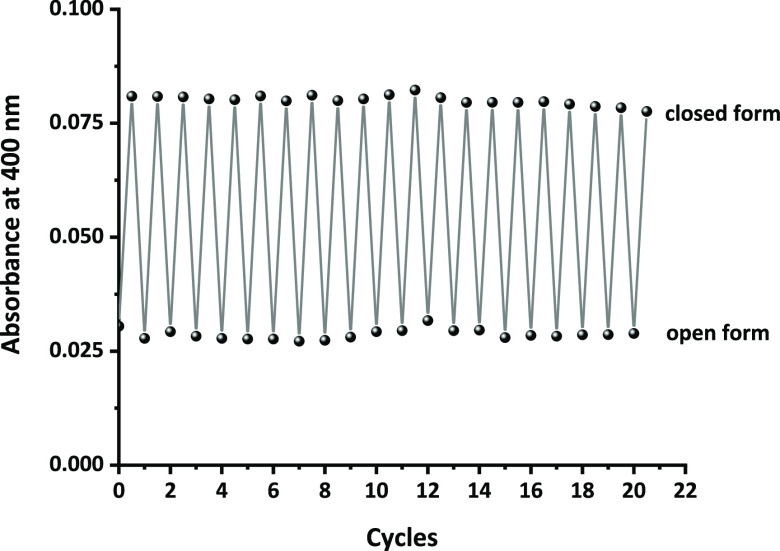
Fatigue resistance of the **BDHPPy^+^**/**BCPDPy^+^** photochromic couple in pure water. Absorbances at 400 nm upon alternate illuminations at 470 and 660 nm are indicated.

The reversibility of the **BDHPPy^+^**/**BCPDPy^+^** photochromic couple as well as its stability was further confirmed by measurement of the fatigue resistance (see Figures 4 and S17). In this experiment, a solution of **BDHPPy^+^** was alternatively irradiated with light at 660 and 470 nm at room temperature and under an air-atmosphere. The behavior of the system was followed by absorption and NMR spectroscopy methods. Using the obtained data, cyclabilities (*Z*_50_)^56^ above 4000 were found both in organic and aqueous solvents. This result demonstrates the remarkable reversibility and stability of the system.

It must be emphasized that all these experiments can be conducted under anaerobic or aerobic conditions and that no differences were observed. Indeed, previous studies have shown that DHPs, and in particular those substituted by pyridinium groups, may act as a O_2_-photosentitizer and can also store and release singlet dioxygen (^1^O_2_) through the formation of endoperoxide derivatives.^47,57^ In the present system, no reactivity with O_2_ was observed, and this feature is clearly corroborated by our calculations that show that the energy transfer reaction (eq 1) between the present photochromic system and dioxygen is unfavorable (Table 2). Indeed, the reaction energies for this energy transfer process are positive for **BDHP** and **BDHPPy^+^**, whereas they are negative for ***t*BuDHP** and **DHPPy^+^**. These results support nicely the experimental observations, as ^1^O_2_ is only produced with ***t*BuDHP** and **DHPPy^+^**. The absence of the ^1^O_2_ generation and subsequent endoperoxide formation in **BDHPPy^+^** clearly contribute to the excellent fatigue resistance of this derivative.
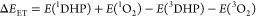
1

**Table 2:** Reaction Energies for the Energy Transfer (Δ*E*_ET_ in kcal/mol) between DHP Isomers and Dioxygen in Various DHP Derivatives

system	Δ*E*_ET_	solvent	expt.^a^
***t*BuDHP**	–8.3	cyclohexane	yes
**DHPPy^+^**	–5.3	acetonitrile	yes
**BDHP**	0.3	cyclohexane	no
**BDHPPy^+^(PF_6_^–^)**	2.5	acetonitrile	no
**BDHPPy^+^(I^–^)**	2.5	water	no

a Experimental observations of ^1^O_2_ production.

### Investigation of the Thermally Triggered Ring-Closing Reaction (Open Form → Closed Form)

In addition to the optically controlled process, the closing process of CPD derivatives can be generally achieved thermally.^58^ When solutions of **BCPDPy**^+^ under its PF_6_^–^ (in CH_3_CN) or its I^–^ (in water) salts were heated under dark conditions, the closed-ring isomer could be progressively and quantitatively generated (see Figures S20–S23). Different temperatures were tested and, considering a first-order kinetic, activation energies *E*_a_ of 24 ± 3 and 22 ± 2 kcal/mol were determined for **BCPDPy^+^**, PF_6_^–^, and **BCPDPy^+^**, I^–^, respectively. The computed energy barrier based on broken-symmetry DFT calculations was found at 21.5 kcal/mol, in good agreement with the experimental values and close to most CPD derivatives reported in the literature.^59^ In addition, a half lifetime (*t*_1/2_) of 19.4 ± 0.3 h was found for **BCPDPy**^**+**^, PF_6_^–^ in CH_3_CN at 37 °C (14.8 ± 0.3 h for **BCPDPy^+^**, I^–^ in water).

### Photoswitching Efficiencies and Mechanisms of the **BDHPPy^+^**/**BCPDPy^+^** Couple

**Figure 5:**
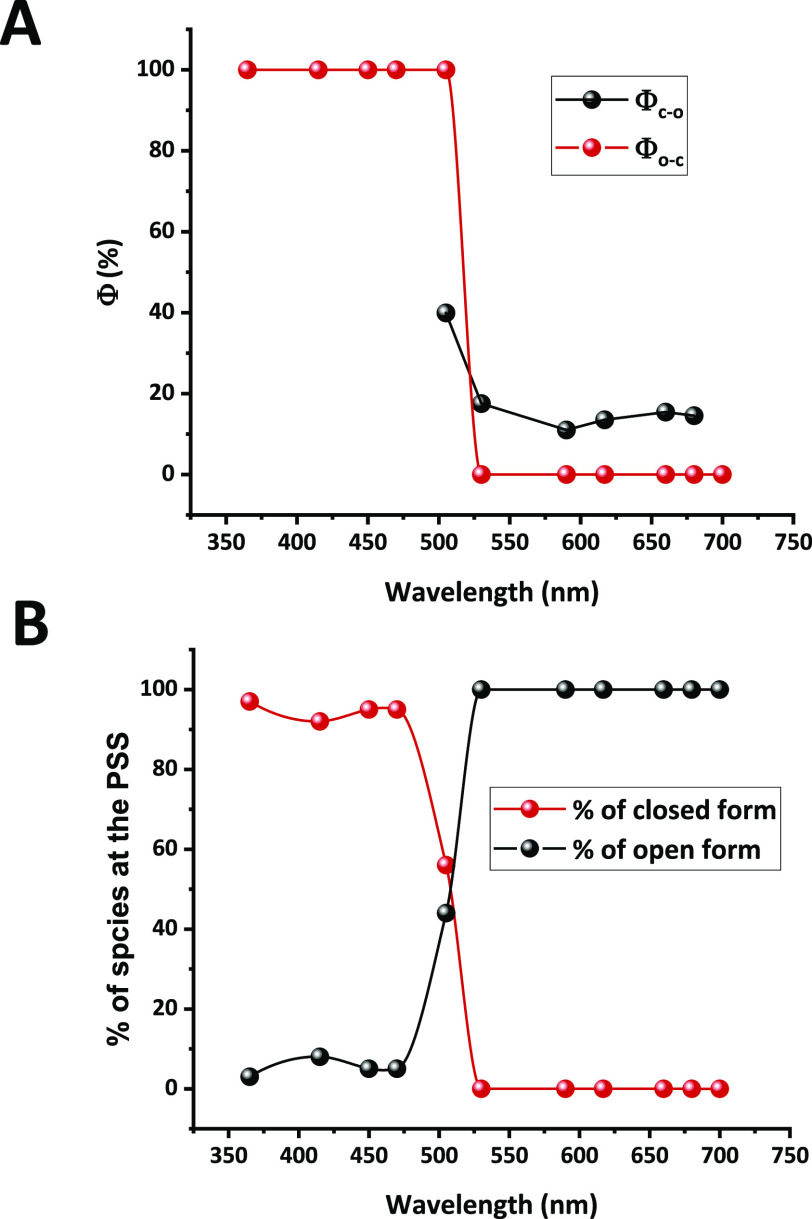
(A) Quantum yields of the photo-ring-opening (Φ_c–o_: black balls) and photo-ring-closing (Φ_o–c_: red balls) processes for the **BDHPPy^+^**/**BCPDPy^+^** photochromic couple in water as a function of the irradiation wavelengths. (B) Conversion percentages of open (black) and closed (red) forms at the PSS for different wavelengths. Note: Φ_c–o_ are not given below 500 nm due to high uncertainties.

To quantify the performances of the **BDHPPy^+^**/**BCPDPy^+^** photochromic couple and to determine the optimal experimental conditions for the photoisomerization reactions, we measured the quantum yields of the photo-ring-opening (Φ_c–o_) and photo-ring-closing (Φ_o–c_) processes at different wavelengths. For this, 10^**–**6^–10^**–**5^ M solutions of the open or closed isomers were irradiated at specific wavelengths using monochromatic LED beams, and the conversions were followed by absorption spectroscopy. Quantum yields were then determined upon fitting the data using the kinetic model reported by Brown and Maafi.^60,61^ The Φ_c–o_ and Φ_o–c_ values are depicted in Figure 5A. In addition, the values of the conversion yields at the PSS were determined at different wavelengths and are represented in Figure 5B.

As seen in Figures 5A,B (see also Tables S1 and S2), the ring-opening reaction can be realized quantitatively by illumination at λ_ex_ ≥ 525 nm, with quantum yields around 15%. In particular, values of Φ_c–o_ = 15.4 and 14.5% were found when **BDHPPy^+^**, **I^–^** was irradiated at λ_ex_ = 660 and 680 nm, respectively in water (20.0% was reached for **BDHPPy^+^**, **PF_6_^–^** in CH_3_CN at λ_ex_ = 680 nm). Such yields are much higher than those obtained for **BDHP** (7.4% at λ_ex_ = 389 nm^46^ and 2.4% at λ_ex_ = 505 nm) and **DHPPy^+^** (9.3% at λ_ex_ = 660 nm). It should be noted that higher quantum yields could be measured at lower wavelengths of excitation (around 500 nm), but such conditions are not optimal because significant amounts of the two isomers (i.e., low PSS) are obtained. For this reason, illumination of the closed isomer in the 660–680 nm range represents the best compromise for the ring-opening process.

Concerning the ring-closing process, quantum yields close to 100% can be found up to ∼500 nm. Such high Φ_o–c_ values are often observed for DHP/CPD systems, but only in the UV part of the spectrum. In the present system, the best compromise for the open to closed form conversion was found at λ_ex_ = 470 nm, i.e., blue light illumination, where a quantitative quantum yield accompanied by a high PSS can be reached, in organic or aqueous media (Table 3).

**Table 3:** Quantum Yields for the Photo-Opening Process (Φ_c–o_) for ***t*BuDHP**, **DHPPy^+^**, **BDHP,** and **BDHPPy^+^**^a^

compounds	quantum yields
	Φ_c–o_ [%] (λ_ex_, nm)
***t*BuDHP**	0.00 (660)
	0.08 (470)
**DHPPy^+^**	9.30 (660)
**BDHP**	0.00 (660)
	2.40 (505)
**BDHPPy^+^**	16.50 (660)

a For solubility reasons, ***t*BuDHP** and **BDHP** were studied in cyclohexane and **DHPPy^+^** and **BDHPPy^+^** were investigated in acetonitrile.

**Figure 6:**
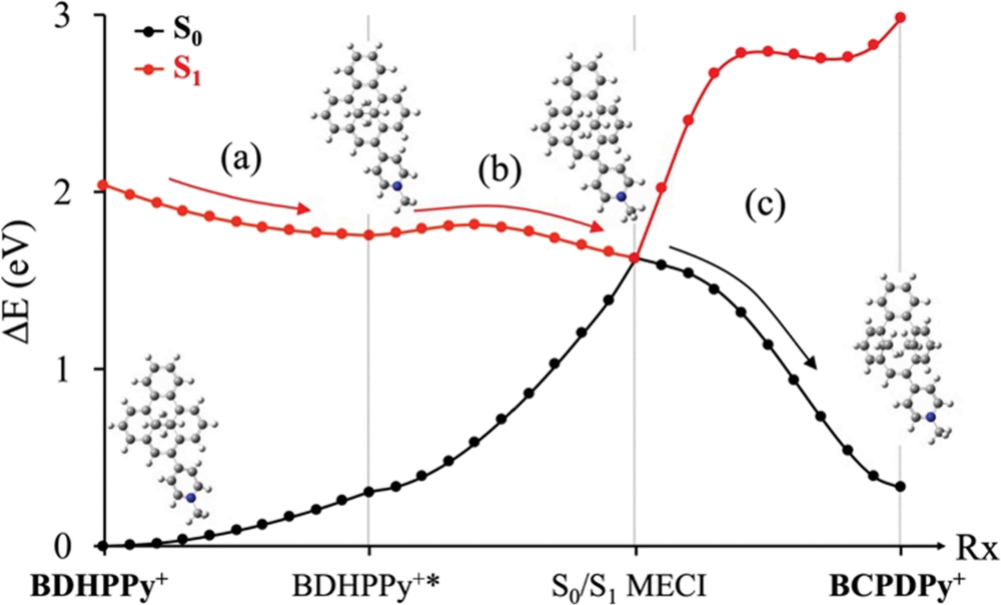
Photoisomerization pathway from ground-state **BDHPPy^+^** to **BCPDPy^+^**: (a) excited-state relaxation on S_1_ to BDHPPy^+^*, (b) weakly activated process to reach the photochemical funnel (S_0_/S_1_ MECI), and (c) nonradiative deactivation to S_0_ and ground-state relaxation to **BCPDPy^+^**. All the Cartesian coordinates for the molecular structures are provided in the Supplementary Information.

The theoretical investigation at the spin-flip-time-dependent-density functional theory (SF-TD-DFT) level of the photoisomerization mechanism on the lowest S_1_ excited state of the **BDHPPy^+^** compound accounts for the observed high ring-opening quantum yield. The excited-state potential energy profile (Figure 6) shows a very favorable relaxation path on the S_1_ state toward the photochemical funnel, that is the S_0_/S_1_ minimum energy conical intersection (MECI) responsible for the nonradiative decay down to S_0_ on the way to the CPD photoproduct formation. A low potential energy barrier (0.3 kcal/mol) is found on the way to the MECI and this crossing is located 3 kcal/mol below the excited-state intermediate BDHPPy^+^*, suggesting an efficient and fast photoisomerization process. This is reminiscent of the potential energy profile found in a recently studied donor–acceptor DHP derivative.^47^ However, in the present system, the excited-state barrier to access the S_0_/S_1_ MECI is even smaller, and the MECI itself is lower in energy relative to the excited-state intermediate. This provides a possible argument to explain the higher photoisomerization quantum yield of **BDHPPy^+^** (Φ_c–o_ = 16.5% at λ_ex_ = 660 nm in CH_3_CN) compared to that of the donor–acceptor DHP (Φ_c–o_ = 13.3% at λ_ex_ = 660 nm in CH_3_CN).

### Electrochemical Study of the **BDHPPy^+^**/**BCPDPy^+^** System

The different isomers of a photochromic couple usually exhibit distinct and specific redox behaviors, and electrochemical techniques represent thus very good tools to read the state of the system (output signal). In addition, switching processes may also be induced by electrical inputs, which is particularly attractive for the development of multi-stimuli-responsive molecular materials. For example, electrically triggered ring-closing and ring-opening reactions are well documented for photochromic systems based on dithienylethenes,^37,38,62^ but they remain rare with dimethyldihydropyrene derivatives. The group of Mitchell reported a Ru(II) complex incorporating a benzocyclophanediene unit in which the closing process was induced by reduction of the Ru center.^59^ Nishihara and co-workers^63,64^ and our group^51^ respectively demonstrated oxidation-triggered isomerization of benzocyclophanedienes and pyridinium-cyclophanediene to their corresponding dimethyldihydropyrene isomers. In this work, the redox activities of **BDHPPy^+^** and **BCPDPy^+^** as their hexafluorophosphate salts were investigated by cyclic voltammetry (CV) under an inert atmosphere in acetonitrile containing tetra-*n*-butylammonium hexafluorophosphate (TBAPF_6_, 0.1 M) as the supporting electrolyte.

**Figure 7:**
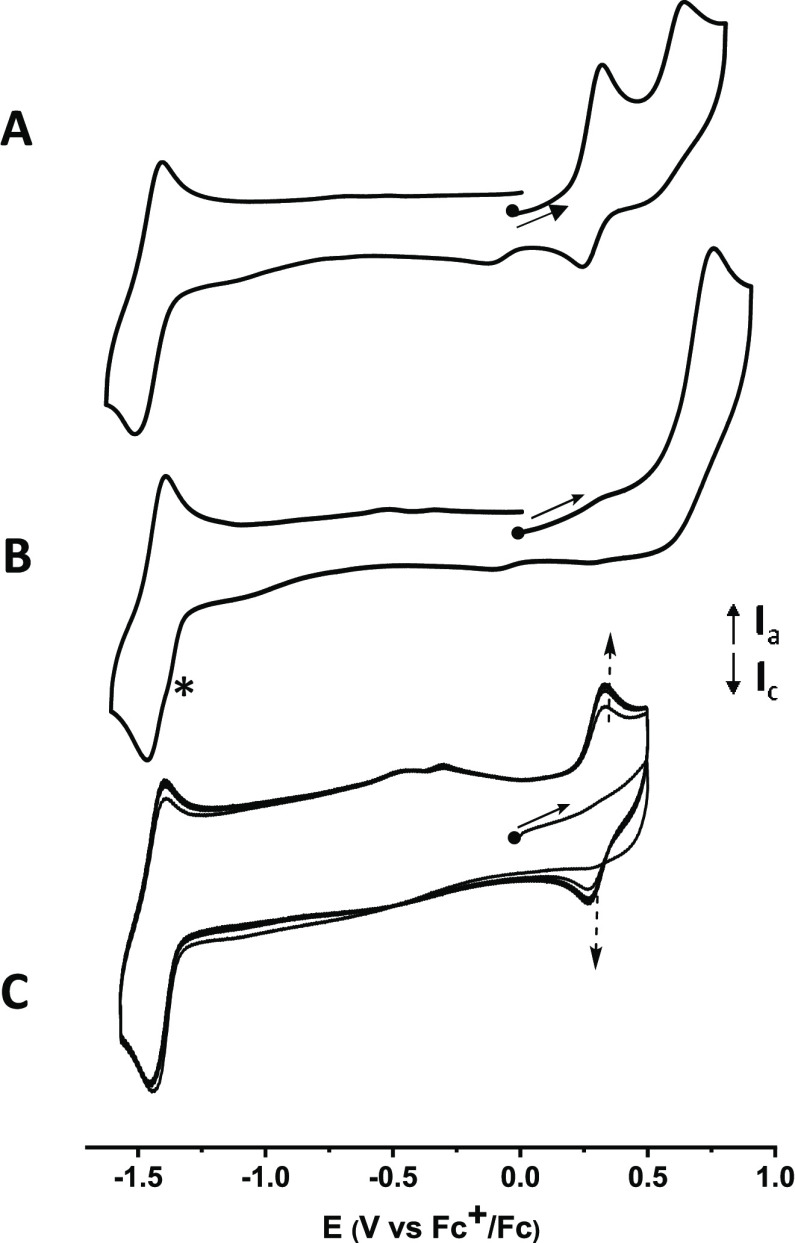
Cyclic voltammograms of (A) **BDHPPy^+^** and (B) **BCPDPy^+^**. * indicates a reduction signal centered on the pyridinium unit in the open form. (C) Repeated cyclic voltammograms of the photogenerated solution of **BCPDPy^+^**. [Conc.] ∼ 1 mmol in 0.1 M TBAPF_6_/CH_3_CN. Scan rate: 100 mV/s.

The CV curve of **BDHPPy^+^** recorded at a scan rate of 100 mV/s (Figure 7A) displays a first reversible wave at *E*_1/2_ = +0.37 V (Δ*E*_p_ = 70 mV) versus the ferrocenium/ferrocene reference couple (Fc^+^/Fc), attributed to the monoelectronic oxidation of the DHP core (DHP^+·^/DHP couple). This signal is followed by an irreversible peak at *E*_pa_ = +0.59 V (DHP^2+^/DHP^+·^) accompanied, during the reverse scan by a weak and ill-defined reduction wave at ∼−0.1 V. This irreversible behavior was attributed to undetermined coupled chemical reaction(s) that follows the electron transfer. In addition, a reversible reduction wave corresponding to the monoelectronic reduction of the pyridinium group is seen at *E*_1/2_ = −1.51 V (Δ*E*_p_ = 80 mV). Another reduction peak is also observed at *E*_pc_ = −1.96 V, attributed to the irreversible reduction of the DHP core.

The solution of **BDHPPy^+^** was then illuminated at 660 nm, and the CV curve of the photogenerated **BCPDPy^+^** compound was recorded (Figure 7B). In accordance with the formation of the open isomer, a unique oxidation peak corresponding to the irreversible oxidation of the CPD center was seen at *E*_pa_ = +0.70 V. When scanning toward low potentials, a first irreversible wave is seen at *E*_pc_ = −1.44 V (see * in Figure 7B). This signal is then followed by a reversible reduction at −1.51 V, i.e., at the same potential than the reduction of **BDHPPy^+^**. When a second cycle was recorded between −1.2 and −1.55 V, the signal at −1.44 V disappeared (see Figure S18). Such behavior indicates that the closed-ring isomer is spontaneously and rapidly formed (at the CV timescale) upon reduction of the open form. This hypothesis was fully confirmed when voltammetric scans were repeated between −1.60 and +0.50 V (Figure 7C): the CV curve of the closed form is immediately seen upon reduction of the open isomer.

**Figure 8:**
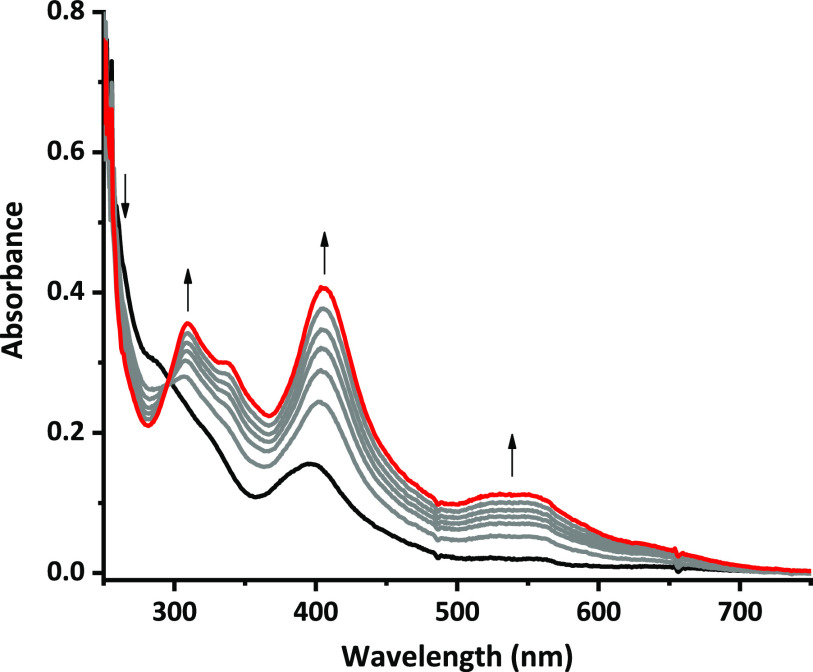
UV/visible spectral changes during reduction of **BCPDPy^+^**. Thick black line: initial spectrum. Red line: final spectrum after passage of 0.06 electron per molecule.

To further demonstrate this process, spectroelectrochemical experiments were performed by following in situ the evolution of the UV/vis spectra of a solution of **BCPDPy^+^** during a potentiostatic electrolysis at −1.35 V (Figure 8). The UV/vis absorption signals of the closed-ring isomer rapidly grew, and the spectrum characteristic of a large amount of **BDHPPy^+^** was obtained after a reduction corresponding to only ∼0.06 e^–^ per molecule.

These results reveal that the redox induced ring-closure reaction involves a catalytic mechanism that can be represented as follows (eqs 2−4):
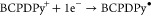
2
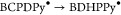
3
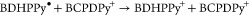
4

The first step corresponds to the reduction of **BCPDPy^+^** (eq 2). The electrogenerated **BCPDPy^•^** then spontaneously and rapidly isomerizes (at least at the CV time scale) to produce the corresponding closed state **BDHPPy^•^** (eq 3). This step is further supported by broken-symmetry DFT calculations which place energetically **BCPDPy^•^** 8 kcal/mol above **BDHPPy^•^** with a ring-closure potential energy barrier of only 13 kcal/mol. **BDHPPy^•^** is then oxidized by the cyclophanediene isomer **BCPDPy^+^** (eq 4) and the generated **BDHPPy^•^** then re-enters the catalytic cycle. This system is thus of particular interest for the conception of “dual-mode-responsive systems”, in which switching processes can be induced with optical and electrical stimuli.

## Conclusions

We have designed and investigated an all-visible photochromic system based on a benzo[*e*]fused dimethyldihydropyrene unit substituted by a pyridinium group. This molecular switch is able to be photoisomerized upon illumination in the visible range (680/470 nm) between its open and closed forms with high photostationary states (PSS near to 100%) and quantum yields. Remarkably, depending on the nature of its counter anion, this system is able to operate in organic and aqueous solvents, with a high fatigue resistance under aerobic conditions. In addition, the closing reaction can be triggered by electrical inputs. Finally, one great asset of this simple photoswitch is with no doubt the possibility to easily replace the simple methyl group of the pyridinium arm by functional units such as anchoring moieties or targeting agents, in order to build multifunctional systems, while keeping its efficient photochromic properties.

## Methods

### General Procedures and Instrumentation

All solvents were purchased and used as received except THF that was distillated over sodium/benzophenone under argon. Water was purified by reverse osmometry with an Elgastat purification system (5 MΩ·cm). Organic and inorganic reagents used in the procedures were purchased on Aldrich, Acros, Fluorochem or TCI Europe and used without further purification. All evaporations were carried out under reduced pressure with a rotatory evaporator, and all organic extracts were washed with water and dried over MgSO_4_. Isocratic chromatographic columns were executed using flash chromatography, and gradient chromatographic columns were realized using dried vacuum column chromatography (DCVC).^65^ Flash column chromatography refers to Merck Kieselgel silica gel, 40–63 μm and DCVC Kieselgel refers to Merck 14–40 μm. Melting points were measured thanks to capillary tubes on a Büchi B-545 device. Infrared spectra were recorded on a PerkinElmer Spectrum Two Fourier transform infrared spectrometer using attenuated total reflexion modes. ^1^H NMR and ^13^C NMR spectra were recorded on a Bruker Avance 500 or 400 MHz spectrometer in CDCl_3_ or CD_3_CN. Chemical shifts are calibrated to residual solvent peaks. Coupling constant values (*J*) are given in hertz and chemical shifts (δ) in ppm. High-resolution mass spectrometry analyses were conducted using the HRMS, Bruker maXis mass spectrometer and were performed in positive electrospray ionization (ESI^+^) at the DCM mass facility.

### Spectroscopy

Absorption spectra were recorded using a Varian Cary 60 Scan UV/visible spectrophotometer equipped with a temperature controller unit. Absorption measurements over the spectral range from 250 to 800 nm were carried out in Hellma quartz suprasil cells having an optical path length of 1 cm.

### Electrochemical Experiments

Electrochemical measurements (cyclic voltammetry, CV) were conducted under an argon atmosphere (argon stream) with a standard one-compartment, three-electrode electrochemical cell using a CH-Instrument 660B potentiometer. Anhydrous CH_3_CN was used as the solvent and tetra-*n*-butylammonium hexafluorophosphate (TBAPF_6_, 0.1 M) was used as the supporting electrolyte. CH-Instrument vitreous carbon (diameter = 3 mm) working electrodes were used for CV experiments. Electrodes were polished with 1 μm diamond paste (Mecaprex Presi) prior to each recording. The counter-electrode was a platinum wire immerged directly in the solution. A CH-Instrument AgNO_3_/Ag (10^–2^ M + TBAP 10^–1^ M in CH_3_CN) electrode was used as a reference electrode. The potential of the regular ferrocenium/ferrocene (Fc^+^/Fc) redox couple was added at the end of each experiment and was used as the internal reference. CV curves were recorded at different scan rates. An automatic ohmic drop compensation procedure was systematically implemented prior to recording CV data. Electrolysis experiments were performed at controlled potential using a Pt plate (2 cm^2^). All the experiments were carried out at room temperature.

### Irradiation Procedures

Irradiation experiments in solution were performed under an inert atmosphere using a Jacomex glove box with carefully degassed solvents. The samples were irradiated in different cells (classical UV/visible quartz cells, NMR tubes or electrochemical cells). The concentration used for UV/visible spectroscopy and NMR experiments was ∼10^–5^ M and 2 mg/mL, respectively, unless otherwise stated. The samples were stirred and kept at 277 K during irradiation in order to limit the thermal back isomerization. The visible light irradiations were carried out with various mounted LEDs from Thorlab: 365 nm (M365L3, FWHM = 9 nm, 880–1290 mW), 415 nm (M415L4, FWHM = 14 nm, 1310–1550 mW), 450 nm (M450LP1, FWHM = 18 nm, 1850–2100 mW), 470 nm (M470L5, FWHM = 28 nm, 809–1162 mW), 505 nm (M505L4, FWHM = 37 nm, 400–520 mW), 530 nm (M530L4, FWHM = 35 nm, 370–480 mW), 590 nm (M590L4, FWHM = 15 nm, 230–300 mW), 617 nm (M617L3, FWHM = 18 nm, 600–650 mW), 660 nm (M660L4, FWHM = 20 nm, 940–1050 mW), 680 nm (M680L4, FWHM = 22 nm, 180–210 mW), 700 nm (M700L4, FWHM = 20 nm, 80–125 mW) in combination of a Thorlab DC2200 led driver and an adjustable collimation adapter (SM2F32-A).

The isomerization process was monitored either by UV/visible or by ^1^H NMR and it was considered that the PSS was reached when no evolution was observed in three consecutive intermediate spectra or after a long period of irradiation. Quantities of residual closed isomers were determined without ambiguities in the negative region by the integration of their characteristic resonance peaks of the internal methyl groups by ^1^H NMR, or by absorption spectroscopy using the Beer–Lambert law above 600 nm (where only the closed form absorbs). The photoisomerization fatigue was investigated by UV/visible spectroscopy. Spectra were recorded upon repeated irradiation cycles at λ_ex_ = 470 nm and 660 nm alternatively. The irradiation times were chosen in order to reach the maximum conversion between photostates (the minimum times were determined from separated UV/visible experiments). Cyclabilities (*Z*_50_) were then calculated following the reported method.^56^

### Quantum Yield Measurements

Quantum yields for the photoinduced ring-opening process (Φ_c–o_: closed form to open form and Φ_o–c_ open to closed form) were determined by illumination of solutions of the open and closed isomers at 277 K using monochromatic LEDs at specific wavelengths. Samples were placed at 4 cm from the irradiation source. The light power was measured with a Newport (818-SL) photodetector and the photoconversion was followed by UV/visible absorption spectroscopy. Data were extracted by fitting of the experimental curves using the kinetic model reported by Maafi and Brown.^60,61^

### Computational Details

The computational strategy follows that already described in our previous work^47^ on a related system and is described herein. DFT and its time-dependent version (TD-DFT) were used to perform calculations on the ground and excited states, respectively, of **BDHP** and **BDHPPy^+^**. Ground-state geometry optimizations were carried out with the hybrid B3LYP functional,^66^ while excited states were computed and optimized with the long-range corrected hybrid ωB97X-D functional^67^ within linear-response TD-DFT. Pople’s 6-311G(d,p) basis set was used throughout.^68^ All calculations were performed with the corresponding solvent used in the experiment (i.e., cyclohexane for **BDHP**, and acetonitrile for **BDHPPy^+^**) within the integral equation formalism polarizable continuum model (IEFPCM).^69^ Vertical absorption transition energies were computed using linear-response nonequilibrium solvation. Note that within the crude vertical approximation, deviations of about 0.25 eV are rather common for valence excited states of organic molecules computed with TD-DFT.^70^ NTOs^71^ for the S_0_ → S_1_ electronic transition were generated in order to compare the nature of the excited states involved between **BDHP** and **BDHPPy^+^**.

The transition state for the thermal **BCPDPy^+^** to **BDHPPy^+^** conversion was located using broken-symmetry DFT due to the biradical (open-shell singlet) nature of this species. To account for the spin contamination, spin-projected energies were computed with the approximate spin-correction procedure proposed by Yamaguchi and coworkers.^72,73^ Reduced species **BDHPPy**· and **BCPDPy**· and the transition state connecting them were computed with unrestricted DFT. All DFT and TD-DFT calculations were performed with Gaussian 16.^74^

The photoisomerization pathway of **BDHPPy^+^** was computed with spin-flip TD-DFT (SF-TD-DFT)^75^ in the gas phase. This quantum chemical method allows the correct physical description of S_0_/S_1_ conical intersection, unlike TD-DFT.^76,77^ It uses a triplet reference state to generate the singlet ground state and electronic excited states applying one-electron spin-flip excitations. Thus, ground and excited states are described on an equal footing, in contrast to TD-DFT. SF-TD-DFT calculations were performed within the Tamm–Dancoff approximation using a spin-restricted triplet reference. For these calculations, the recommended half-and-half BHHLYP functional^78^ was used, and the *tert*-butyl groups were also replaced by hydrogen atoms for simplicity. The approximate photoisomerization pathway was constructed by computing the S_0_ and S_1_ energies using linearly interpolated structures in internal coordinates between the optimized critical structures (minima and MECI). GAMESS was used to carry out the SF-TD-DFT calculations.^79^ Note that because analytic hessian is not implemented at the SF-TD-DFT in GAMESS, the excited-state transition state was optimized with TD-DFT to evaluate the excited-state barrier on S_1_ along the **BDHPPy^+^** to **BCPDPy^+^** photoisomerization.

B3LYP/6-311G(d,p) was also used to evaluate the reaction energy for the electronic energy transfer between DHP and dioxygen. All calculations were performed with the corresponding solvent used in the experiment (i.e., water, acetonitrile,...). Open-shell singlet biradical and triplet structures were computed at the broken-symmetry UB3LYP level. Spin contamination has been taken into account using the previously mentioned procedure.^72,73^

## Supporting Information Available

Full experimental details, NMR and absorption spectra, additional electrochemical data, and Cartesian coordinates for the computed molecular structures (PDF)

## Abbreviations

DHP: dimethyldihydropyrene
CPD: cyclophanediene

## References

[ref1] Balzani V.; Ceroni P.; Juris A. Photochemistry and Photophysics: Concepts, Research, Applications; Wiley-VCH: Weinheim, 2014.

[ref2] Turro N. J.; Ramamurthy V.; Scaiano J. C. Modern Molecular Photochemistry of Organic Molecules; University Science Books: Sausalito, CA, 2010.

[ref3] Molecular Switches; Feringa B. L., Ed.; Wiley-VCH: Weinheim, Chichester, 2001.

[ref4] Zhang J.; Zhang R.; Liu K.; Li Y.; Wang X.; Xie X.; Jiao X.; Tang B. A Light-Activatable Photosensitizer for Photodynamic Therapy Based on a Diarylethene Derivative. Chem. Commun. 2021, 57, 8320–8323. 10.1039/D1CC02102H. 34319334

[ref5] Liu G.; Xu X.; Chen Y.; Wu X.; Wu H.; Liu Y. A Highly Efficient Supramolecular Photoswitch for Singlet Oxygen Generation in Water. Chem. Commun. 2016, 52, 7966–7969. 10.1039/C6CC02996E. 27251874

[ref6] Hou L.; Zhang X.; Pijper T. C.; Browne W. R.; Feringa B. L. Reversible Photochemical Control of Singlet Oxygen Generation Using Diarylethene Photochromic Switches. J. Am. Chem. Soc. 2014, 136, 910–913. 10.1021/ja4122473. 24392882

[ref7] Vickerman B. M.; Zywot E. M.; Tarrant T. K.; Lawrence D. S. Taking Phototherapeutics from Concept to Clinical Launch. Nat. Rev. Chem. 2021, 5, 816–834. 10.1038/s41570-021-00326-w. 37117665 PMC8493544

[ref8] Velema W. A.; van der Berg J. P.; Hansen M. J.; Szymanski W.; Driessen A. J. M.; Feringa B. L. Optical Control of Antibacterial Activity. Nat. Chem. 2013, 5, 924–928. 10.1038/nchem.1750. 24153369

[ref9] Broichhagen J.; Schönberger M.; Cork S. C.; Frank J. A.; Marchetti P.; Bugliani M.; Shapiro A. M. J.; Trapp S.; Rutter G. A.; Hodson D. J.; Trauner D. Optical Control of Insulin Release Using a Photoswitchable Sulfonylurea. Nat. Commun. 2014, 5, 5116 10.1038/ncomms6116. 25311795 PMC4208094

[ref10] Koçer A.; Walko M.; Feringa B. L. Synthesis and Utilization of Reversible and Irreversible Light-Activated Nanovalves Derived from the Channel Protein MscL. Nat. Protoc. 2007, 2, 1426–1437. 10.1038/nprot.2007.196. 17545979

[ref11] Presa A.; Brissos R. F.; Caballero A. B.; Borilovic I.; Korrodi-Gregório L.; Pérez-Tomás R.; Roubeau O.; Gamez P. Photoswitching the Cytotoxic Properties of Platinum(II) Compounds. Angew. Chem., Int. Ed. 2015, 54, 4561–4565. 10.1002/anie.201412157. 25689285

[ref12] Mulatihan D.; Guo T.; Zhao Y. Azobenzene Photoswitch for Isomerization-Dependent Cancer Therapy via Azo-Combretastatin A4 and Phototrexate. Photochem. Photobiol. 2020, 96, 1163–1168. 10.1111/php.13292. 32521572

[ref13] Gelder R. N. V. Regenerative and Restorative Medicine for Eye Disease. Nat. Med. 2022, 28, 1149–1156. 10.1038/s41591-022-01862-8. 35715505 PMC10718186

[ref14] Zhang Y.; Wiesholler L. M.; Rabie H.; Jiang P.; Lai J.; Hirsch T.; Lee K.-B. Remote Control of Neural Stem Cell Fate Using NIR-Responsive Photoswitching Upconversion Nanoparticle Constructs. ACS Appl. Mater. Interfaces 2020, 12, 40031–40041. 10.1021/acsami.0c10145. 32805826

[ref15] Lee I.-N.; Dobre O.; Richards D.; Ballestrem C.; Curran J. M.; Hunt J. A.; Richardson S. M.; Swift J.; Wong L. S. Photoresponsive Hydrogels with Photoswitchable Mechanical Properties Allow Time-Resolved Analysis of Cellular Responses to Matrix Stiffening. ACS Appl. Mater. Interfaces 2018, 10, 7765–7776. 10.1021/acsami.7b18302. 29430919 PMC5864053

[ref16] Roubinet B.; Bossi M. L.; Alt P.; Leutenegger M.; Shojaei H.; Schnorrenberg S.; Nizamov S.; Irie M.; Belov V. N.; Hell S. W. Carboxylated Photoswitchable Diarylethenes for Biolabeling and Super-Resolution RESOLFT Microscopy. Angew. Chem., Int. Ed. 2016, 55, 15429–15433. 10.1002/anie.201607940. PMC5132007 27767250

[ref17] Roubinet B.; Weber M.; Shojaei H.; Bates M.; Bossi M. L.; Belov V. N.; Irie M.; Hell S. W. Fluorescent Photoswitchable Diarylethenes for Biolabeling and Single-Molecule Localization Microscopies with Optical Superresolution. J. Am. Chem. Soc. 2017, 139, 6611–6620. 10.1021/jacs.7b00274. 28437075

[ref18] Uno K.; Bossi M. L.; Konen T.; Belov V. N.; Irie M.; Hell S. W. Asymmetric Diarylethenes with Oxidized 2-Alkylbenzothiophen-3-yl Units: Chemistry, Fluorescence, and Photoswitching. Adv. Opt. Mater. 2019, 7, 1801746 10.1002/adom.201801746.

[ref19] ter Schiphorst J.; Coleman S.; Stumpel J. E.; Ben Azouz A.; Diamond D.; Schenning A. P. H. J. Molecular Design of Light-Responsive Hydrogels, For in Situ Generation of Fast and Reversible Valves for Microfluidic Applications. Chem. Mater. 2015, 27, 5925–5931. 10.1021/acs.chemmater.5b01860.

[ref20] Wani O. M.; Zeng H.; Priimagi A. A Light-Driven Artificial Flytrap. Nat. Commun. 2017, 8, 15546 10.1038/ncomms15546. 28534872 PMC5457518

[ref21] Zeng H.; Wani O. M.; Wasylczyk P.; Kaczmarek R.; Priimagi A. Self-Regulating Iris Based on Light-Actuated Liquid Crystal Elastomer. Adv. Mater. 2017, 29, 1701814 10.1002/adma.201701814. 28589679

[ref22] Pärs M.; Hofmann C. C.; Willinger K.; Bauer P.; Thelakkat M.; Köhler J. An Organic Optical Transistor Operated under Ambient Conditions. Angew. Chem., Int. Ed. 2011, 50, 11405–11408. 10.1002/anie.201104193. 22113798

[ref23] Chen H.; Cheng N.; Ma W.; Li M.; Hu S.; Gu L.; Meng S.; Guo X. Design of a Photoactive Hybrid Bilayer Dielectric for Flexible Nonvolatile Organic Memory Transistors. ACS Nano 2016, 10, 436–445. 10.1021/acsnano.5b05313. 26673624

[ref24] Roldan D.; Kaliginedi V.; Cobo S.; Kolivoska V.; Bucher C.; Hong W.; Royal G.; Wandlowski T. Charge Transport in Photoswitchable Dimethyldihydropyrene-Type Single-Molecule Junctions. J. Am. Chem. Soc. 2013, 135, 5974–5977. 10.1021/ja401484j. 23574365

[ref25] Zacharias P.; Gather M. C.; Köhnen A.; Rehmann N.; Meerholz K. Photoprogrammable Organic Light-Emitting Diodes. Angew. Chem., Int. Ed. 2009, 48, 4038–4041. 10.1002/anie.200805969. 19222078

[ref26] Klajn R.; Wesson P. J.; Bishop K. J. M.; Grzybowski B. A. Writing Self-Erasing Images Using Metastable Nanoparticle “Inks”. Angew. Chem., Int. Ed. 2009, 48, 7035–7039. 10.1002/anie.200901119. 19533698

[ref27] Bléger D.; Hecht S. Visible-Light-Activated Molecular Switches. Angew. Chem., Int. Ed. 2015, 54, 11338–11349. 10.1002/anie.201500628. 26096635

[ref28] Fukaminato T.; Hirose T.; Doi T.; Hazama M.; Matsuda K.; Irie M. Molecular Design Strategy toward Diarylethenes That Photoswitch with Visible Light. J. Am. Chem. Soc. 2014, 136, 17145–17154. 10.1021/ja5090749. 25390547

[ref29] Fredrich S.; Göstl R.; Herder M.; Grubert L.; Hecht S. Switching Diarylethenes Reliably in Both Directions with Visible Light. Angew. Chem., Int. Ed. 2016, 55, 1208–1212. 10.1002/anie.201509875. 26662470

[ref30] Simke J.; Bösking T.; Ravoo B. J. Photoswitching of *Ortho* -Aminated Arylazopyrazoles with Red Light. Org. Lett. 2021, 23, 7635–7639. 10.1021/acs.orglett.1c02856. 34533955

[ref31] Hoorens M. W. H.; Medved’ M.; Laurent A. D.; Di Donato M.; Fanetti S.; Slappendel L.; Hilbers M.; Feringa B. L.; Jan Buma W.; Szymanski W. Iminothioindoxyl as a Molecular Photoswitch with 100 Nm Band Separation in the Visible Range. Nat. Commun. 2019, 10, 2390 10.1038/s41467-019-10251-8. 31160552 PMC6546742

[ref32] Xi H.; Zhang Z.; Zhang W.; Li M.; Lian C.; Luo Q.; Tian H.; Zhu W.-H. All-Visible-Light-Activated Dithienylethenes Induced by Intramolecular Proton Transfer. J. Am. Chem. Soc. 2019, 141, 18467–18474. 10.1021/jacs.9b07357. 31656065

[ref33] Hou L.; Larsson W.; Hecht S.; Andréasson J.; Albinsson B. A General Approach for All-Visible-Light Switching of Diarylethenes through Triplet Sensitization Using Semiconducting Nanocrystals. J. Mater. Chem. C 2022, 10, 15833–15842. 10.1039/D2TC03582K.

[ref34] Ishikawa M.; Ohzono T.; Yamaguchi T.; Norikane Y. Photo-Enhanced Aqueous Solubilization of an Azo-Compound. Sci. Rep. 2017, 7, 6909 10.1038/s41598-017-06947-w. 28761073 PMC5537305

[ref35] Brown C.; Rastogi S. K.; Barrett S. L.; Anderson H. E.; Twichell E.; Gralinski S.; McDonald A.; Brittain W. J. Differential Azobenzene Solubility Increases Equilibrium Cis/Trans Ratio in Water. J. Photochem. Photobiol., A 2017, 336, 140–145. 10.1016/j.jphotochem.2016.12.013.

[ref36] Gilat S. L.; Kawai S. H.; Lehn J.-M. Light-Triggered Molecular Devices: Photochemical Switching Of Optical and Electrochemical Properties in Molecular Wire Type Diarylethene Species. Chem. – Eur. J. 1995, 1, 275–284. 10.1002/chem.19950010504.

[ref37] Gorodetsky B.; Samachetty H. D.; Donkers R. L.; Workentin M. S.; Branda N. R. Reductive Electrochemical Cyclization of a Photochromic 1,2-Dithienylcyclopentene Dication. Angew. Chem., Int. Ed. 2004, 43, 2812–2815. 10.1002/anie.200353029. 15150756

[ref38] Khettabi A.; Grempka A.; Lafolet F.; Chatir E.; Leconte N.; Collomb M.; Jouvenot D.; Cobo S. Catalytic Light-Triggered Reduction Promoted by a Dithienylethene Derivative. Chem. – Eur. J. 2020, 26, 13359–13362. 10.1002/chem.201905825. 32220098

[ref39] Sheepwash M. A. L.; Mitchell R. H.; Bohne C. Mechanistic Insights into the Photochromism of *Trans*-10b,10c-Dimethyl-10b,10c-Dihydropyrene Derivatives. J. Am. Chem. Soc. 2002, 124, 4693–4700. 10.1021/ja017229e. 11971718

[ref40] Roemer M.; Gillespie A.; Jago D.; Costa-Milan D.; Alqahtani J.; Hurtado-Gallego J.; Sadeghi H.; Lambert C. J.; Spackman P. R.; Sobolev A. N.; Skelton B. W.; Grosjean A.; Walkey M.; Kampmann S.; Vezzoli A.; Simpson P. V.; Massi M.; Planje I.; Rubio-Bollinger G.; Agraït N.; Higgins S. J.; Sangtarash S.; Piggott M. J.; Nichols R. J.; Koutsantonis G. A. 2,7- and 4,9-Dialkynyldihydropyrene Molecular Switches: Syntheses, Properties, and Charge Transport in Single-Molecule Junctions. J. Am. Chem. Soc. 2022, 144, 12698–12714. 10.1021/jacs.2c02289. 35767015

[ref41] Zhang Z.; Wang W.; O’Hagan M.; Dai J.; Zhang J.; Tian H. Stepping Out of the Blue: From Visible to Near-IR Triggered Photoswitches. Angew. Chem., Int. Ed. 2022, 61, e202205758 10.1002/anie.202205758. 35524420

[ref42] Leistner A.; Pianowski Z. L. Smart Photochromic Materials Triggered with Visible Light. Eur. J. Org. Chem. 2022, 2022, e202101271 10.1002/ejoc.202101271.

[ref43] Garmshausen Y.; Klaue K.; Hecht S. Dihydropyrene as an Aromaticity Probe for Partially Quinoid Push-Pull Systems. ChemPlusChem 2017, 82, 1025–1029. 10.1002/cplu.201700068. 31961608

[ref44] Klaue K.; Garmshausen Y.; Hecht S. Taking Photochromism beyond Visible: Direct One-Photon NIR Photoswitches Operating in the Biological Window. Angew. Chem., Int. Ed. 2018, 57, 1414–1417. 10.1002/anie.201709554. 29243389

[ref45] Klaue K.; Han W.; Liesfeld P.; Berger F.; Garmshausen Y.; Hecht S. Donor-Acceptor Dihydropyrenes Switchable with Near-Infrared Light. J. Am. Chem. Soc. 2020, 142, 11857–11864. 10.1021/jacs.0c04219. 32476422

[ref46] Ayub K.; Li R.; Bohne C.; Williams R. V.; Mitchell R. H. Calculation Driven Synthesis of an Excellent Dihydropyrene Negative Photochrome and Its Photochemical Properties. J. Am. Chem. Soc. 2011, 133, 4040–4045. 10.1021/ja1100596. 21344935

[ref47] Ziani Z.; Loiseau F.; Lognon E.; Boggio-Pasqua M.; Philouze C.; Cobo S.; Royal G. Synthesis of a Negative Photochrome with High Switching Quantum Yields and Capable of Singlet-Oxygen Production and Storage. Chem. – Eur. J. 2021, 27, 16642–16653. 10.1002/chem.202103003. 34677893

[ref48] Bakkar A.; Cobo S.; Lafolet F.; Roldan D.; Saint-Aman E.; Royal G. A Redox- and Photo-Responsive Quadri-State Switch Based on Dimethyldihydropyrene-Appended Cobalt Complexes. J. Mater. Chem. C 2016, 4, 1139–1143. 10.1039/C5TC04277A.

[ref49] Bakkar A.; Cobo S.; Lafolet F.; Roldan D.; Jacquet M.; Bucher C.; Royal G.; Saint-Aman E. Dimethyldihydropyrene–Cyclophanediene Photochromic Couple Functionalized with Terpyridyl Metal Complexes as Multi-Addressable Redox- and Photo-Switches. Dalton Trans. 2016, 45, 13700–13708. 10.1039/C6DT00843G. 27264501

[ref50] Jacquet M.; Lafolet F.; Cobo S.; Loiseau F.; Bakkar A.; Boggio-Pasqua M.; Saint-Aman E.; Royal G. Efficient Photoswitch System Combining a Dimethyldihydropyrene Pyridinium Core and Ruthenium(II) Bis-Terpyridine Entities. Inorg. Chem. 2017, 56, 4357–4368. 10.1021/acs.inorgchem.6b02861. 28368594

[ref51] Roldan D.; Cobo S.; Lafolet F.; Vilà N.; Bochot C.; Bucher C.; Saint-Aman E.; Boggio-Pasqua M.; Garavelli M.; Royal G. A Multi-Addressable Switch Based on the Dimethyldihydropyrene Photochrome with Remarkable Proton-Triggered Photo-Opening Efficiency. Chem. – Eur. J. 2015, 21, 455–467. 10.1002/chem.201404858. 25358895

[ref52] Mitchell R. H.; Ward T. R. The Synthesis of Benz-, Naphth-, and Anth-Annelated Dihydropyrenes as Aids to Measuring Aromaticity by NMR. Tetrahedron 2001, 57, 3689–3695. 10.1016/S0040-4020(01)00233-2.

[ref53] Mitchell R. H.; Lai Y.-H.; Williams R. V. N-Bromosuccinimide-Dimethylformamide: A Mild, Selective Nuclear Monobromination Reagent for Reactive Aromatic Compounds. J. Org. Chem. 1979, 44, 4733–4735. 10.1021/jo00393a066.

[ref54] Mitchell R. H.; Iyer V. S.; Khalifa N.; Mahadevan R.; Venugopalan S.; Weerawarna S. A.; Zhou P. An Experimental Estimation of Aromaticity Relative to That of Benzene. The Synthesis and NMR Properties of a Series of Highly Annelated Dimethyldihydropyrenes: Bridged Benzannulenes. J. Am. Chem. Soc. 1995, 117, 1514–1532. 10.1021/ja00110a008.

[ref55] Boekelheide V.; Phillips J. B. *Trans*-15,16-Dimethyldihydropyrene: A new type of aromatic system having methyl groups within the cavity of the π-electron cloud. Proc. Natl. Acad. Sci. U. S. A. 1964, 51, 550–552. 10.1073/pnas.51.4.550. 16591157 PMC300115

[ref56] Photochromism: Molecules and Systems, Rev. ed.; Dürr H., Bouas-Laurent H., Eds.; Elsevier: Amsterdam, Boston, 2003.

[ref57] Cobo S.; Lafolet F.; Saint-Aman E.; Philouze C.; Bucher C.; Silvi S.; Credi A.; Royal G. Reactivity of a Pyridinium-Substituted Dimethyldihydropyrene Switch under Aerobic Conditions: Self-Sensitized Photo-Oxygenation and Thermal Release of Singlet Oxygen. Chem. Commun. 2015, 51, 13886–13889. 10.1039/C5CC04763C. 26214006

[ref58] Blattmann H.-R.; Meuche D.; Heilbronner E.; Molyneux R. J.; Boekelheide V. Photoisomerization of *Trans*-15,16-Dimethyldihydropyrene. J. Am. Chem. Soc. 1965, 87, 130–131. 10.1021/ja01079a031.

[ref59] Mitchell R. H.; Brkic Z.; Sauro V. A.; Berg D. J. A Photochromic, Electrochromic, Thermochromic Ru Complexed Benzannulene: An Organometallic Example of the Dimethyldihydropyrene–Metacyclophanediene Valence Isomerization. J. Am. Chem. Soc. 2003, 125, 7581–7585. 10.1021/ja034807d. 12812498

[ref60] Maafi M.; Brown R. G. The Kinetic Model for AB(1ϕ) Systems. J. Photochem. Photobiol., A 2007, 187, 319–324. 10.1016/j.jphotochem.2006.10.030.

[ref61] Maafi M.; Brown R. G. Kinetic Analysis and Elucidation Options for AB(1k,2ϕ) Systems. New Spectrokinetic Methods for Photochromes. Photochem. Photobiol. Sci. 2008, 7, 1360 10.1039/b807556e. 18958323

[ref62] Gorodetsky B.; Branda N. R. Bidirectional Ring-Opening and Ring-Closing of Cationic 1,2-Dithienylcyclopentene Molecular Switches Triggered with Light or Electricity. Adv. Funct. Mater. 2007, 17, 786–796. 10.1002/adfm.200600902.

[ref63] Kishida M.; Kusamoto T.; Nishihara H. Photoelectric Signal Conversion by Combination of Electron-Transfer Chain Catalytic Isomerization and Photoisomerization on Benzodimethyldihydropyrenes. J. Am. Chem. Soc. 2014, 136, 4809–4812. 10.1021/ja412528d. 24655047

[ref64] Muratsugu S.; Kume S.; Nishihara H. Redox-Assisted Ring Closing Reaction of the Photogenerated Cyclophanediene Form of Bis(Ferrocenyl)Dimethyldihydropyrene with Interferrocene Electronic Communication Switching. J. Am. Chem. Soc. 2008, 130, 7204–7205. 10.1021/ja8016494. 18479105

[ref65] Pedersen D.; Rosenbohm C. Dry Column Vacuum Chromatography. Synthesis 2004, 2431–2434. 10.1055/s-2001-18722.

[ref66] Becke A. D. Density-Functional Thermochemistry. III. The Role of Exact Exchange. J. Chem. Phys. 1993, 98, 5648–5652. 10.1063/1.464913.

[ref67] Chai J.-D.; Head-Gordon M. Long-Range Corrected Hybrid Density Functionals with Damped Atom–Atom Dispersion Corrections. Phys. Chem. Chem. Phys. 2008, 10, 6615–6620. 10.1039/b810189b. 18989472

[ref68] Krishnan R.; Binkley J. S.; Seeger R.; Pople J. A. Self-consistent Molecular Orbital Methods. XX. A Basis Set for Correlated Wave Functions. J. Chem. Phys. 1980, 72, 650–654. 10.1063/1.438955.

[ref69] Tomasi J.; Mennucci B.; Cammi R. Quantum Mechanical Continuum Solvation Models. Chem. Rev. 2005, 105, 2999–3094. 10.1021/cr9904009. 16092826

[ref70] Laurent A. D.; Jacquemin D. TD-DFT Benchmarks: A Review. Int. J. Quantum Chem. 2013, 113, 2019–2039. 10.1002/qua.24438.

[ref71] Martin R. L. Natural Transition Orbitals. J. Chem. Phys. 2003, 118, 4775–4777. 10.1063/1.1558471.

[ref72] Yamaguchi K.; Jensen F.; Dorigo A.; Houk K. N. A Spin Correction Procedure for Unrestricted Hartree-Fock and Møller-Plesset Wavefunctions for Singlet Diradicals and Polyradicals. Chem. Phys. Lett. 1988, 149, 537–542. 10.1016/0009-2614(88)80378-6.

[ref73] Yamanaka S.; Kawakami T.; Nagao H.; Yamaguchi K. Effective Exchange Integrals for Open-Shell Species by Density Functional Methods. Chem. Phys. Lett. 1994, 231, 25–33. 10.1016/0009-2614(94)01221-0.

[ref74] Frisch M. J.; Trucks G. W.; Schlegel H. B.; Scuseria G. E.; Robb M. A.; Cheeseman J. R.; Scalmani G.; Barone V.; Petersson G. A.; Nakatsuji H.; Li X.; Caricato M.; Marenich A. V.; Bloino J.; Janesko B. G.; Gomperts R.; Mennucci B.; Hratchian H. P.; Ortiz J. V.; Izmaylov A. F.; Sonnenberg J. L.; Williams-Young D.; Ding F.; Lipparini F.; Egidi F.; Goings J.; Peng B.; Petrone A.; Henderson T.; Ranasinghe D.; Zakrzewski V. G.; Gao J.; Rega N.; Zheng G.; Liang W.; Hada M.; Ehara M.; Toyota K.; Fukuda R.; Hasegawa J.; Ishida M.; Nakajima T.; Honda Y.; Kitao O.; Nakai H.; Vreven T.; Throssell K.; Montgomery J. A. Jr.; Peralta J. E.; Ogliaro F.; Bearpark M. J.; Heyd J. J.; Brothers E. N.; Kudin K. N.; Staroverov V. N.; Keith T. A.; Kobayashi R.; Normand J.; Raghavachari K.; Rendell A. P.; Burant J. C.; Iyengar S. S.; Tomasi J.; Cossi M.; Millam J. M.; Klene M.; Adamo C.; Cammi R.; Ochterski J. W.; Martin R. L.; Morokuma K.; Farkas O.; Foresman J. B.; Fox D. J. Gaussian 16, Rev. B.01; 2016.

[ref75] Shao Y.; Head-Gordon M.; Krylov A. I. The Spin–Flip Approach within Time-Dependent Density Functional Theory: Theory and Applications to Diradicals. J. Chem. Phys. 2003, 118, 4807–4818. 10.1063/1.1545679.

[ref76] Levine B. G.; Ko C.; Quenneville J.; Martínez T. J. Conical Intersections and Double Excitations in Time-Dependent Density Functional Theory. Mol. Phys. 2006, 104, 1039–1051. 10.1080/00268970500417762.

[ref77] Minezawa N.; Gordon M. S. Optimizing Conical Intersections by Spin–Flip Density Functional Theory: Application to Ethylene. J. Phys. Chem. A 2009, 113, 12749–12753. 10.1021/jp908032x. 19905013

[ref78] Becke A. D. A New Mixing of Hartree–Fock and Local Density-functional Theories. J. Chem. Phys. 1993, 98, 1372–1377. 10.1063/1.464304.

[ref79] Schmidt M. W.; Baldridge K. K.; Boatz J. A.; Elbert S. T.; Gordon M. S.; Jensen J. H.; Koseki S.; Matsunaga N.; Nguyen K. A.; Su S.; Windus T. L.; Dupuis M.; Montgomery J. A. General Atomic and Molecular Electronic Structure System. J. Comput. Chem. 1993, 14, 1347–1363. 10.1002/jcc.540141112.

