# Supplementary material for: All Visible Light Photoswitch Based on the Dimethyldihydropyrene Unit Operating in Aqueous Solutions with High Quantum Yields
Source: JACS Au. 2022 Dec 21;3(1):131–42. doi: 10.1021/jacsau.2c00552 (PMC9875246; doi:10.1021/jacsau.2c00552)
Supplement: Supplementary file 1 — au2c00552_si_001.pdf [file au2c00552_si_001.pdf]

Electronic Supplementary Information

All Visible-Light Photoswitch Based on the Dimethyldihydropyrene Unit Operating in Aqueous Solutions with High Quantum Yields

Zakaria Ziani,[†] Saioa Cobo,[†] Frédérique Loiseau,[†] Damien Jouvenot,[†] Elise Lognon,[‡] Martial Boggio-Pasqua,[‡,] and Guy Royal[†,*]*

[†]Univ. Grenoble Alpes, CNRS, DCM, 38000 Grenoble, France

[‡] LCPQ UMR 5626, CNRS et Université Toulouse III – Paul Sabatier, 118 route de Narbonne, 31062 Toulouse, France

Corresponding Authors

Email: guy.royal@univ-grenoble-alpes.fr

Email: martial.boggio@irsamc.ups-tlse.fr

Table of contents

1. Synthesis of $BDHPPy^+$ 2
2. Mass spectrometry data 6
3. ^{1}H and ^{13}C NMR data 9
4. Absorption Data 14
5. Electrochemical Data 15
6. Kinetic and Thermodynamic data 16
7. Quantum yields for the photoisomerization processes 19
8. Photo-kinetics experiments and data 20
9. Cartesian coordinates for optimized structures 33
10. References 46

1. Synthesis of BDHPPy+

2,7-Di-tert-butyl-trans-10b,10c-dimethyl-10b,10c-dihydropyrene (**tBuDHP**) and 4-bromo-2,7-di-tert-butyl-trans-10b,10c-dimethyl-10b-10c-dihydropyrene (**DHPBr**) were prepared following previously reported procedures.[1] **BFDHP**[2] and **BDHP**[2] and **BDHPBr**[3] were prepared using slightly modified procedures. **BDHPPy+** was prepared using the synthetic route represented in Scheme S1.

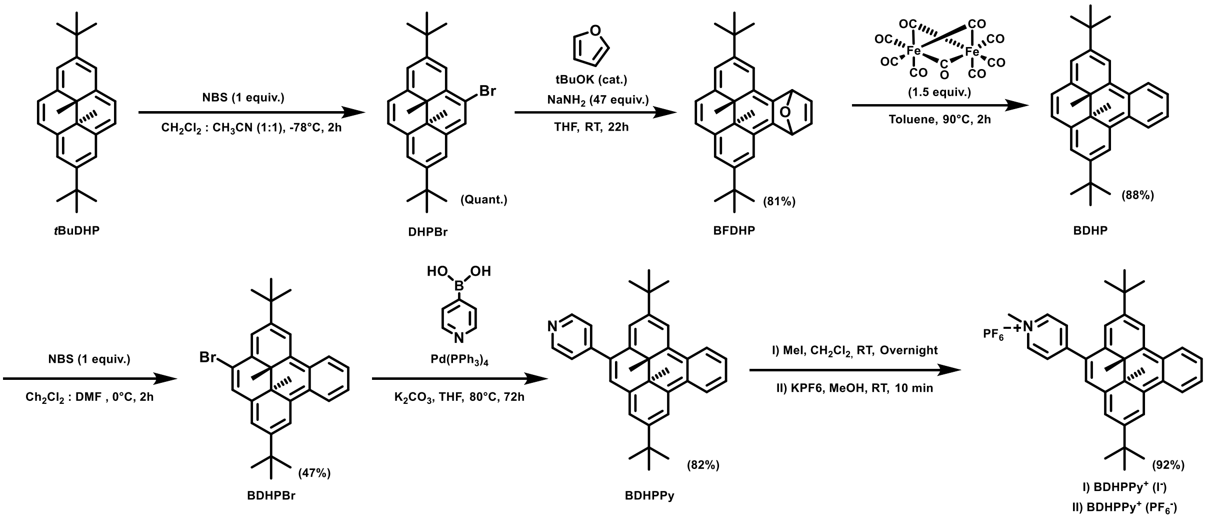

Scheme S1: Synthesis of **BDHPPy+**

2,7-di-*tert*-butyl-*trans*-10b,10c-dimethyl-10b,10c-dihydro-9,12-tetrahydro-9,12-expoxybenzo[*e*]pyren (BFDHP)[2]

Sodium amide (2.23 g, mmol) and *t*BuOK (~ 15 mg) were added under argon to a solution of **DHPBr** (500 mg, 1.18 mmol) and furan (7.2 mL) in dry THF (20 mL) in an oven dried schlenk with good magnetic stirring. After 22 hours, cyclohexane (100 mL) was added and after 10 minutes of stirring, the mixture was allowed to settle. The dark green solution was decanted carefully on celite (5 cm) and silica (2 cm). The reaction mixture solids were further rinsed with tetrahydrofuran and cyclohexane mixture (1:1) (*v:v*) until no color remains in the filtrate. The filtrate was evaporated and was purified by column chromatography over silica gel using a gradient of cyclohexane and ethyl acetate from (100:0 to 90:10) (*v:v*) as eluent. The product was obtained as dark green crystals (392.5 mg, 0.96 mmol, 81 % yield). **WARNING:** the product slowly decomposes in solid phase and rapidly in halogenated solutions.

m.p: 190-192°C
IR: 2959, 2924, 2864, 1618, 1447, 1361, 1342, 1282, 1261, 1210, 860, 834, 801, 671, 649 cm^{-1}
UV/vis (Cyclohexane) (λ_{max} [nm] ε [$M^{-1}.cm^{-1}$]): 465 (1500), 380 (8580), 361 (18600), 268 (2930)
^{1}H RMN (500 MHz, THF-*d*$_8$) δ: 8.25 – 8.15 (m, 4H, H_{ar}), 8.11 (s, 2H, H_{ar}), 7.11 (dd, J_1 = 6, J_2 = 2 Hz, 1H, H_{ar}), 6.99 (dd, J_1 = 6 Hz, J_2 = 2 Hz, 1H, , H_{ar}), 6.48 (dd, J_1 = 2 Hz, J_2 = 1 Hz, 1H, H_{ar}), 6.45 (dd, J_1 = 2 Hz, J_2 = 1 Hz, 1H, H_{ar}), 1.62 (s, 9H, H_{tBu}), 1.61 (s, 9H, H_{tBu}), -3.18 (s, 3H, H_{Me}), -3.43 (s, 3H, H_{Me}) ppm
^{13}C RMN (125 MHz, THF-*d*$_8$) δ: 145.86, 145.59, 141.10, 140.81, 138.25, 138.06, 137.94, 137.51, 128.39, 128.02, 125.30, 125.26, 122.17, 122.13, 115.81, 115.50, 81.71, 81.37, 36.45, 33.96, 33.05, 31.83, 17.06, 14.63 ppm
HRMS (m/z): DHP: Calcd. for $C_{30}H_{34}O$: 410.2604. Found: 410.2600.

2,7-di-tert-butyl-trans-10b,10c-dimethyl-10b,10c-dihydrobenzo[*e*]pyren (BDHP)[2]

A solution of **BFDHP** (219 mg, 0.53 mmol) and $Fe_2(CO)_9$ (280 mg, mmol) in dry toluene (20 mL) was stirred at 90°C in the dark, under argon atmosphere for 2h. After cooling, the mixture was filtered through a silica plug (5 cm) using toluene as eluent. The intense red solution was evaporated in the dark. The crude solid was dissolved in a minimum of cyclohexane and was filtered on a silica plug (5 cm) in order to remove the unreacted compound. The product was obtained as red crystals (185.5 mg, 0.47 mmol) in 88% yield.

m.p: 171-173°C
IR: 2961, 2923, 2864, 1618, 1603, 1474, 1459, 1445, 1361, 1335, 1264, 883, 872, 752, cm^{-1}
UV/vis (Cyclohexane) (λ_{max} [nm] ε [$M^{-1}.cm^{-1}$]) / closed isomer: 504 (7000), 388 (35000), 369 (26400), 338 (27800), 321 (25500), 308 (24900)
UV/vis (Cyclohexane) (λ_{max} [nm] ε [$M^{-1}.cm^{-1}$]) / open isomer: 346 (9260), 267 (29300)
^{1}H RMN (500 MHz, $CDCl_3$) δ: 8.77 (m, 2H, H_{ar}), 8.28 (d, *J* = 1 Hz, 2H, H_{ar}), 7.61 (m, 2H, H_{ar}), 7.35 (d, *J* = 1 Hz, 2H, H_{ar}), 7.13 (s, 2H, H_{ar}), 1.49 (s, 18H, H_{tBu}), -1.58 (s, 6H, H_{Me}) ppm
^{13}C RMN (125 MHz, $CDCl_3$) δ: 144.55, 138.53, 134.88, 129.45, 125.80, 124.63, 121.13, 119.87, 117.12, 35.56, 35.39, 30.84, 17.55 ppm
HRMS (m/z): DHP: Calcd. for $C_{30}H_{34}$: 394.2655. Found: 394.2653.

4-bromo-2,7-di-*tert*-butyl-*trans*-10b,10c-dimethyl-10b,10c-dihydrobenzo[*e*]pyren (BDHPBr)[3]

A solution of NBS (55 mg, 0.31 mmol) in dry DMF (12 mL) was added slowly in the dark to a solution of 2,7-di-*tert*-butyl-*trans*-10b,10c-dimethyl-10b,10c-dihydrobenzo[*e*]pyrene **BDHP** (122 mg, 0.31 mmol) in dry CH_2Cl_2 (40 mL) under argon at 0 °C. The cooling bath was then removed and the reaction mixture was allowed to stir at room temperature for 1 h. The reaction mixture was poured into cyclohexane (100 mL) and the organic solution was washed with water (3 x 30 mL), brine (30 mL), dried over $MgSO_4$ and evaporated under reduced pressure. The red residue was dissolved in cyclohexane and was filtered through a plug of silica gel. Evaporation of the filtrate gave the desired compound (69 mg, 0.23 mmol) as a red powder in 74% yield. If needed, the sample can be purified further by recrystallization in cyclohexane.

m.p: 178-179°C
IR: 2954, 2910, 2861, 1604, 1552, 1464, 1444, 1357, 1335, 1255, 1237, 1201, 887, 750 cm^{-1}
UV/vis (Cyclohexane) (λ_{max} [nm] ε [$M^{-1}.cm^{-1}$]) / closed isomer: 538 (3500), 505 (4180), 472 (3090), 392 (24300), 373 (18000), 339 (17800), 324 (15700), 309 (14200), 293 (10700), 280 (7380), 268 (8580)
UV/vis (Cyclohexane) (λ_{max} [nm] ε [$M^{-1}.cm^{-1}$]) / open isomer: 335 (3790), 321 (3980), 290 (9900), 279 (10300), 261 (17800), 255 (21800)
^{1}H RMN (500 MHz, $CDCl_3$) δ: 8.76 – 8.70 (m, 2H, H_{ar}), 8.27 (d, *J* = 1 Hz, 1H, H_{ar}), 8.25 (d, *J* = 1 Hz, 1H, H_{ar}), 7.67 – 7.62 (m, 3H), 7.28 (s, 1H, H_{ar}), 7.25 (s, 1H, H_{ar}), 1.52 (s, 9H, H_{tBu}), 1.49 (s, 9H, H_{tBu}), -1.41 (s, 3H, H_{Me}), -1.42 (s, 3H, H_{Me}) ppm
^{13}C RMN (125 MHz, $CDCl_3$) δ: 148.5, 146.8, 146.3, 139.6, 135.5, 135.3, 133.1, 129.2, 126.2, 124.7, 124.5, 118.7, 118.5, 117.3, 117.1, 114.1, 38.8, 35.8, 35.7, 35.5, 30.5, 17.8, 17.0 ppm
HRMS (m/z): DHP: Calcd. for $C_{30}H_{33}Br$: 472.1760. Found: 472.1752.

4-(4-pyridyl)-2,7-di-*tert*-butyl-*trans*-10b,10c-dimethyl-10b,10c-dihydrobenzo[e]pyren (BDHPPy)

A round bottom flask was filled under an argon atmosphere with 4-bromo-2,7-di-*tert*-butyl-*trans*-10b,10c-dimethyl-10b,10c-dihydrobenzo[*e*]pyrene **BDHPBr** (100 mg, 0.21 mmol), 4-pyridinylboronic acid (34 mg, 0.28 mmol), potassium carbonate (139.6 mg, 1.01 mmol), tetrakistriphenylphosphine palladium(0) (24.3 mg, 0.021 mmol). Freshly distilled tetrahydrofuran (10 mL) and distilled water (3 mL) were then added. The mixture was heated at 80 °C for 72 h. After cooling

down the mixture to room temperature, the mixture was filtered over celite and the resulting organic layer was diluted in dichloromethane (30 mL). The solution was washed with water (3 x 30 mL), brine (30 mL), dried over anhydrous $MgSO_4$ and evaporated under reduce pressure. The crude product was purified by column chromatography on silica gel using a gradient of cyclohexane and ethyl acetate (100:0 to 90:10 ; *v:v*) as eluent. The desired product was obtained (81.7 mg, 0.17 mmol) as a pink solid (yield: 82%).

m.p: 182-184°C
IR: 2963, 2928, 2865, 1593, 1472, 1408, 1366, 1345, 1258, 1240, 886, 817, 758 cm^{-1}
UV/vis (Cyclohexane) (λ_{max} [nm] ε [$M^{-1}.cm^{-1}$]) / closed isomer: 543 (3520), 507 (4130), 475 (3030), 394 (26600), 377 (20120), 341 (18050), 324 (16550), 309 (16160), 269 (20490)
UV/vis (Cyclohexane) (λ_{max} [nm] ε [$M^{-1}.cm^{-1}$]) / open isomer: 359 (3340), 314 (5570), 266 (25670)
^{1}H RMN (500 MHz, $CDCl_3$) δ / closed isomer : 8.80 – 8.75 (m, 2H, H_{ar}), 8.75 – 8.70 (m, 2H, H_{ar}), 8.33 (dd, J_1 = 7 Hz, J_2 = 1 Hz, 2H, H_{ar}), 7.66 – 7.63 (m, 2H, H_{ar}), 7.55 (d, J = 1 Hz, 1H, H_{ar}), 7.53 – 7.49 (m, 2H, H_{ar}), 7.40 – 7.38 (m, 1H, H_{ar}), 7.17 (d, J = 1 Hz, 1H, H_{ar}), 1.53 (s, 9H, H_{tBu}), 1.44 (s, 9H, H_{tBu}), -1.37 (s, 3H, H_{Me}), -1.38 (s, 3H, H_{Me}) ppm
^{1}H RMN (500 MHz, $CDCl_3$) δ: / open isomer : 8.63 – 8.60 (m, 2H, H_{ar}), 7.71 – 7.67 (m, 2H, H_{ar}), 7.54 – 7.52 (m, 2H, H_{ar}), 7.45 – 7.40 (m, 2H, H_{ar}), 6.94 – 6.91 (m, 2H, H_{ar}), 6.91 – 6.89 (m, 2H, H_{ar}), 6.64 (d, J = 2 Hz, 1H, H_{ar}), 1.32 (s, 3H, H_{Me}), 1.29 (s, 3H, H_{Me}), 1.27 (s, 9H, H_{tBu}), 1.21 (s, 9H, H_{tBu}) ppm
^{13}C RMN (125 MHz, $CDCl_3$) δ: 150.21, 149.86, 146.57, 145.81, 138.73, 136.05, 135.37, 134.94, 130.17, 129.71, 129.66, 126.27, 126.21, 125.13, 124.86, 124.65, 123.00, 119.98, 117.42, 117.16, 116.81, 36.36, 35.95, 35.64, 35.62, 30.82, 30.80, 18.03, 17.82 ppm
HRMS (m/z): Calcd. for $C_{35}H_{37}N$ 471.2999. Found. 471.2993.

4-(2,7-di-tert-butyl-trans-10b,10c-dimethyl-10b,10c-dihydrobenzo[e]pyren-4-yl)-1-methylpyridin-1-ium (BDHPPy$^+$)

A solution of 4-(4-pyridyl)-2,7-di-tert-butyl-trans-10b,10c-dimethyl-10b,10c-dihydrobenzo[*e*]pyrene **BDHPPy** (20 mg, 0.042 mmol) in CH_2Cl_2 (10 mL) was prepared under inert atmosphere. Methyl iodide (1 mL, excess) was added to the previous solution and the resulting mixture was allowed to stir overnight under inert atmosphere. The solvent was then evaporated under reduced pressure and the residue was dissolved in MeOH (1 mL). The product was subjected to an anion exchange by addition of a saturated aqueous solution of KPF_6 (2 mL). The solid was filtered under vacuum and washed several times with water and diethyl ether. The solid was then dried under reduced pressure to give the targeted product as a brown powder (24 mg, 0.039 mmol) in 92% yield. If needed, the compound can be further purified either by column chromatography over silica gel using a mixture of acetonitrile:MeOH:H_2O:NaBr (4:1:1:1) (v:v:v:v) followed by a an anion exchange using a saturated aqueous solution of KPF_6 (2 mL) in methanol (1 mL) or by a recrystallization in methanol.

m.p: 250-252°C
IR: 2995, 2956, 2902, 2862, 1635, 1558, 1514, 1462, 1368, 1345, 1258, 873, 842, 752 cm^{-1}
UV/vis (Acetonitrile) (λ_{max} [nm] ε [$M^{-1}.cm^{-1}$]) / closed isomer: 661 (800), 556 (3000), 405 (14000), 335 (11000), 307 (13000)
UV/vis (Acetonitrile) (λ_{max} [nm] ε [$M^{-1}.cm^{-1}$]) / open isomer: 455 (3000), 392 (6000), 321 (7000), 284 (13000)
UV/vis (Water) (λ_{max} [nm] ε [$M^{-1}.cm^{-1}$]) / closed isomer: 660 (1100), 557 (2600), 403 (8100), 336 (5400), 309 (6100), 259 (4400)
UV/vis (Water) (λ_{max} [nm] ε [$M^{-1}.cm^{-1}$]) / open isomer: 456 (1800), 392 (4400), 327 (4500), 286 (7100)
^{1}H RMN (500 MHz, CD_3CN) δ / closed isomer : 8.95 – 8.91 (m, 2H, H_{ar}), 8.62 (d, J = 6 Hz, 2H, H_{ar}), 8.49 (s, 1H, H_{ar}), 8.44 (s, 1H, H_{ar}), 8.18 (d, J = 6 Hz, 2H, H_{ar}), 7.75 – 7.69 (m, 2H, H_{ar}), 7.65 (s, 1H, H_{ar}), 7.52 (s,

1H, H_{ar}), 7.31 (s, 1H, H_{ar}), 4.32 (s, 3H, H_{NMe}), 1.51 (s, 9H, H_{tBu}), 1.47 (s, 9H, H_{tBu}), -1.39 (s, 3H, H_{Me}), -1.44 (s, 3H, H_{Me}) ppm

^{1}H RMN (500 MHz, CD_3CN) δ/ open isomer: 8.47 (d, *J* = 7 Hz, 2H, H_{ar}), 8.10 (d, *J* = 7 Hz, 2H, H_{ar}), 7.81 – 7.68 (m, 2H, H_{ar}), 7.48 – 7.45 (m, 2H, H_{ar}), 7.42 (s, 1H, H_{ar}), 7.09 (d, *J* = 2 Hz, 1H, H_{ar}), 7.02 (d, *J* = 2 Hz, 1H, H_{ar}), 7.01 (d, *J* = 2 Hz, 1H, H_{ar}), 6.69 (d, *J* = 2 Hz, 1H, H_{ar}), 4.25 (s, 3H, H_{NMe}), 1.28 (s, 3H, H_{Me}), 1.27 (s, 9H, H_{tBu}), 1.22 (s, 9H, H_{tBu}), 1.21 (s, 3H, H_{Me}) ppm

^{13}C RMN (125 MHz, CD_3CN)) δ/ closed isomer: 159.05, 151.36, 147.86, 145.53, 139.92, 139.31, 138.61, 136.06, 130.40, 129.99, 129.22, 128.08, 127.69, 127.37, 126.16, 125.76, 121.93, 120.68, 118.97, 115.92, 48.41, 37.74, 36.89, 36.27, 36.11, 30.65, 30.57, 18.27, 18.21 ppm

^{13}C RMN (125 MHz, CD_3CN)) δ/ open isomer: 159.71, 153.13, 151.64, 145.55, 144.34, 144.29, 142.66, 141.66, 141.33, 140.57, 140.56, 140.40, 137.22, 137.11, 131.44, 130.94, 129.88, 129.82, 129.76, 125.74, 125.35, 48.25, 35.00, 34.86, 31.42, 31.34, 19.83, 18.85 ppm

HRMS (m/z): Calcd. for $C_{36}H_{40}N^+$ 486.3155. Found. 486.3142.

2. Mass spectrometry data

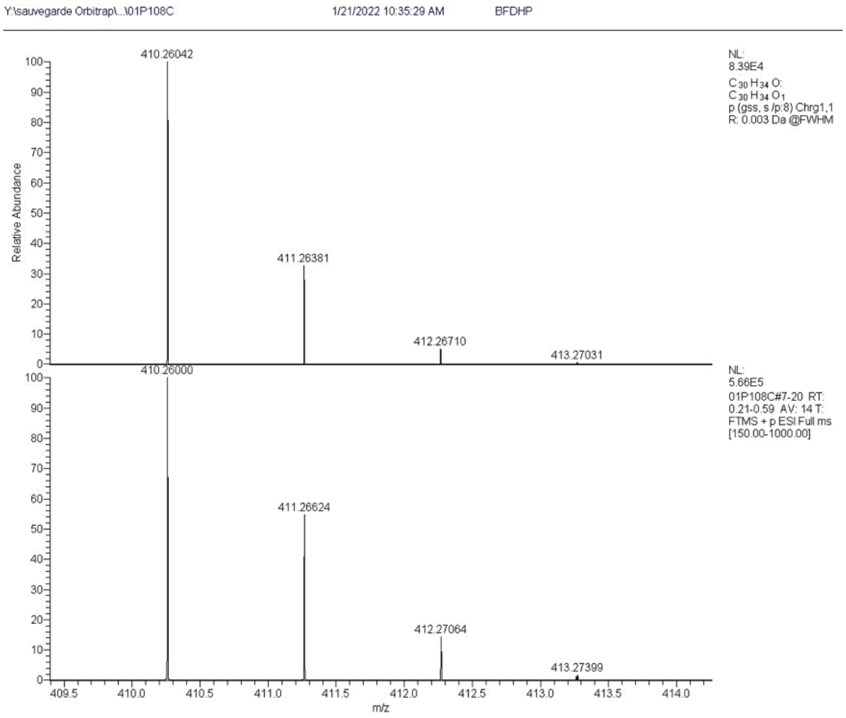

Figure S1: High-resolution mass spectrum of **BFDHP** (top: experimental, bottom: calculated).

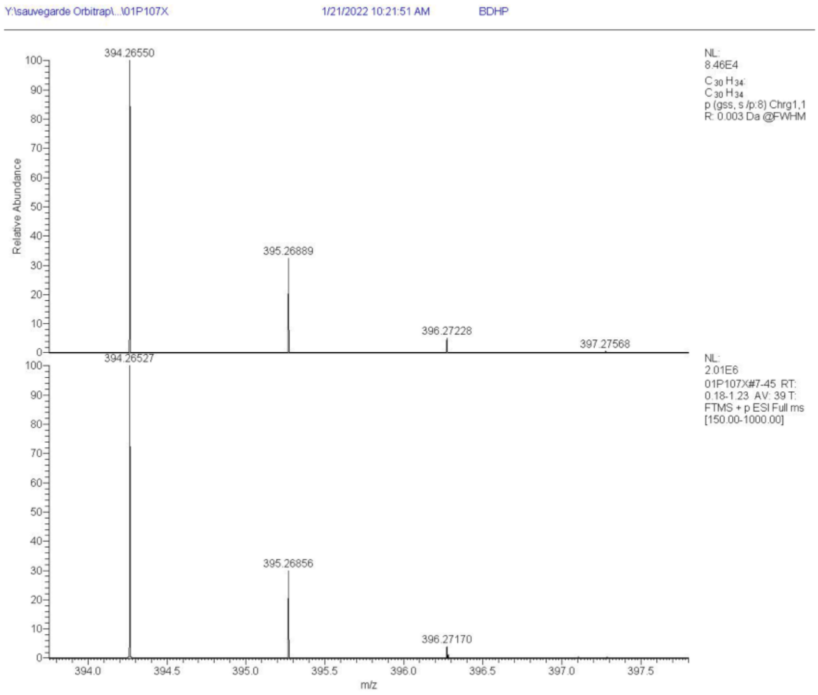

Figure S2: High-resolution mass spectrum of **BDHP** (top: experimental, bottom: calculated).

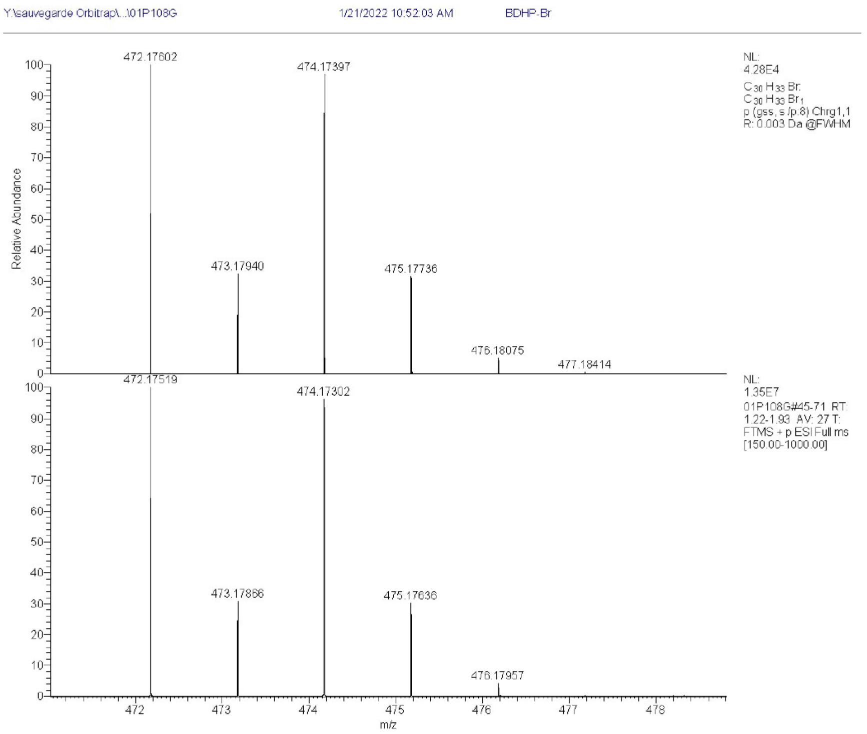

Figure S3: High-resolution mass spectrum of **BDHPBr** (top: experimental, bottom: calculated).

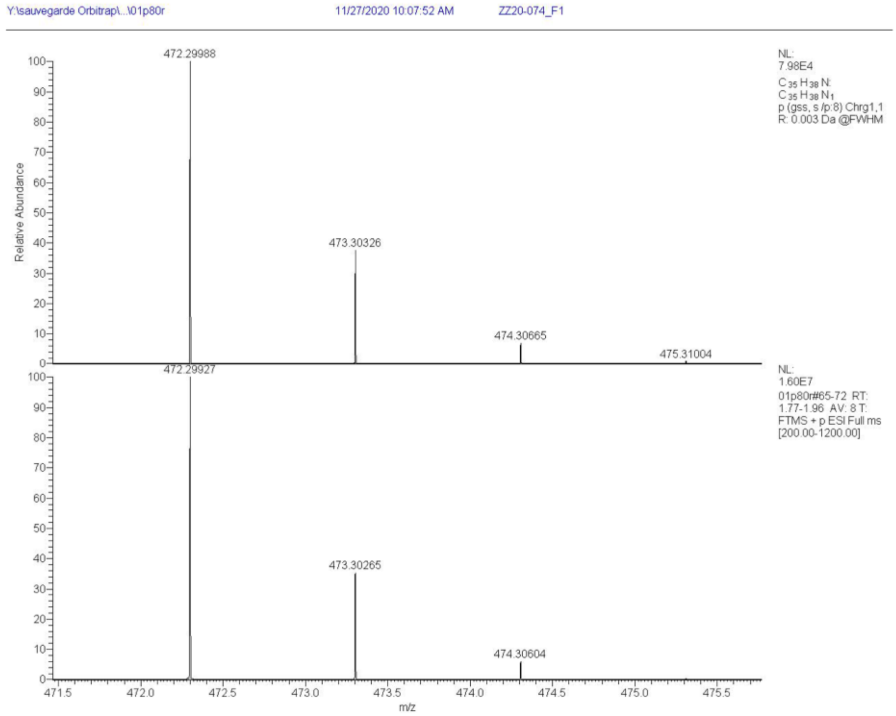

Figure S4: High-resolution mass spectrum of **BDHPPy** (top: experimental, bottom: calculated).

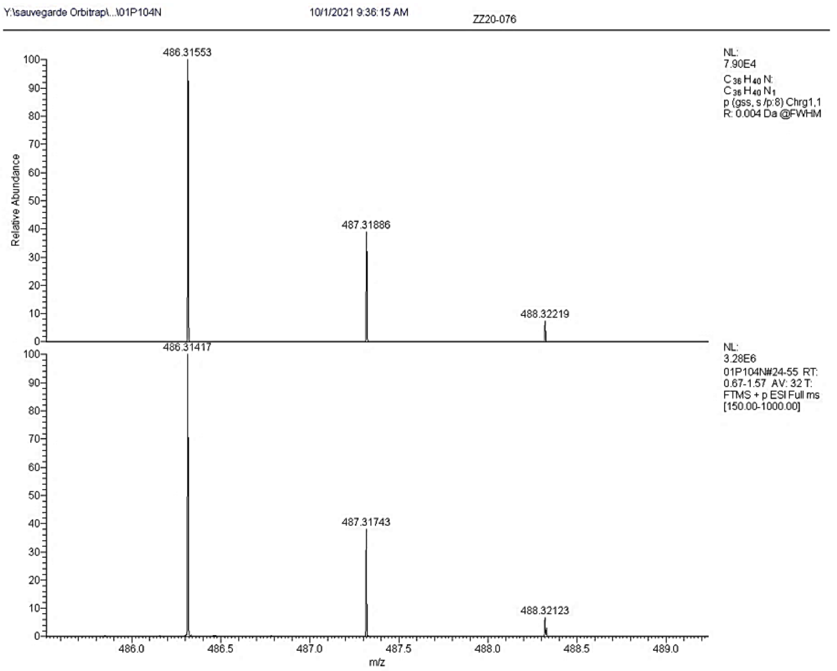

Figure S5: High-resolution mass spectrum of **BDHPPy⁺** (top: experimental, bottom: calculated).

3. ^{1}H and ^{13}C NMR data

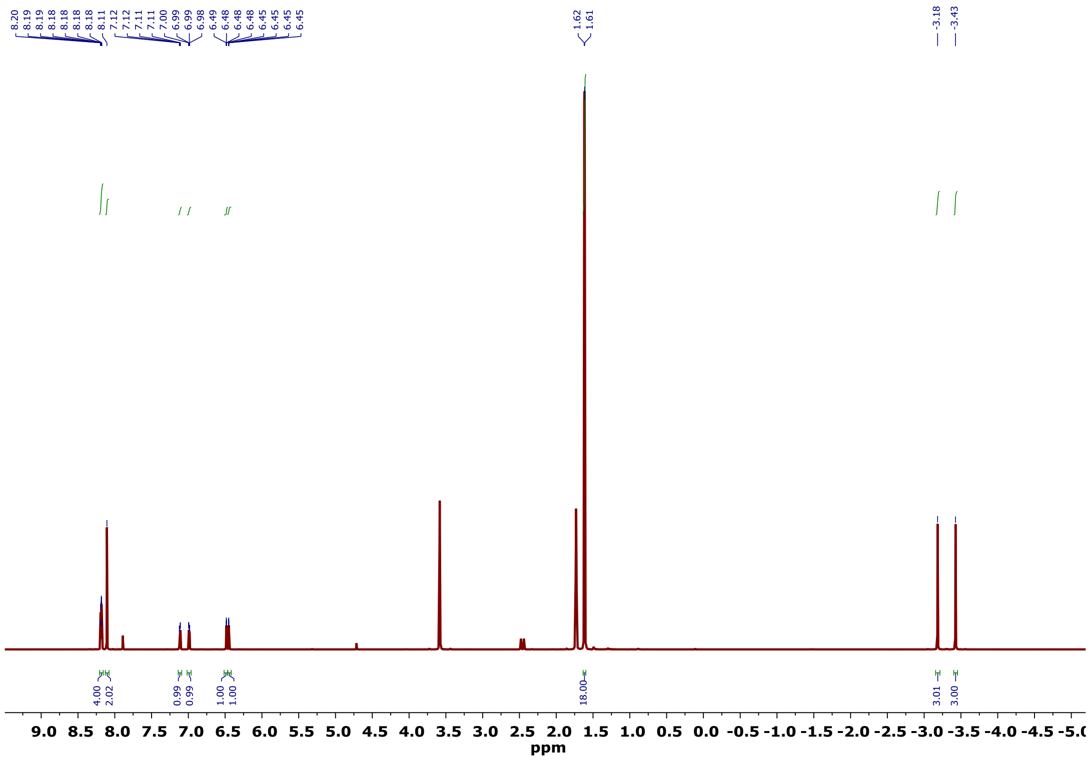

Figure S6: ^{1}H NMR spectrum of **BFDHP** in THF-d_8.

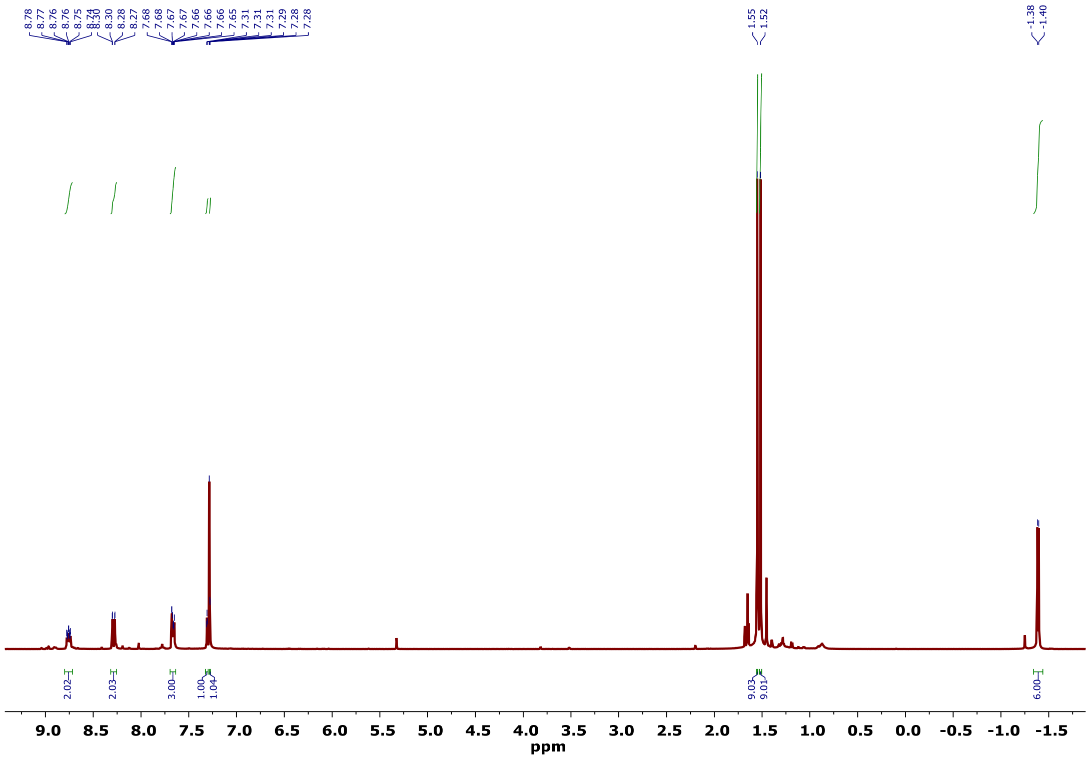

Figure S7: ^{1}H NMR spectrum of **BDHPBr** in $CDCl_3$.

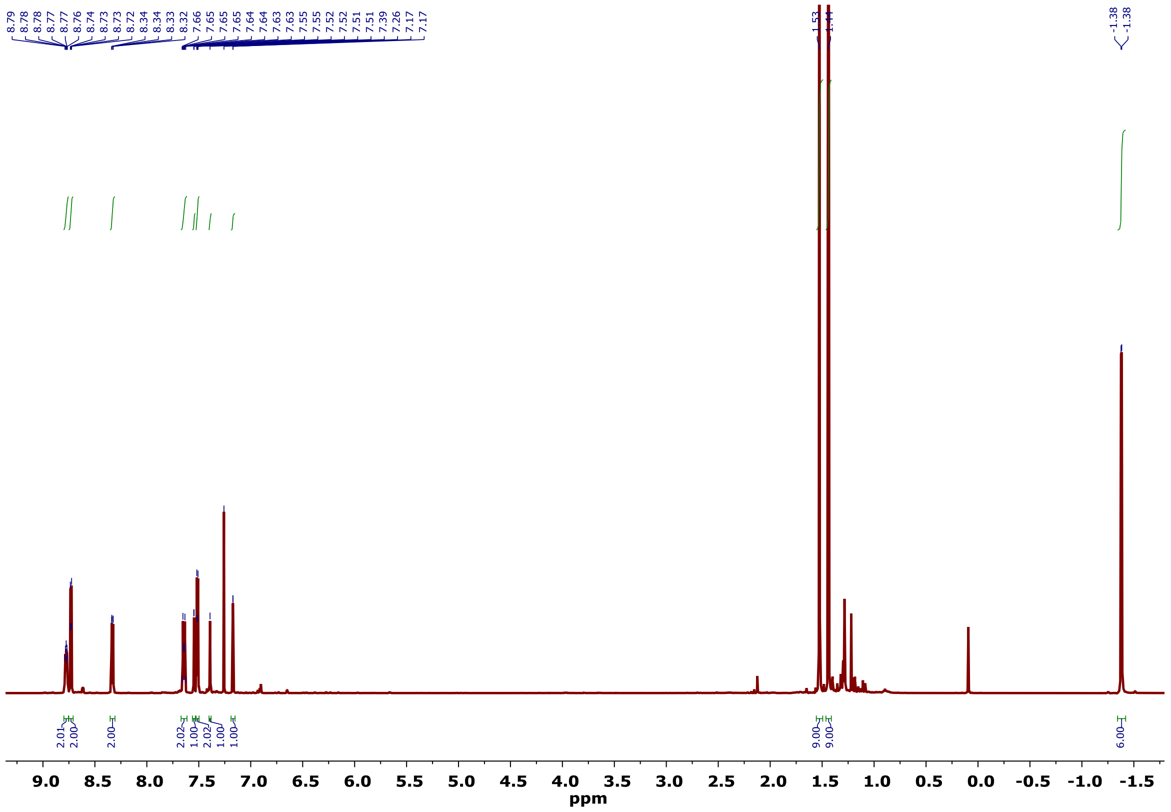

Figure S8: ^{1}H NMR spectrum of **BDHPPy** in $CDCl_3$.

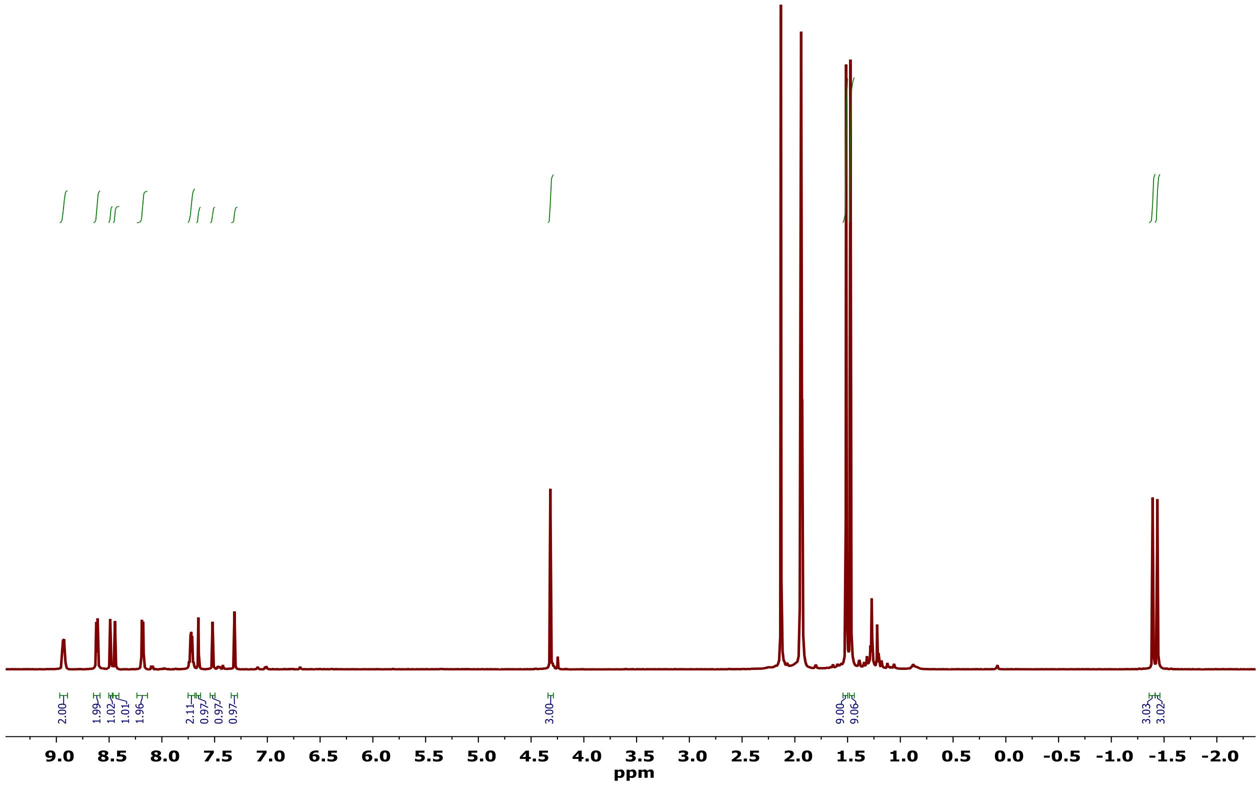

Figure S9: ^{1}H NMR spectrum of **BDHPPy⁺** in CD_3CN.

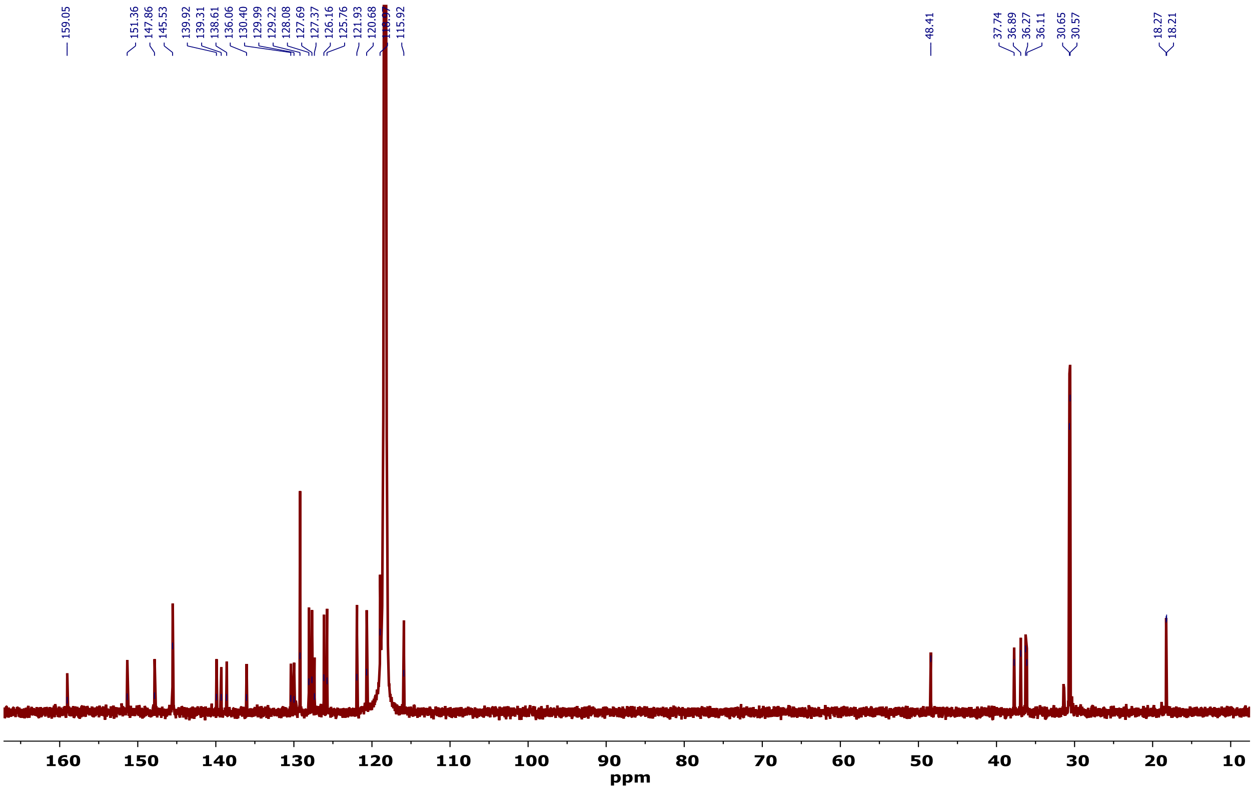

Figure S10: ^{13}C NMR spectrum of **BDHPPy^{+}** in CD_3CN.

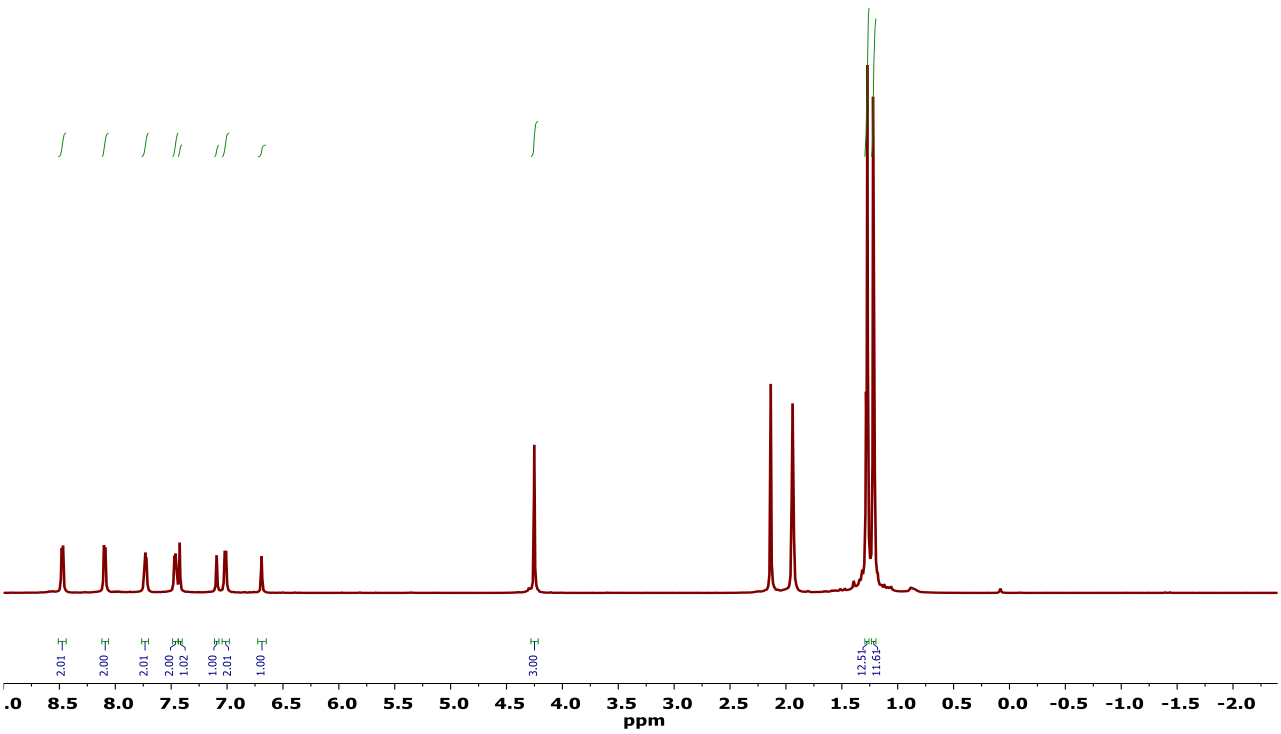

Figure S11: ^{1}H NMR spectrum of **BCPDPy^{+}** in CD_3CN.

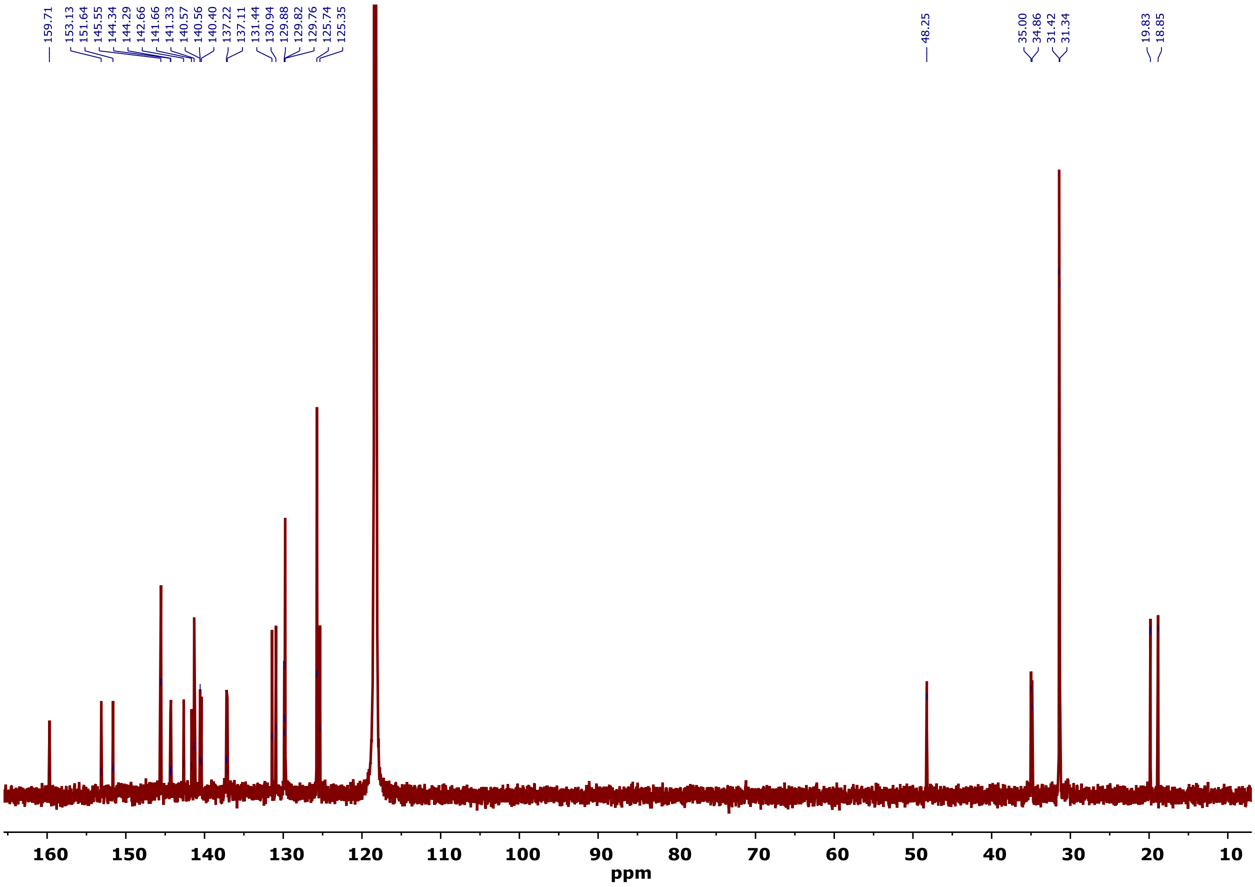

Figure S12: ^{13}C NMR spectrum of **BCPDPy⁺** in CD_3CN.

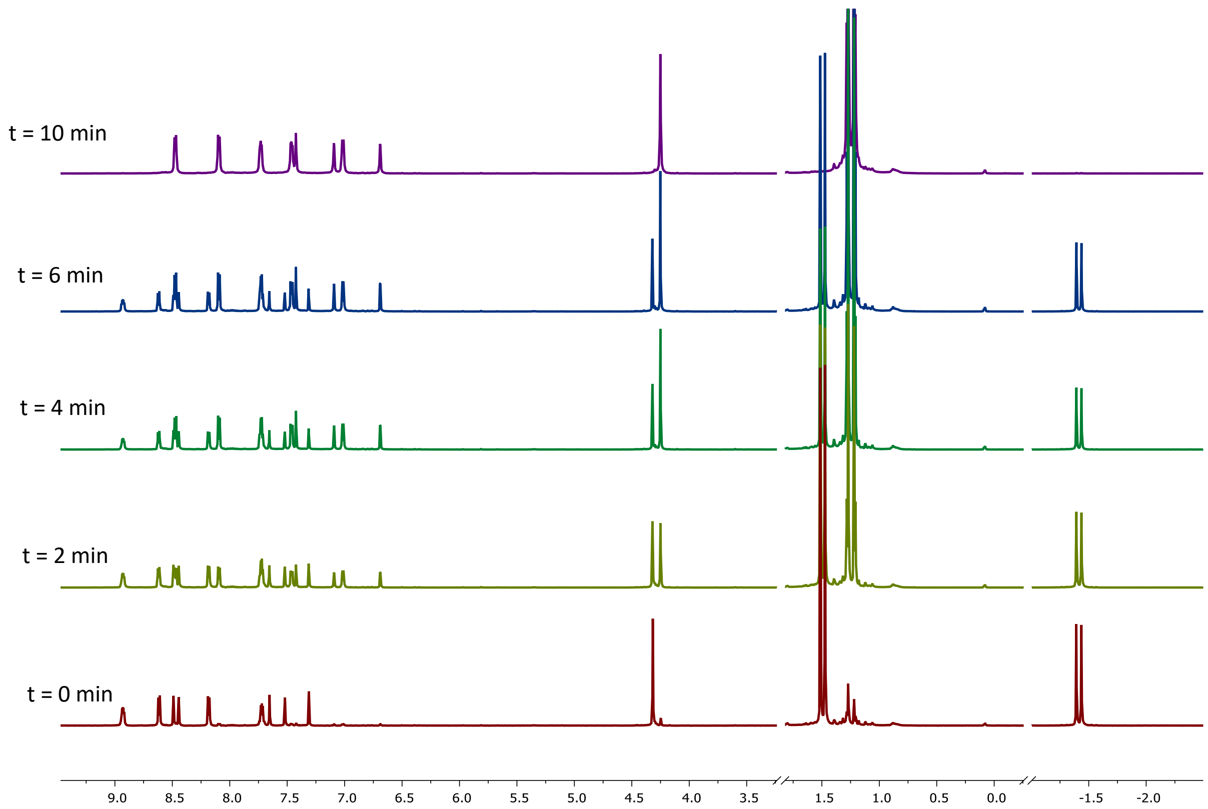

Figure S13: Evolution of the ^{1}H NMR signals of **BDHPPy^{+}, PF$_6^{-}$** during irradiation (λ = 660 nm, T = 277K, Conc. = 2 mM in CD_3CN). The peaks of the initial **BDHPPy^{+}** are progressively replaced by the signals of the open **BCPDPy^{+}** isomer.

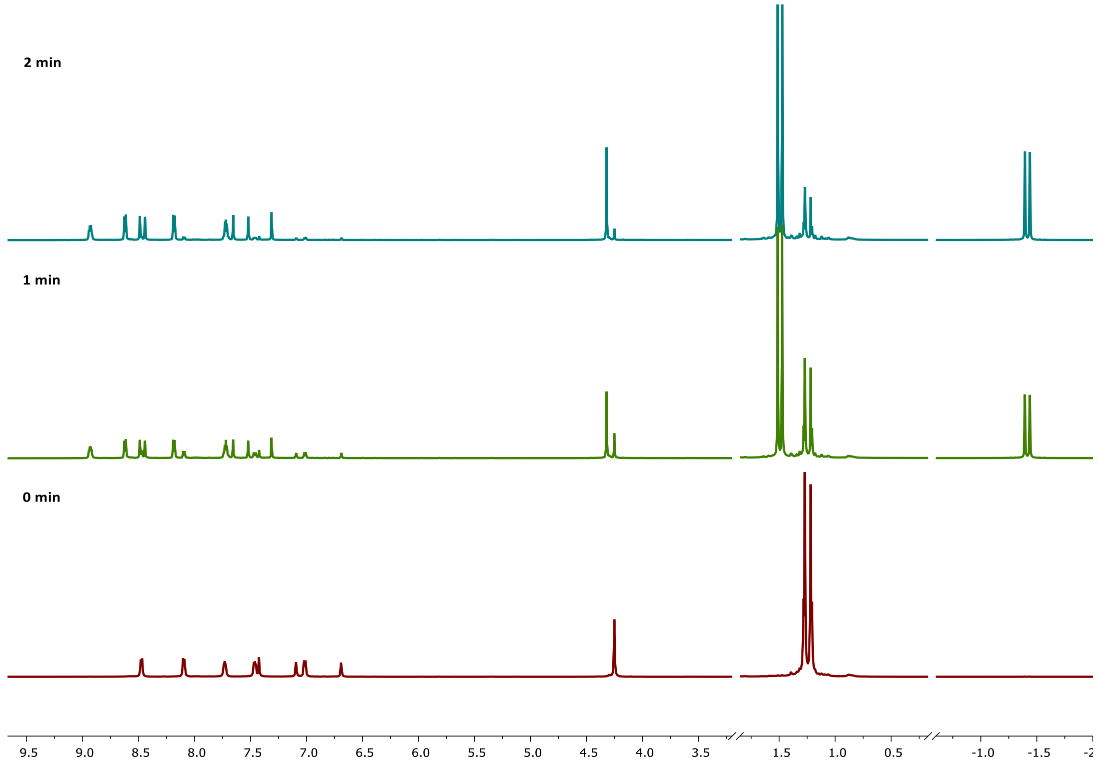

Figure S14: Evolution of the ^{1}H NMR signals of **BCPDPy^{+}, PF$_6^{-}$** during irradiation (λ = 470 nm, T = 277K, Conc. = 2 mM in CD_3CN). The peaks of the initial **BCPDPy^{+}** are progressively replaced by the signals of the closed **BDHPPy^{+}** isomer.

4. Absorption Data

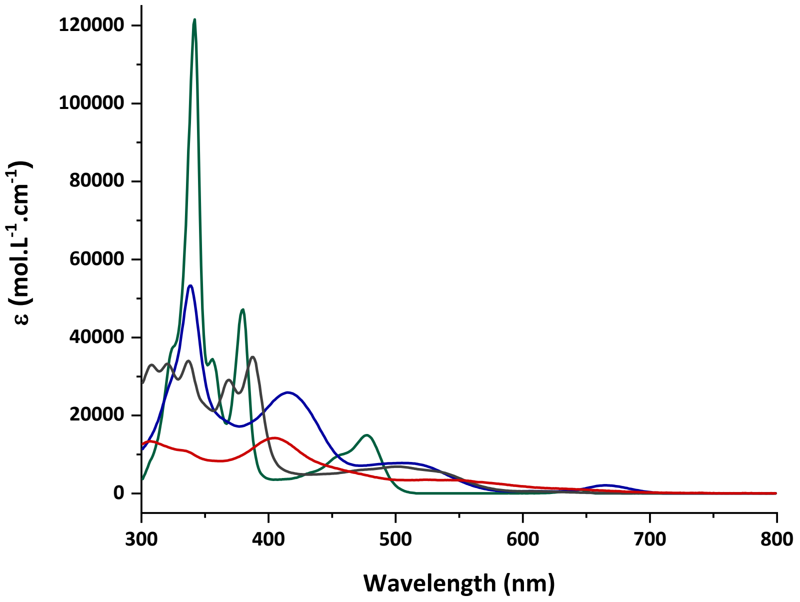

Figure S15: UV/vis spectra between 300-800 nm of ***t*BuDHP** (green line), **BDHP** (grey line), **DHPPy$^+$** (blue line) and **BDHPPy$^+$** (red line). Solvents used: cyclohexane for ***t*BuDHP** and **BDHP**; acetonitrile for **DHPPy$^+$** and **BDHPPy$^+$**.

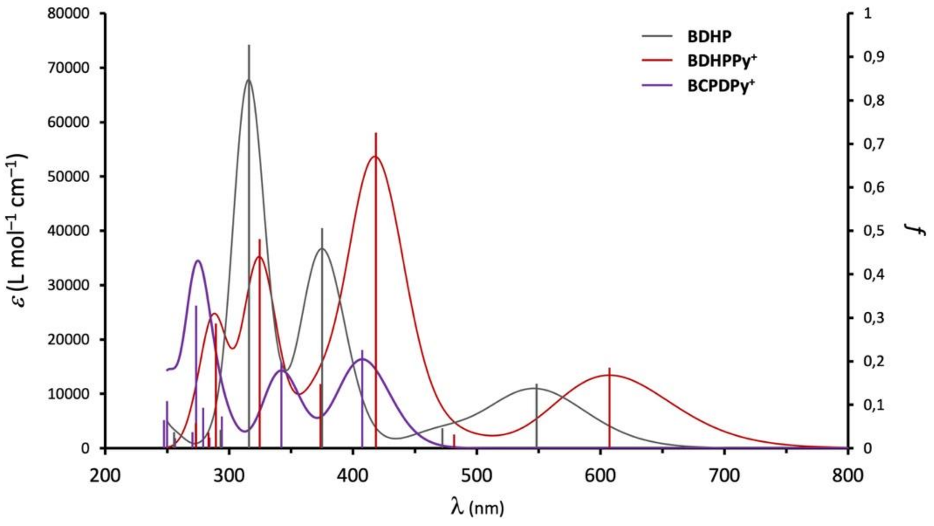

Figure S16: Simulated UV/vis absorption spectra between 250 nm-800 nm of **BDHP** (grey line), **BDHPPy$^+$** (red line) and **BCPDPy$^+$** (purple line) at the TD-ωB97X-D/6-311G(d,p) level. Solvents used: cyclohexane for **BDHP**; acetonitrile for **BDHPPy$^+$** and **BCPDPy$^+$**. The oscillator strength (*f*) for each vertical transition is indicated by a vertical line. Convoluted spectra are obtained with a phenomenological Gaussian broadening characterized by a half-width at half-height of 1500 cm^{-1}.

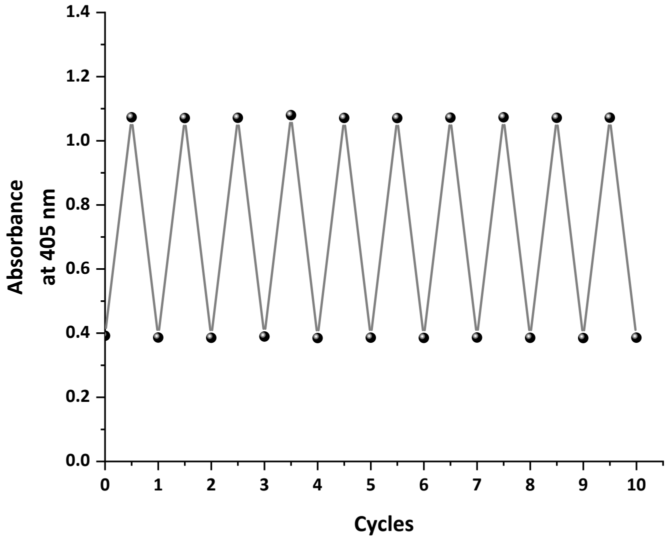

Figure S17: Study of the fatigue resistance of the **BDHPPy⁺/BCPDPy⁺** photochromic couple (PF_6^- salts) in CH_3CN. The graphical show changes in absorption for each cycle upon alternative illuminations at 470 nm and 660 nm.

5. Electrochemical Data

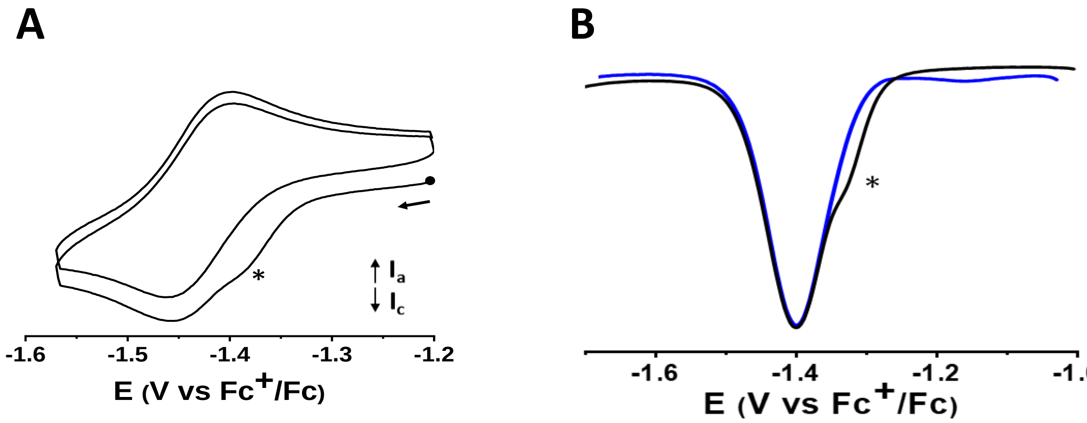

Figure S18: (A) 2 consecutive cyclic voltammograms of **BCPDPy**$^{+\cdot}$ (scan rate: 100 mv.s^{-1}). **(B)** Differential Pulse Voltammetry of **BCPDPy**$^{+\cdot}$ (black line) and **BDHPPy**$^{+\cdot}$ (blue line). [Conc.] ~ 1 mmol in 0.1M $TBAPF_6/CH_3CN$. * indicates the reduction signal centered on the pyridinium unit in the open form; the signal at lower potential corresponds to the reduction of the closed isomer.

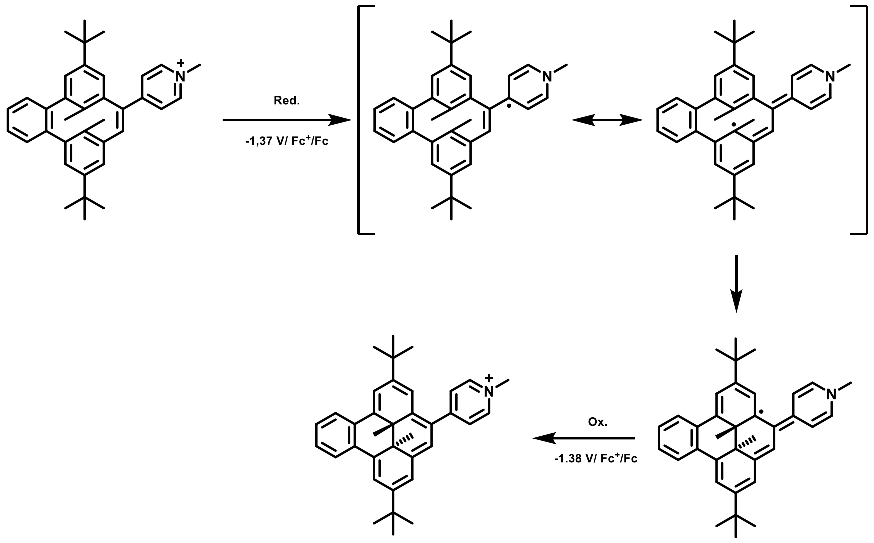

Figure S19: Proposed mechanism for the electron induced closing of **BCPDPy**$^+$ into **BDHPPy**$^+$.

6. Kinetic and Thermodynamic data

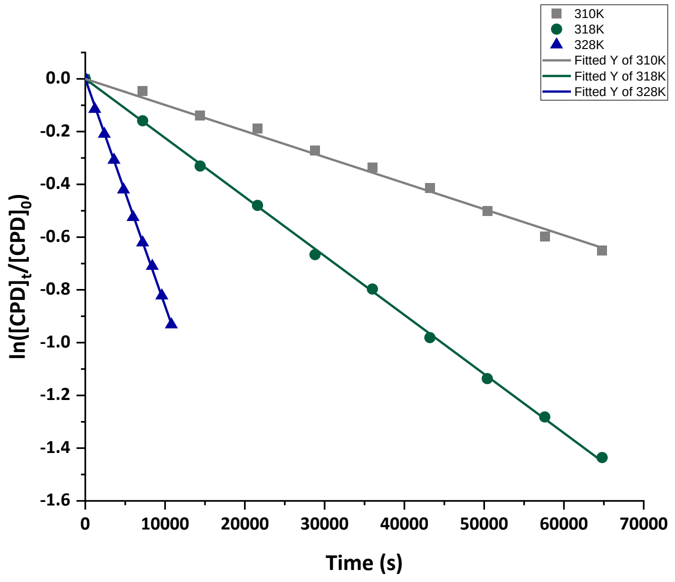

Figure S20: Relative pseudo-first order rate constants for the thermal closing (measured at 405 nm) of **BCPDPy**$^+$, PF_6^- in degassed CH_3CN followed by absorption spectroscopy.

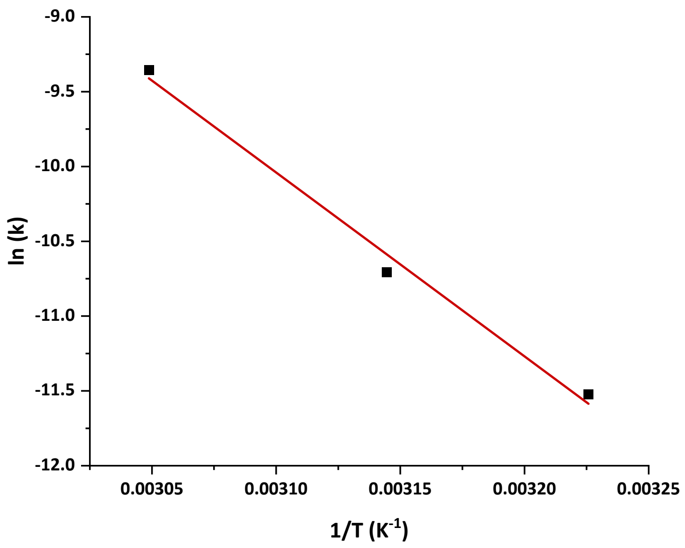

Figure S21: Arrhenius plot corresponding to the thermal closing of **BCPDPy$^+$**, PF_6^- in degassed CH_3CN.

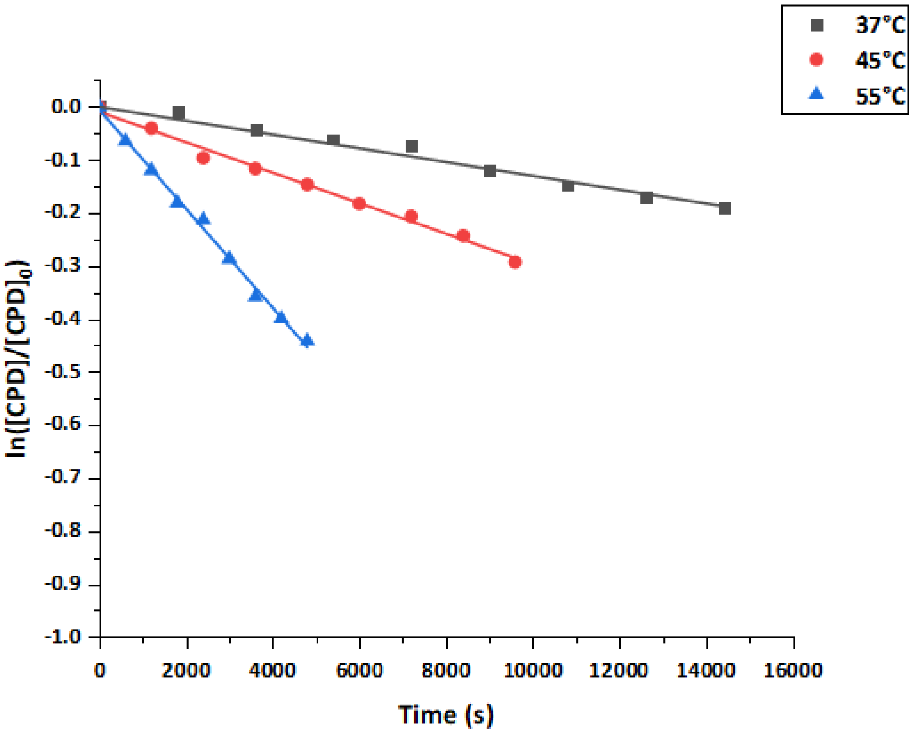

Figure S22: Relative pseudo-first order rate constants for the thermal closing (measured at 405 nm) of **BCPDPy$^+$, I^-** in degassed H_2O followed by absorption spectroscopy.

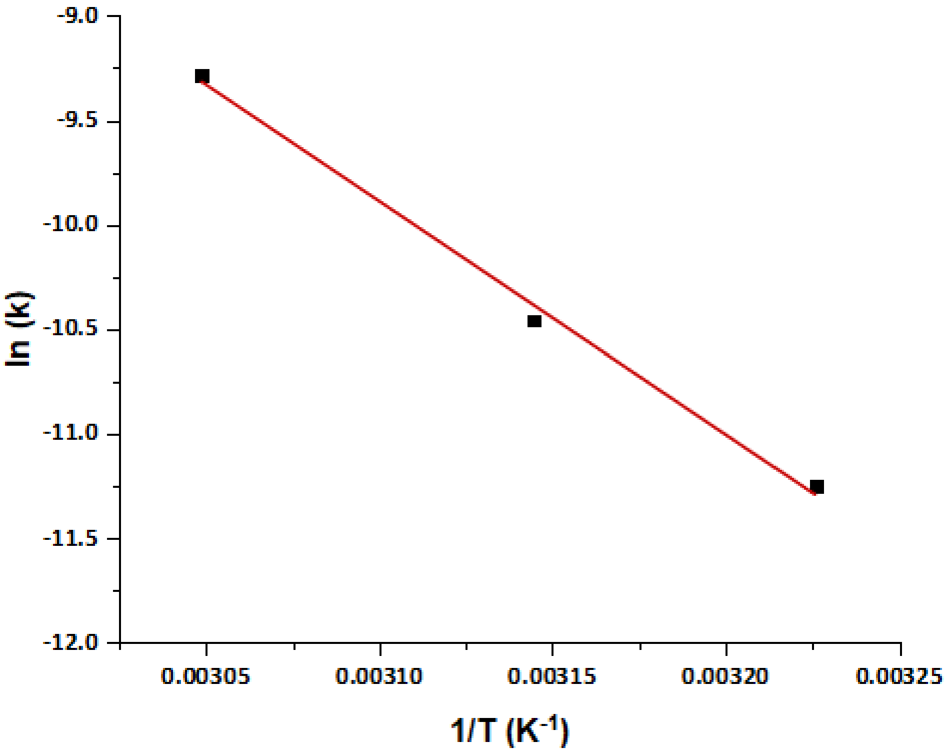

Figure S23: Arrhenius plot corresponding to the thermal closing of of **BCPDPy$^+$, I$^-$** in degassed H_2O followed by absorption spectroscopy.

7. Quantum yields for the photoisomerization processes

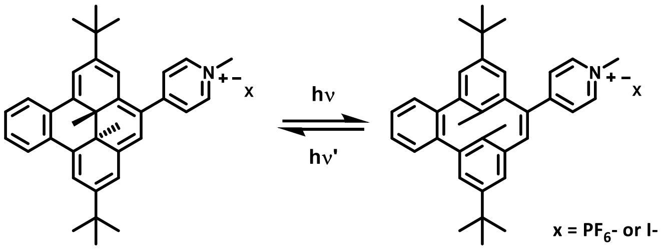

Table S1. Quantum yields for the photo-opening (**$\phi_{c\text{-}o}$**) and photo-closing (**$\phi_{o\text{-}c}$**) reactions for the **BDHPPy$^+$/BCPDPy$^+$** couple (as I^- salt) at different wavelengths, corresponding extinction coefficients ε of the open and closed isomers and PSS. Solvent: H_2O

Wavelength (nm)	ε BDHPPy+ ($M^{-1}.cm^{-1}$)	$\phi_{c\text{-}o}$ (%)	ε BCPDPy+ ($M^{-1}.cm^{-1}$)	$\phi_{o\text{-}c}$ (%)	PSS (%) (BDHPPy$^+$:BCPDPy$^+$)
365	4878	-	3448	≈ 100[a]	97:3
415	7381	≈ 100[a]	3522	≈ 100[a]	92:8
450	4158	≈ 100[a]	1972	≈ 100[a]	95:5
470	3488	≈ 100[a]	1356	≈ 100[a]	95:5
505	2698	39.9 ± 0.2	607	≈ 100[a]	56:44
530	2708	17.5 ± 0.4	0	0	100:0
590	1972	11.0 ± 0.4	0	0	100:0
617	1452	13.5 ± 0.6	0	0	100:0
660	887	15.4 ± 0.1	0	0	100:0
680	689	14.5 ± 0.5	0	0	100:0

[a] High measurement uncertainty due to an overly efficient process.

Table S2. Quantum yields for the photo-opening (**$\phi_{c\text{-}o}$**) and photo-closing (**$\phi_{o\text{-}c}$**) reactions of the **BDHPPy$^+$/BCPDPy$^+$** couple (as PF_6^- salt) at different wavelengths, corresponding extinction coefficients ε of the open and closed isomers and PSS. Solvent: CH_3CN

Wavelength (nm)	ε BDHPPy+ ($M^{-1}.cm^{-1}$)	$\phi_{c\text{-}o}$ (%)	ε BCPDPy+ ($M^{-1}.cm^{-1}$)	$\phi_{o\text{-}c}$ (%)	PSS (%) (BDHPPy$^+$:BCPDPy$^+$)
365	-	-	4254	92 ± 3	95:5
415	12968	19.1 ± 0.4	4442	86 ± 5	82:18
450	6700	42 ± 2	2950	94 ± 4	95:5
470	5020	25 ± 5	2079	81 ± 3	95:5
505	3411	28 ± 2	721	29.7 ± 0.3	74:26
530	3092	13.0 ± 0.3	0	0	100:0
590	2258	13.2 ± 0.2	0	0	100:0
617	1399	15.8 ± 0.2	0	0	100:0
660	806	16.5 ± 0.2	0	0	100:0
680	460	20.0 ± 0.5	0	0	100:0

8. Photo-kinetics experiments and data

A) Experimental study of the photo-opening process in acetonitrile

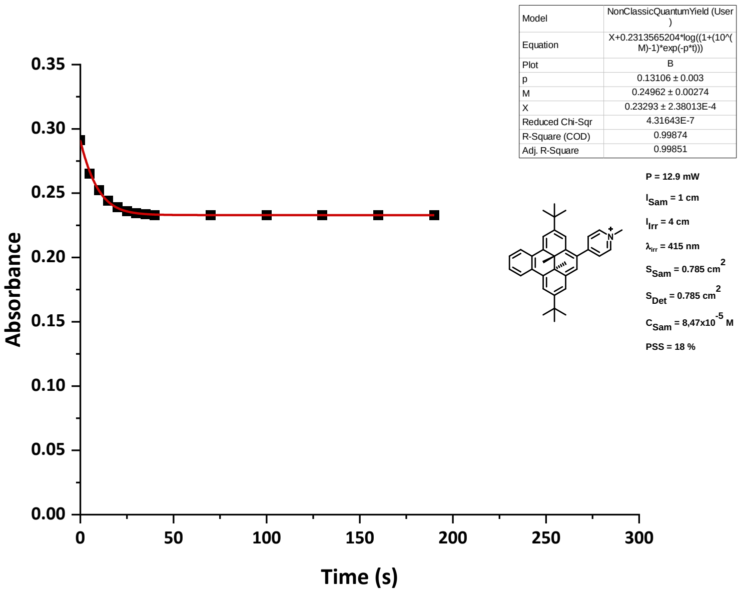

Figure S24: Evolution of the absorbance at λ = 415 nm during irradiation at λ_{ex} = 415 nm of **BDHPPy$^+$, PF6$^-$** in acetonitrile as function of time and corresponding fit using Maafi and Brown photo-kinetic model.

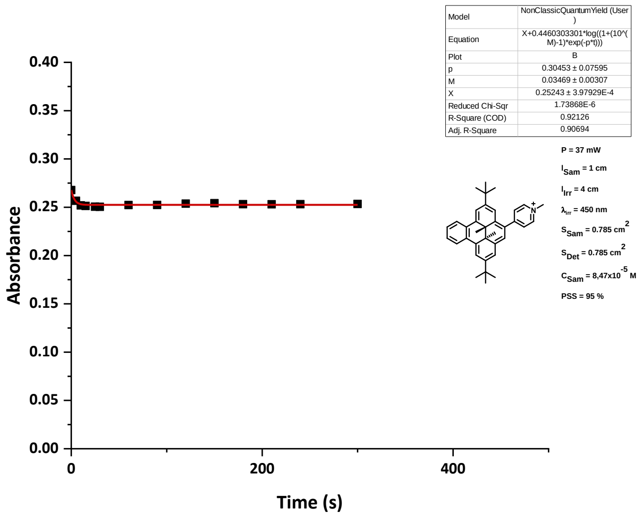

Figure S25: Evolution of the absorbance at λ = 450 nm during an irradiation at λ_{ex} = 450 nm of **BDHPPy$^+$, PF6$^-$** in acetonitrile as function of time and its fit using Maafi and Brown photo-kinetic model.

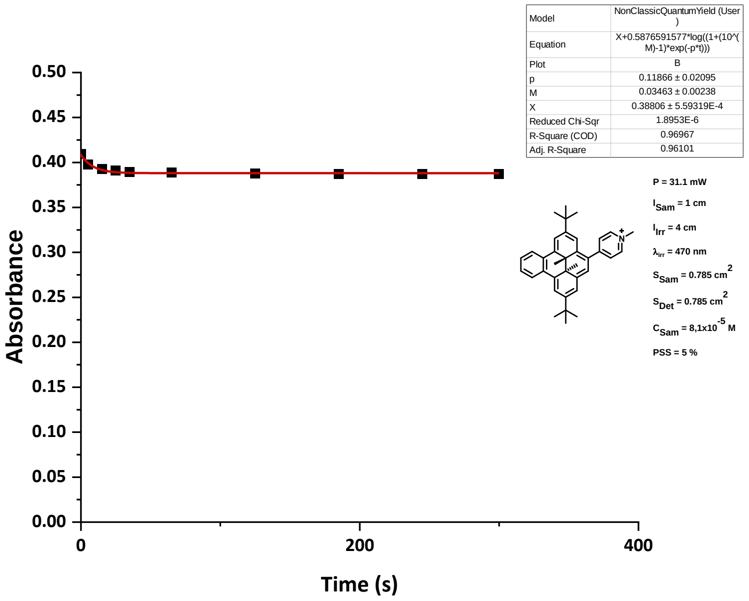

Figure S26: Evolution of the absorbance at λ = 470 nm during an irradiation at λ_{ex} = 470 nm of **BDHPPy$^+$, PF6$^-$** in acetonitrile as function of time and its fit using Maafi and Brown photo-kinetic model.

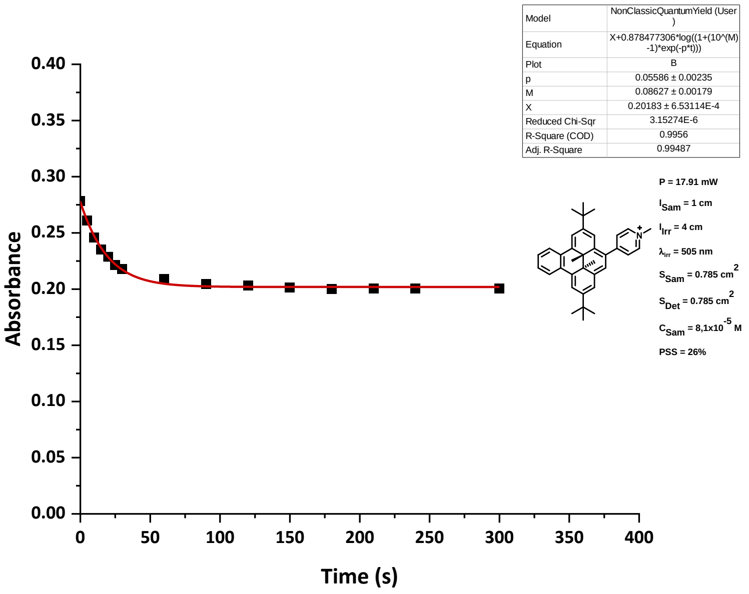

Figure S27: Evolution of the absorbance at λ = 505 nm during an irradiation at λ_{ex} = 505 nm of **BDHPPy$^+$, PF6$^-$** in acetonitrile in function of time and its fit using Maafi and Brown photo-kinetic model.

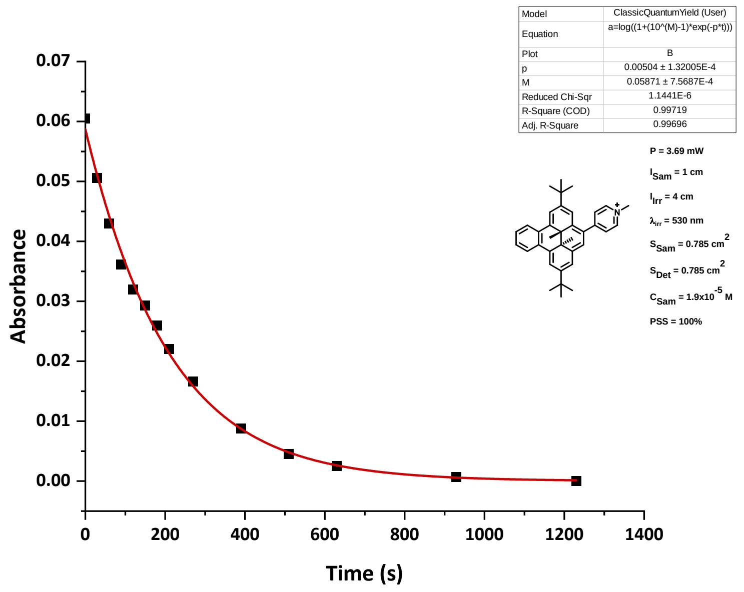

Figure S28: Evolution of the absorbance at λ = 530 nm during an irradiation at λ_{ex} = 530 nm of **BDHPPy$^+$, PF6$^-$** in acetonitrile as function of time and its fit using Maafi and Brown photo-kinetic model.

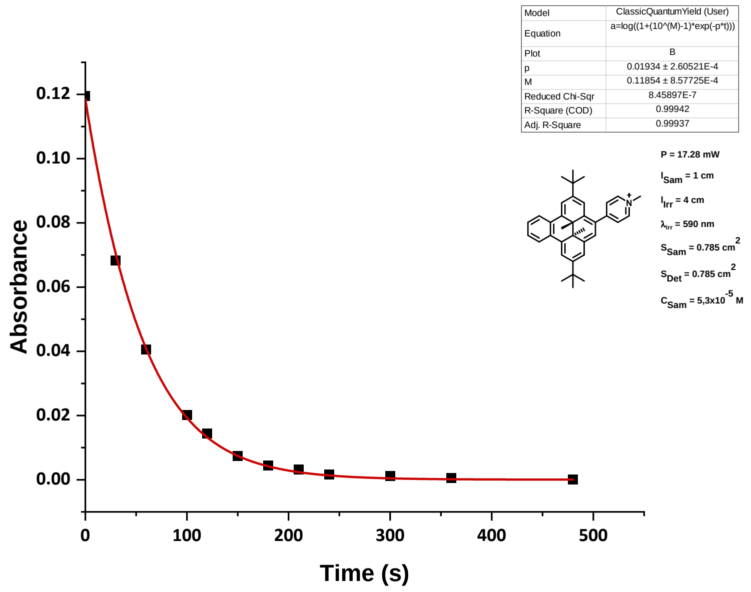

Figure S29: Evolution of the absorbance at λ = 590 nm during an irradiation at λ_{ex} = 590 nm of **BDHPPy$^+$, PF6$^-$** in acetonitrile as function of time and its fit using Maafi and Brown photo-kinetic model.

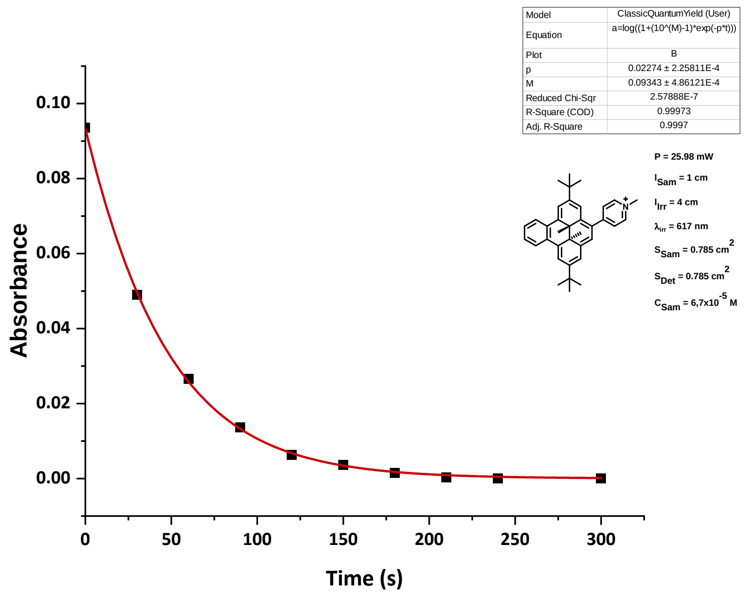

Figure S30: Evolution of the absorbance at λ = 617 nm during an irradiation at λ_{ex} = 617 nm of **BDHPPy+, PF6-** in acetonitrile in function of time and its fit using Maafi and Brown photo-kinetic model.

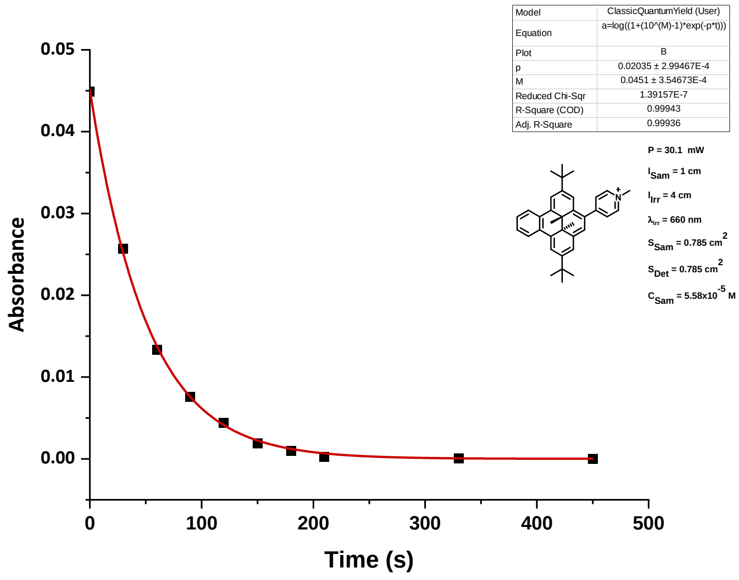

Figure S31: Evolution of the absorbance at λ = 660 nm during an irradiation at λ_{ex} = 660 nm of **BDHPPy+, PF6-** in acetonitrile as function of time and its fit using Maafi and Brown photo-kinetic model.

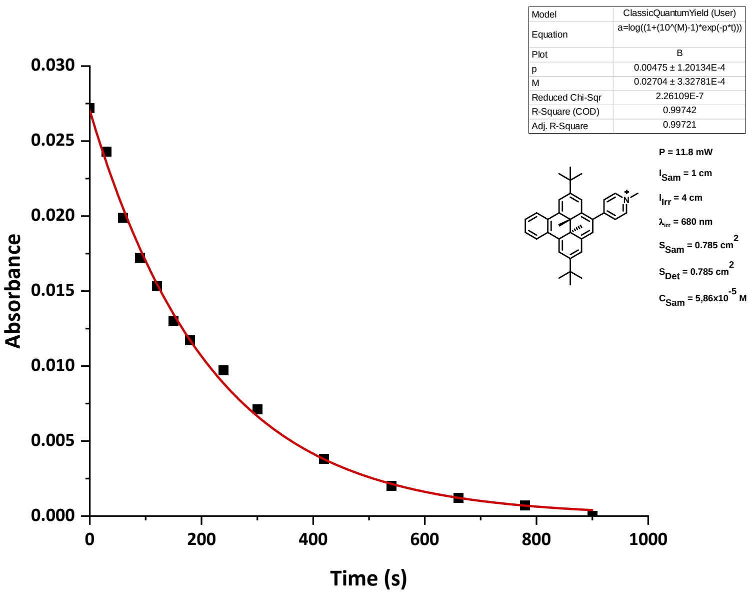

Figure S32: Evolution of the absorbance at λ = 660 nm during an irradiation at λ_{ex} = 660 nm of **BDHPPy$^+$, PF$_6^-$** in acetonitrile as function of time and its fit using Maafi and Brown photo-kinetic model.

B) Experimental study of the photo-closing process in acetonitrile

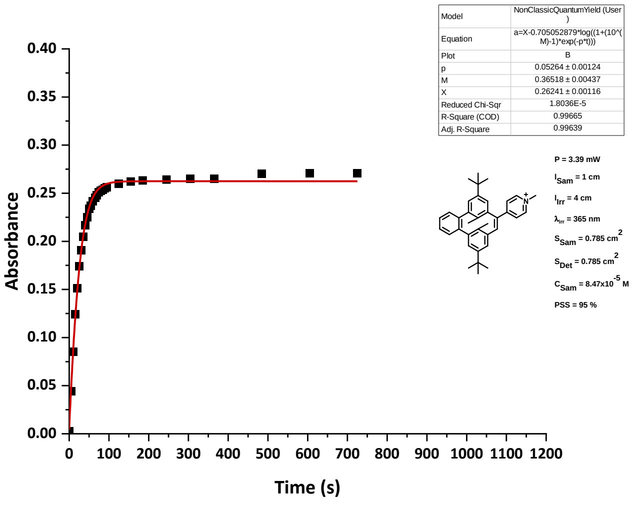

Figure S33: Evolution of the absorbance at λ = 660 nm during an irradiation at λ_{ex} = 365 nm of **BCPDPy$^+$, PF$_6^-$** in acetonitrile as function of time and its fit using Maafi and Brown photo-kinetic model.

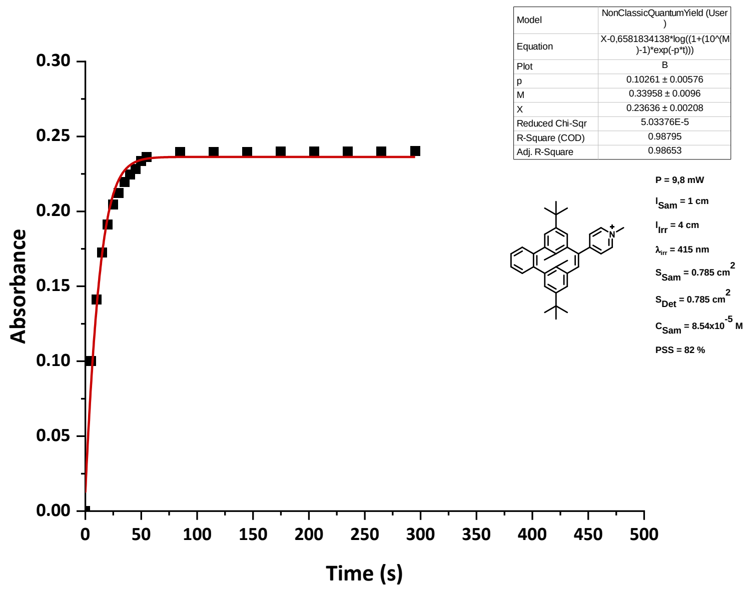

Figure S34: Evolution of the absorbance at λ = 660 nm during an irradiation at λ_{ex} = 415 nm of **BCPDPy$^+$, PF$_6^-$** in acetonitrile as function of time and its fit using Maafi and Brown photo-kinetic model.

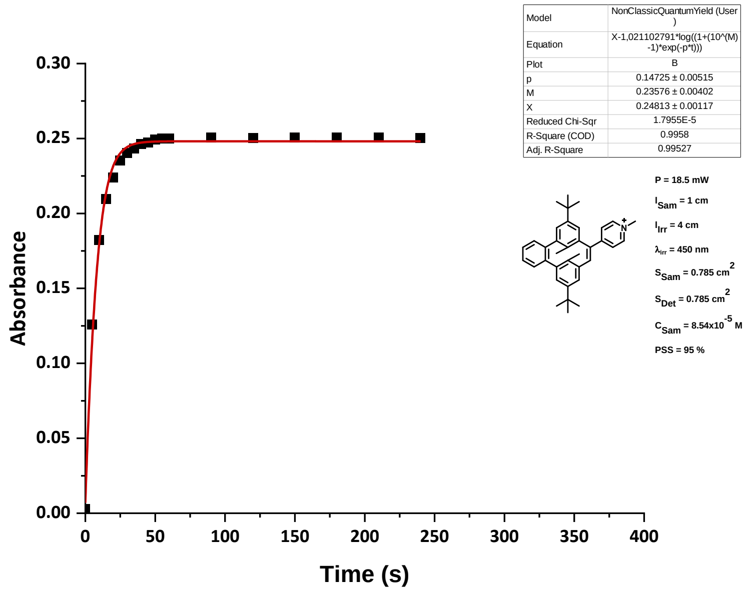

Figure S35: Evolution of the absorbance at λ = 660 nm during an irradiation at λ_{ex} = 450 nm of **BCPDPy$^+$, PF$_6^-$** in acetonitrile as function of time and its fit using Maafi and Brown photo-kinetic model.

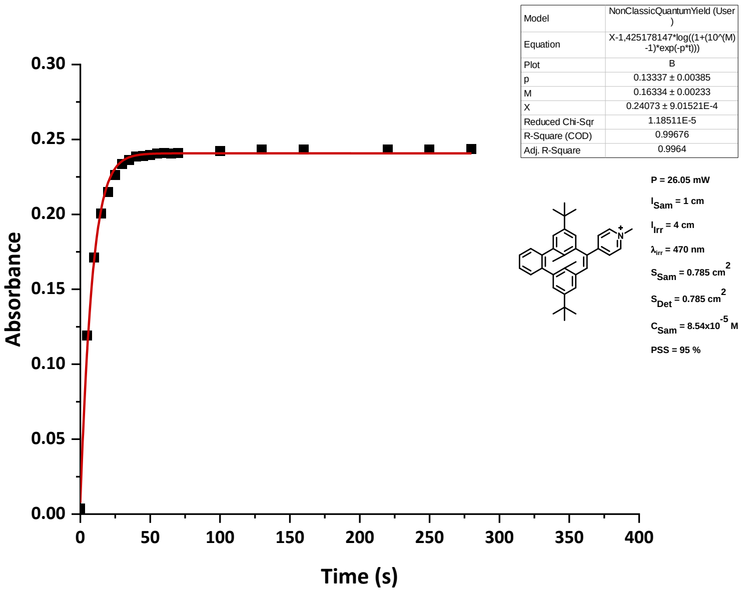

Figure S36: Evolution of the absorbance at λ = 660 nm during an irradiation at λ_{ex} = 470 nm of **BCPDPy$^+$, PF$_6^-$** in acetonitrile as function of time and its fit using Maafi and Brown photo-kinetic model.

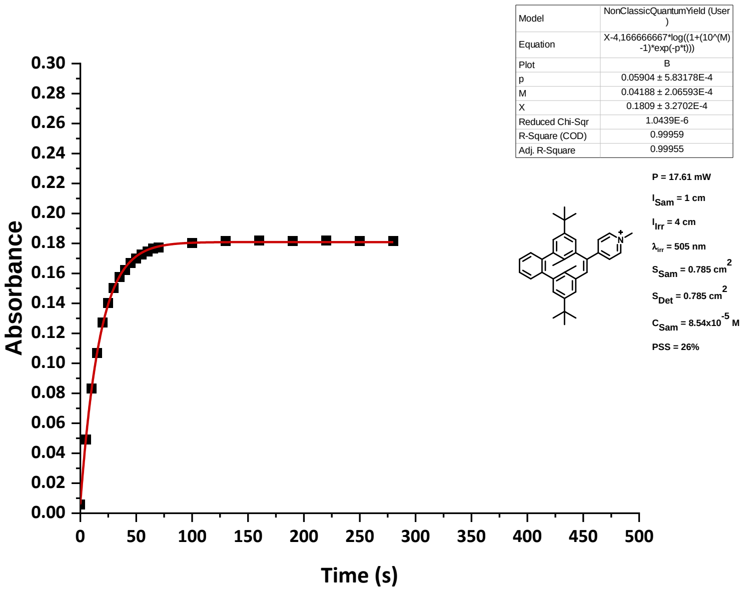

Figure S37: Evolution of the absorbance at λ = 660 nm during an irradiation at λ_{ex} = 505 nm of **BCPDPy$^+$, PF$_6^-$** in acetonitrile as function of time and its fit using Maafi and Brown photo-kinetic model.

C) Experimental study of the photo-opening process in water

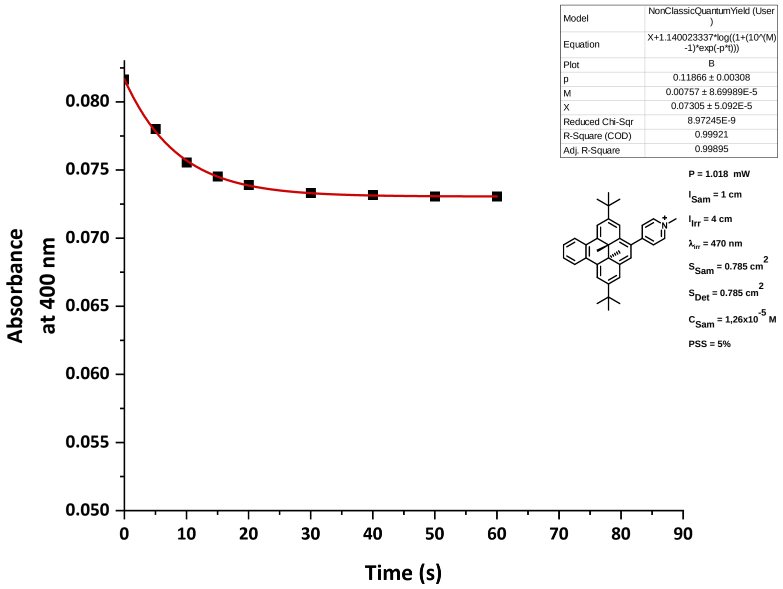

Figure S38: Evolution of the absorbance at λ = 400 nm during an irradiation at λ_{ex} = 470 nm of a solution of **BDHPPy$^+$, I$^-$** in water as function of time and its fit using Maafi and Brown photo-kinetic model.

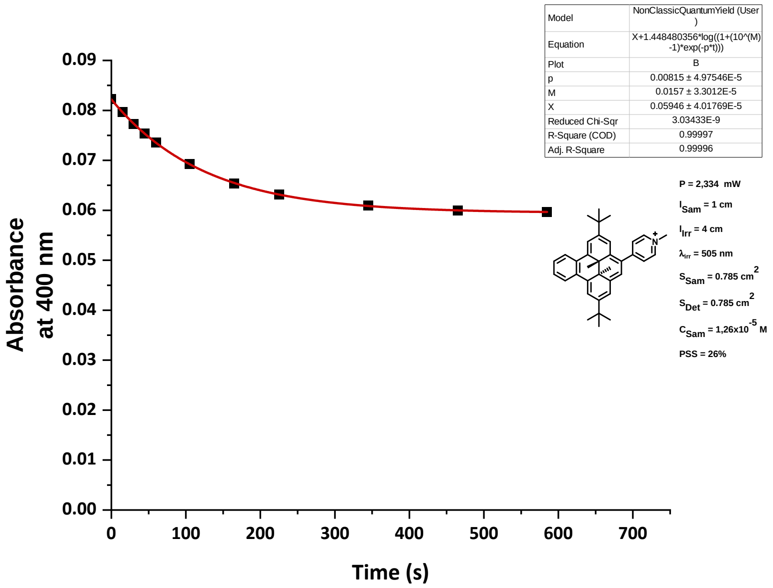

Figure S39: Evolution of the absorbance at λ = 400 nm during an irradiation at λ_{ex} = 505 nm of a solution of **BDHPPy$^+$**, I$^-$ in water as function of time and its fit using Maafi and Brown photo-kinetic model.

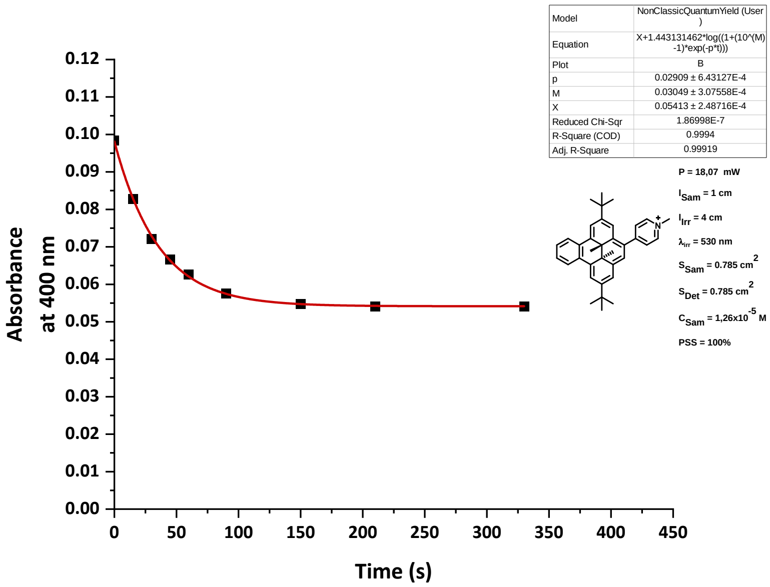

Figure S40: Evolution of the absorbance at λ = 400 nm during an irradiation at λ_{ex} = 530 nm of a solution of **BDHPPy$^+$, I$^-$** in water as function of time and its fit using Maafi and Brown photo-kinetic model.

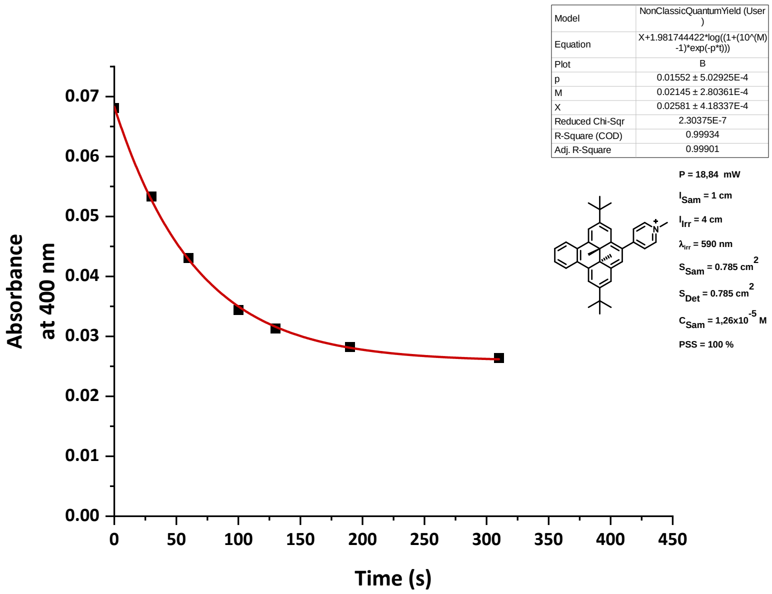

Figure S41: Evolution of the absorbance at λ = 400 nm during an irradiation at λ_{ex} = 590 nm of a solution of **BDHPPy$^+$, I$^-$** in water as function of time and its fit using Maafi and Brown photo-kinetic model.

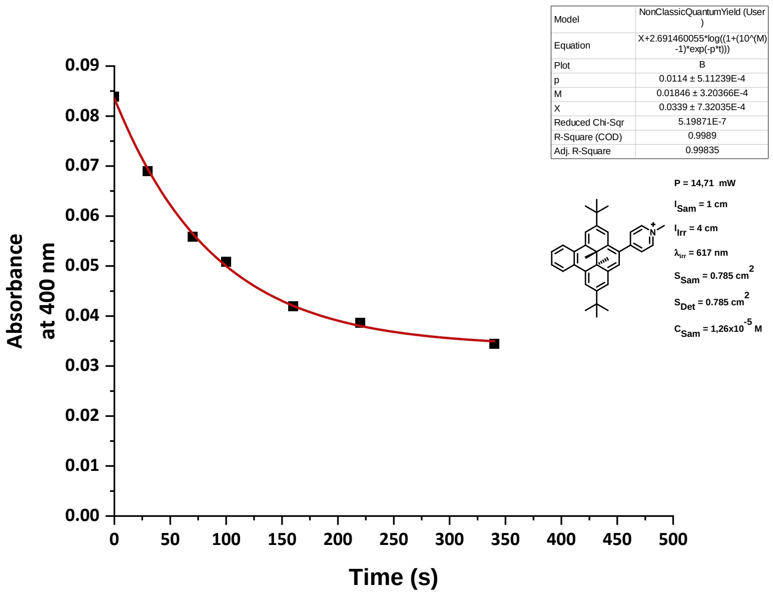

Figure S42: Evolution of the absorbance at λ = 400 nm during an irradiation at λ_{ex} = 617 nm of a solution of **BDHPPy⁺, I⁻** in water as function of time and its fit using Maafi and Brown photo-kinetic model.

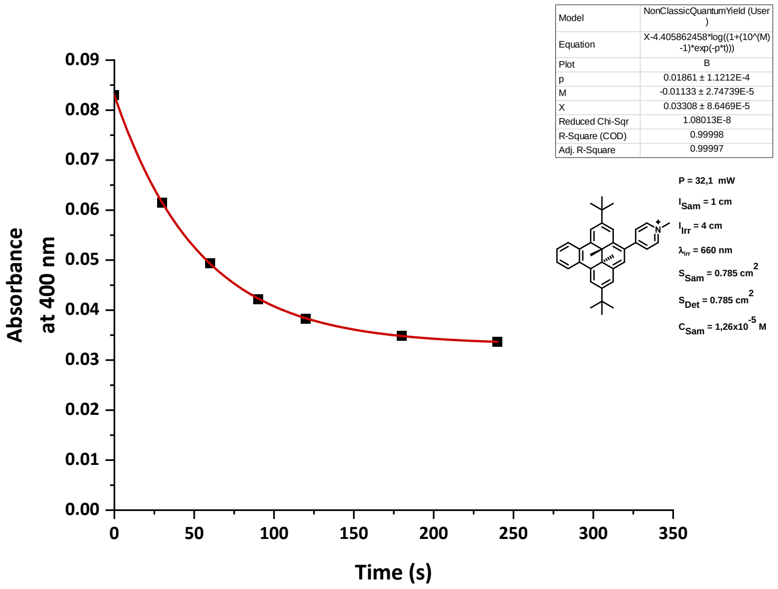

Figure S43: Evolution of the absorbance at λ = 400 nm during an irradiation at λ_{ex} = 660 nm of a solution of **BDHPPy⁺, I⁻** in water as function of time and its fit using Maafi and Brown photo-kinetic model.

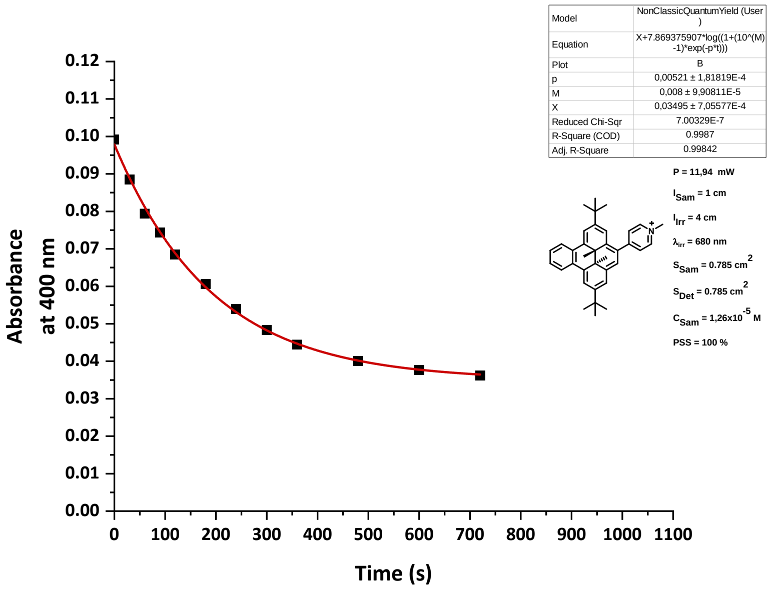

Figure S44: Evolution of the absorbance at λ = 400 nm during an irradiation at λ_{ex} = 680 nm of a solution of **BDHPPy⁺, I⁻** in water as function of time and its fit using Maafi and Brown photo-kinetic model.

D) Experimental study of the photo-closing process in water

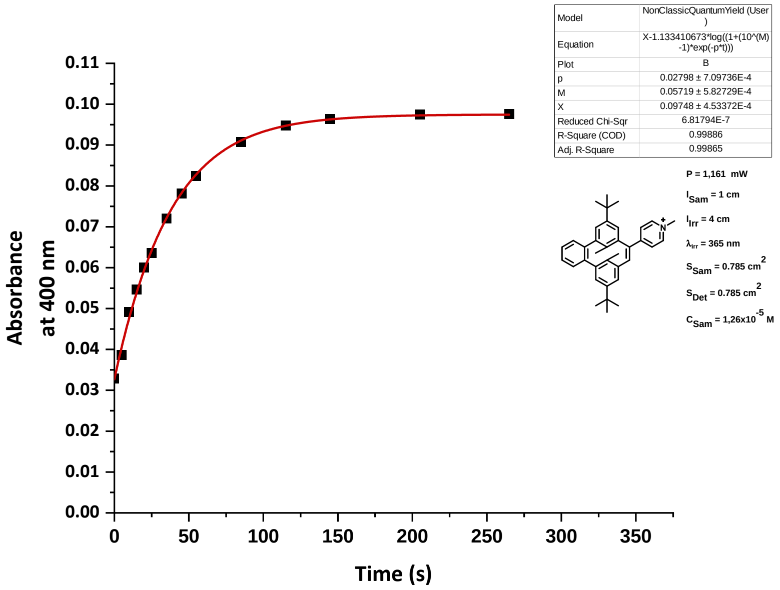

Figure S45: Evolution of the absorbance at λ = 400 nm during an irradiation at λ_{ex} = 365 nm of a solution of **BCPDPy⁺, I⁻** in water as function of time and its fit using Maafi and Brown photo-kinetic model.

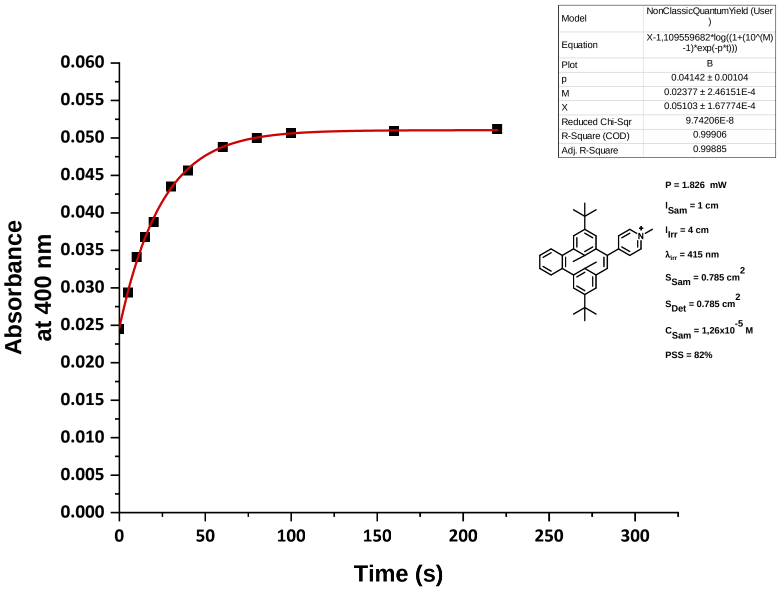

Figure S46: Evolution of the absorbance at λ = 400 nm during an irradiation at λ_{ex} = 415 nm of a solution of **BCPDPy$^+$, I$^-$** in water as function of time and its fit using Maafi and Brown photo-kinetic model.

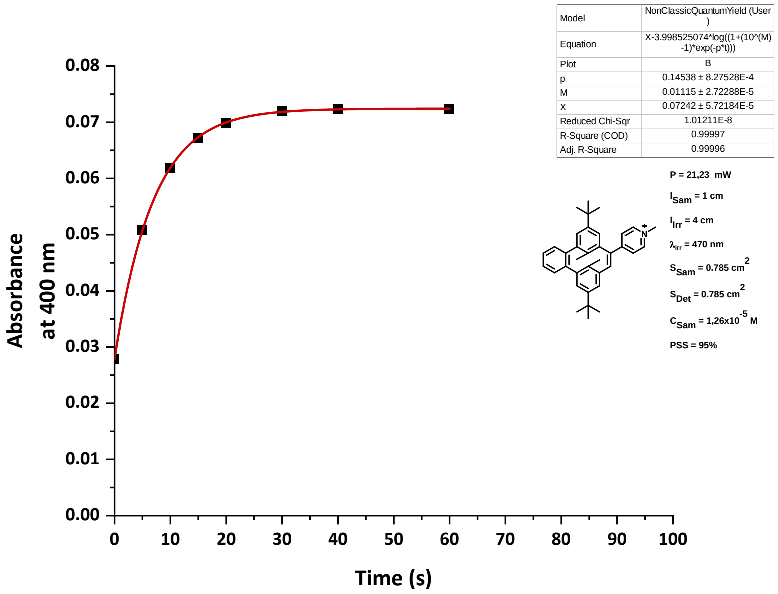

Figure S47: Evolution of the absorbance at λ = 400 nm during an irradiation at λ_{ex} = 470 nm of a solution of **BCPDPy$^+$ I$^-$** in water as function of time and its fit using Maafi and Brown photo-kinetic model.

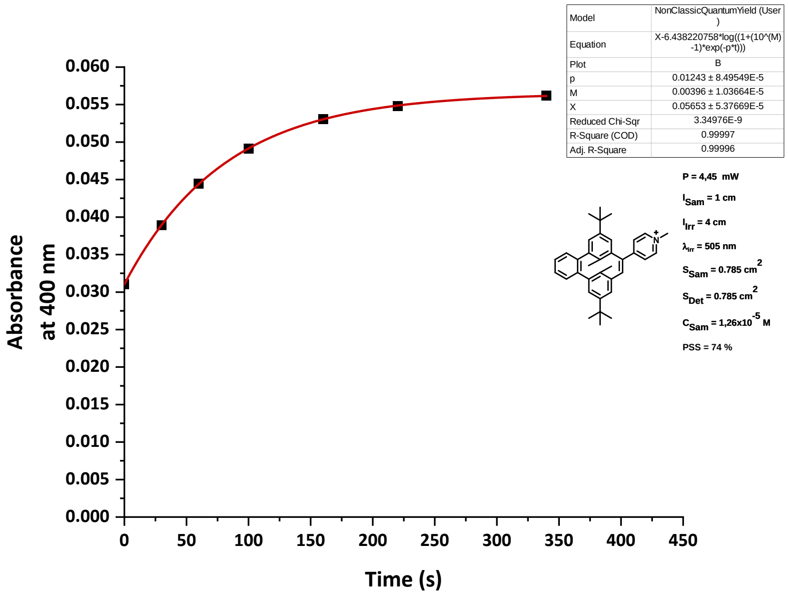

Figure S48: Evolution of the absorbance at λ = 400 nm during an irradiation at λ_{ex} = 505 nm of a solution of **BCPDPy$^+$ I$^-$** in water as function of time and its fit using Maafi and Brown photo-kinetic model.

9. Cartesian coordinates for optimized structures

B3LYP/6-311G(d,p) **BDHP** optimized Cartesian coordinates in acetonitrile.

C	2.858948222421	0.057803587395	-1.742081435292
C	3.552665609768	0.014079530971	-0.550972670941
C	2.812218142304	-0.036012143123	0.676768449249
C	1.450096547602	0.066286332874	0.748743347155
C	1.447886119456	0.096774923529	-1.800008804065
C	0.692845855995	0.009148058961	1.989752669756
C	-0.739214864856	-0.019089556074	1.975391987918
C	-1.471464727707	-0.068987923528	0.717809236121
C	-2.827925921524	0.040413788467	0.613807707594
C	-3.545262080274	-0.008018612823	-0.632397097211
C	-2.831034902595	-0.058084505726	-1.807526037440
C	-1.415474710815	-0.098253378302	-1.830794532876
C	-0.680737514618	0.000577945316	-2.991564500733
C	0.737557104750	0.000513466594	-2.977277281562
C	0.670016110195	0.386546807665	-0.518310213345
C	-0.665302618631	-0.389281757388	-0.533047572297
C	0.443189830844	1.939564662046	-0.501255576298
C	-0.438973841009	-1.942178180347	-0.511906940961
H	3.394299436356	0.002816227774	-2.684847923916
H	3.370575617914	-0.199591005143	1.586367883826
H	-3.411034611671	0.207160999000	1.509907551589
H	-3.340653254776	-0.009182254369	-2.762304462884
H	-1.201607816091	0.060768984235	-3.942590890113
H	1.278046463090	-0.055768822419	-3.917475113098
H	-0.099697755477	2.253826488506	0.391639004188
H	-0.113873722067	2.260595487257	-1.382506605927
H	0.079231639866	-2.258231696848	0.394878609673
H	0.142038492337	-2.261760004060	-1.378117490964
H	1.414659019052	2.436923790496	-0.505364276376
H	-1.409978081529	-2.439513761891	-0.543038769649
C	-1.420302336645	-0.042512378553	3.218680442795
H	-2.501119668165	-0.092745375332	3.229633791960
C	1.347567080990	0.023129237800	3.248144730315
H	2.427877600549	0.072257953713	3.282896654608
C	-0.751922773487	-0.030760082987	4.421584185151
H	-1.307890627942	-0.053506403912	5.351989852515
C	0.653716631811	0.003357573071	4.436205309887
H	1.190117587476	0.019246821790	5.378194246990
C	5.093273860883	-0.057537667122	-0.564715446083
C	5.545168412120	-1.352853518149	-1.280474279755
C	5.656520038789	1.167542162130	-1.323063541579
C	5.704254653602	-0.057333971584	0.849745727879
H	5.159439612004	-2.237121503344	-0.765792354523
H	5.194246349015	-1.387300535099	-2.314162564811
H	6.637784657176	-1.416972781376	-1.294809343211
H	5.360547656116	2.099130956106	-0.832871830822
H	6.750040350211	1.128019745665	-1.346637499096
H	5.301160034326	1.204935306425	-2.354964022346
H	6.794766082979	-0.069029227435	0.773739186839
H	5.421820315831	0.834883645284	1.415372077678
H	5.408664382052	-0.938461959359	1.425567888527
C	-5.079801663411	0.065026035440	-0.591760434978
C	-5.626786977447	-1.121243188025	0.238368010713
C	-5.522022264531	1.395667304397	0.063894429851
C	-5.709081750939	-0.006551809085	-1.994783094838
H	-5.343704893448	-2.075491568832	-0.214272555378
H	-5.249175393087	-1.112033452087	1.263277116571
H	-6.719232834146	-1.077489651028	0.285017514221
H	-5.152997325608	2.251603716999	-0.507513412301
H	-6.614275633571	1.453549653231	0.097161466418
H	-5.154742090801	1.492895229878	1.087919207578
H	-6.798494734640	0.023767926594	-1.908611807329
H	-5.404940177603	0.836172108767	-2.621186288866
H	-5.442650918768	-0.932425713027	-2.511549005702

B3LYP/6-311G(d,p) **BDHPPy$^+$** optimized Cartesian coordinates in acetonitrile.

C	2.258571533939	-1.126987395651	-0.054177902217
C	-2.073908364637	2.560128054369	-0.104350729805
C	2.012081161529	-2.489245338715	-0.116669639439
C	-3.371542420967	2.114842468435	-0.048098057682
C	0.666917813776	-2.956148450822	-0.051464076182
C	-3.610814973695	0.697562212891	0.049330071270
C	-0.407837200192	-2.127358501444	0.124595384496
C	-2.632079743931	-0.248508255229	-0.029725620443
C	1.245161559519	-0.167043817275	0.111912502368
C	-0.967970227039	1.668209258938	-0.115558167958
C	-1.783645475879	-2.600613989197	0.136490648086
C	-2.876076083525	-1.679098440983	0.086627835550
C	0.324275527332	2.105583548189	0.005928843130
C	1.450702023244	1.220770908280	0.056863382962
C	-0.178216753220	-0.652347597247	0.410133170745
C	-1.213370851288	0.189187101242	-0.374861141451
C	-0.342909036368	-0.490016846046	1.963902640727
C	-1.060097821773	-0.010155833296	-1.922907269812
C	-4.193676080626	-2.200303968129	0.116239523769
C	-2.075250959662	-3.985641373818	0.233440003496
C	-4.441874102263	-3.552050005083	0.204472431959
C	-3.368028917349	-4.457565987835	0.267474792312
H	3.269774372508	-0.782072359193	-0.220053344346
H	-1.853967797389	3.620484701946	-0.088024746481
H	0.494623235045	-4.009690868461	-0.207455960434
H	-4.630215448125	0.384295393305	0.229225943865
H	0.500272226582	3.172159292248	0.097552036305
H	-1.335121846631	-0.806869541387	2.288224015755
H	-1.252303123522	-1.043908827917	-2.214584095728
H	-0.188649616576	0.546306164149	2.265696497244
H	-0.056565416306	0.266183728534	-2.252044981422
H	0.398197204865	-1.112654057785	2.467841635015
H	-1.779264195505	0.629843661423	-2.436485382734
H	-5.036126783439	-1.525091411074	0.050939080004
H	-1.264368058291	-4.697431815467	0.309464508907
H	-5.463091661629	-3.914658155125	0.223087829505
H	-3.554862290281	-5.521936450975	0.349049890127
C	2.780804946124	1.836938904064	0.068682383723
C	3.058271066066	2.974802272194	-0.718928221924
C	3.826114809730	1.395386931309	0.908106150179
C	4.288255588489	3.585075837449	-0.671048999722
H	2.314814679108	3.375786851083	-1.393379501924
C	5.036141382955	2.044393492519	0.927845739238
H	3.682328979696	0.566258593847	1.585342099166
H	4.532304534034	4.445074764954	-1.278094891862
H	5.846660786457	1.738948028010	1.573832582833
N	5.266999954600	3.125275490134	0.143642104948
C	6.596187856505	3.771259549166	0.152371796164
H	7.008711621439	3.716864213100	1.157008337048
H	7.253380677877	3.256486461561	-0.548520573304
H	6.486002529481	4.812280415824	-0.139216683911
C	3.187456889926	-3.460024671063	-0.337565295050
C	3.847605226362	-3.162493932172	-1.706076084280
C	4.230587804165	-3.264942854757	0.788668345386
C	2.751086187636	-4.937442518385	-0.327571538720
H	3.130268768494	-3.291359163547	-2.521130171830
H	4.239236714164	-2.144248897239	-1.757369722283
H	4.680925832083	-3.850833472299	-1.874703719748
H	3.790611766930	-3.471884391285	1.768052201153
H	5.069417110016	-3.951181286006	0.641108333700
H	4.631044150300	-2.249238137858	0.805051777208
H	3.631953182737	-5.572532310076	-0.449342292283
H	2.271079988932	-5.214539984596	0.614908688612
H	2.065511842114	-5.170130577777	-1.146464622789
C	-4.580553954255	3.061210365831	-0.014240768458
C	-5.525028228802	2.722614568130	-1.192991022502
C	-5.343086379126	2.882473362753	1.321067107808
C	-4.171103189571	4.539677873877	-0.138137205865
H	-5.015154340108	2.857355472300	-2.150986741576
H	-5.887274054800	1.693189265481	-1.145277887129
H	-6.396477730554	3.383641879555	-1.175692011699
H	-4.698784965945	3.121549897497	2.171582599709
H	-6.206667933773	3.553331747705	1.349339332537
H	-5.712422853675	1.863028464272	1.452629416401
H	-5.066641944076	5.166229612789	-0.134855964407
H	-3.541749006730	4.859926430346	0.696407249189
H	-3.632546843240	4.734365905517	-1.069497955198

B3LYP/6-311G(d,p) **BCPDPy$^+$** optimized Cartesian coordinates in acetonitrile.

C	-0.861145220736	-1.924312748360	1.013748721603
C	0.446640974659	3.231136113360	-0.154707729900
C	0.136923530590	-2.888127562130	1.216075094329
C	1.697377316534	3.541218811856	-0.690199856158
C	1.392143194903	-2.588044090976	0.687128657805
C	2.677651690937	2.545994030781	-0.578261526328
C	1.609454463674	-1.493091781944	-0.168793454086
C	2.511840686205	1.400281364165	0.214244175995
C	-0.678155420838	-0.833294424651	0.159361790858
C	0.232967417130	2.063324338872	0.602738403927
C	3.031494057665	-1.048656474749	-0.362583542875
C	3.472693219243	0.263357881535	0.009199048357
C	-1.159975020654	1.588674902958	0.642357082752
C	-1.609530090566	0.337218083927	0.313926838903
C	0.506937669577	-0.735517154227	-0.606102151536
C	1.343396833061	1.276682163478	0.992558456033
C	0.579026978283	0.107266364769	-1.854328944262
C	1.301464884232	0.429581063900	2.234617834732
C	4.849143086668	0.525110712386	0.012250148868
C	3.994630562249	-1.988607236078	-0.751193851129
C	5.786976689312	-0.435064052077	-0.359117143240
C	5.355879224471	-1.694489665223	-0.760657673302
H	-1.789928284690	-1.991830149458	1.569242566809
H	-0.411282311921	3.854191127190	-0.374409305552
H	2.251793944461	-3.179843995425	0.972850572737
H	3.596547297061	2.638003182418	-1.145351170708
H	-1.905608825077	2.376194707864	0.705041056295
H	1.606758685795	0.285700177132	-2.165886659957
H	2.057526711676	-0.352641547377	2.234458349656
H	0.067640685735	1.064233497860	-1.766839052110
H	0.325313769797	-0.015204147145	2.419610564502
H	0.088530089719	-0.447624090968	-2.662716415106
H	1.518628455283	1.088740863166	3.083979557988
H	5.189527784763	1.508505765697	0.316154463685
H	3.662589323048	-2.975622652082	-1.052922534145
H	6.843674838243	-0.194398953463	-0.339185704881
H	6.069822210847	-2.448349147144	-1.071978914108
C	-3.051854871337	0.116182325715	0.151240069987
C	-4.031236740066	0.879900609196	0.820009379595
C	-3.528625917788	-0.892289811442	-0.710617627580
C	-5.368047241152	0.652149537491	0.601685659696
H	-3.758197580767	1.641410510952	1.536375282781
C	-4.876786429758	-1.078403525230	-0.898880353938
H	-2.839566377792	-1.526583857813	-1.249928085054
H	-6.140641805348	1.215929850885	1.104610210685
H	-5.270371346712	-1.832279064924	-1.565330899321
N	-5.786031677530	-0.312365007394	-0.251791145340
C	-7.231122976858	-0.571022613141	-0.426659709416
H	-7.402771396517	-0.951211872413	-1.430398516216
H	-7.558240801239	-1.304366497827	0.310314018222
H	-7.775611513132	0.359843119376	-0.291519446343
C	-0.141051452866	-4.129031628531	2.081103145744
C	-0.530842573066	-3.698678658342	3.514512116738
C	-1.308324463207	-4.926111119115	1.453077643387
C	1.082664414532	-5.058730664950	2.172500333401
H	0.276873319381	-3.130479287050	3.984079871197
H	-1.430392023387	-3.078811140208	3.523749828777
H	-0.728711448723	-4.580588306920	4.131123301162
H	-1.055722566586	-5.255653863742	0.441267808553
H	-1.526908032235	-5.813264008014	2.054974125369
H	-2.220457463527	-4.327343470794	1.394856702196
H	0.823294508055	-5.946074845373	2.755796630573
H	1.411509208323	-5.393300616127	1.184817486271
H	1.927580845677	-4.574587584059	2.669484284264
C	1.984492172451	4.836767534184	-1.468172279723
C	3.177946460209	5.565191903759	-0.807010679870
C	2.339162398710	4.504832598246	-2.936323646227
C	0.776905852311	5.791762965327	-1.467279984514
H	2.950024232793	5.824913036568	0.230643687146
H	4.079121779524	4.947464173814	-0.809753357327
H	3.402877353036	6.489553281068	-1.347445019243
H	1.511735854920	3.987471730764	-3.429842639590
H	2.543405249513	5.425485359169	-3.491138065223
H	3.225599585856	3.870406226589	-3.008206144504
H	1.041763686622	6.714618899866	-1.989711591090
H	-0.086132405997	5.359879030800	-1.981257965177
H	0.473191582336	6.059481219803	-0.451475626502

Broken-symmetry B3LYP/6-311G(d,p) transition state optimized Cartesian coordinates in acetonitrile for **BDHPPy⁺/BCPDPy⁺** thermal conversion.

C	-0.960259443707	-1.911373757234	0.951851236732
C	0.473820680010	3.323995143200	-0.136069490336
C	0.018983676093	-2.895199723138	1.198971101890
C	1.788021377988	3.658356493231	-0.454020375561
C	1.342026078756	-2.584592255928	0.819063157263
C	2.760102043459	2.621758586640	-0.362884429382
C	1.676850119400	-1.442430375074	0.113930502896
C	2.502150781019	1.386743422461	0.189701642489
C	-0.696429983570	-0.755084338827	0.238453484957
C	0.121802997148	2.059580845675	0.359082158939
C	3.101792161697	-1.060026072647	-0.049113710789
C	3.508655415638	0.296595838822	0.122663659769
C	-1.251946193173	1.659780600879	0.323268132948
C	-1.689953200512	0.354159679296	0.194228589482
C	0.640423364907	-0.502121331402	-0.296712551873
C	1.169824941734	1.096419383008	0.722867275661
C	0.704009509072	-0.032810366607	-1.752143534784
C	1.135406400963	0.636887311632	2.181137227981
C	4.879776940843	0.584487727517	0.143219237672
C	4.082384806976	-2.034146819501	-0.272910835046
C	5.839933460126	-0.405224120848	-0.054262982295
C	5.439156767143	-1.718025293116	-0.282290352641
H	-1.955788592965	-2.046536898038	1.353950520668
H	-0.327849607882	4.027144418167	-0.325898966510
H	2.143636867753	-3.246337789132	1.125322392742
H	3.747742557370	2.805455712404	-0.767944406489
H	-1.975640522848	2.460038781368	0.209540412919
H	1.712095025905	0.269195394826	-2.035176348887
H	1.858648278420	-0.155459664211	2.373539226252
H	0.024393065954	0.797686350566	-1.941847829422
H	0.143502383464	0.289347507416	2.471004343315
H	0.408872568190	-0.864885805393	-2.398355223591
H	1.392665104904	1.489275061952	2.817286222707
H	5.203108625728	1.601106916381	0.331785254356
H	3.775220417078	-3.057915673517	-0.451604759214
H	6.892357344396	-0.147409815487	-0.031695053664
H	6.173765327930	-2.495073166534	-0.458682810217
C	-3.099504268921	0.085500981390	-0.021574540621
C	-4.121040591301	0.956757273777	0.433950486025
C	-3.540832072759	-1.060311463168	-0.728718770954
C	-5.440543708305	0.692044293113	0.178701906167
H	-3.886945370872	1.835213407599	1.018021629474
C	-4.873703280965	-1.275177058707	-0.965250439908
H	-2.830447932510	-1.774517673250	-1.119014073621
H	-6.236562825647	1.334627305772	0.526361489354
H	-5.231959066346	-2.130241924853	-1.520545317387
N	-5.819603473140	-0.410229677042	-0.518043700657
C	-7.250698561712	-0.700343551728	-0.728553569127
H	-7.628167341942	-1.303415873801	0.097820600140
H	-7.798710942380	0.237328032496	-0.781628871181
H	-7.368687413270	-1.241617000603	-1.664211193108
C	-0.282761093393	-4.211684942053	1.926849035559
C	0.485519225392	-4.252831340089	3.270345648452
C	-1.783477696193	-4.385340952969	2.224601647496
C	0.173581324229	-5.397207520404	1.042499294584
H	1.565964402787	-4.186240464466	3.124073794805
H	0.179917941045	-3.427484396346	3.919245572625
H	0.276566143952	-5.191330070961	3.792438873372
H	-2.383971299643	-4.361551546612	1.311062661130
H	-1.948490848296	-5.352765854637	2.705586209750
H	-2.158015223339	-3.614064629613	2.902870171494
H	-0.027389061205	-6.344020009057	1.552336655227
H	-0.364117816411	-5.402754362972	0.090278953061
H	1.243411011939	-5.355333689789	0.826023409912
C	2.200626703134	5.039196370587	-0.979052928338
C	3.319529709823	5.610716284434	-0.074856578109
C	2.731225952788	4.910336039790	-2.427557664588
C	1.027863508911	6.036402720087	-0.981185295250
H	2.967788934673	5.726867857810	0.953994989352
H	4.200912228692	4.965563569019	-0.060080897731
H	3.630981658798	6.593300357175	-0.441135922094
H	1.959281767247	4.510833347682	-3.090965324598
H	3.028894547649	5.893129044996	-2.804770678398
H	3.602281298650	4.253896873146	-2.487289826319
H	1.381031778793	7.012093225147	-1.324318980955
H	0.225763310969	5.722233316291	-1.654315962392
H	0.606275173670	6.168041734003	0.019094996420

SF-TD-BHHLYP/6-311G(d,p) model **BDHPPy+** optimized Cartesian coordinates.

C	2.0831328212	-0.3234879087	-1.0813999709
C	-3.3165430797	0.1393011259	0.5972121479
C	3.0333532832	-0.4978397955	-0.1266621516
C	-3.5780424386	0.2427278973	1.9111743956
C	2.7515017594	-0.4027373566	1.2584135125
C	-2.5293654647	0.2418577290	2.8910202946
C	1.5170106170	-0.0862237891	1.7079272204
C	-1.2451575403	0.0135305176	2.5684612371
C	0.7514227506	-0.0115782944	-0.7390350009
C	-1.9713738796	0.0026152053	0.1363817199
C	1.1821626280	-0.0328023572	3.1229135036
C	-0.1612998528	0.0111131688	3.5432742609
C	-1.6505255891	0.1120363906	-1.1666859541
C	-0.2950220141	0.0379778327	-1.6464948649
C	0.4344508128	0.3024763773	0.7221071135
C	-0.8937231222	-0.3600661343	1.1387333994
C	0.3553605519	1.8576191555	0.7859919321
C	-0.7900559042	-1.9102912098	1.1107020186
C	-0.4289723550	0.0297421731	4.9201563717
C	2.1958791596	-0.0060002953	4.0955776907
C	0.5771878830	0.0401985586	5.8485590295
C	1.9064865599	0.0317483916	5.4323069168
H	2.3447648274	-0.5238340789	-2.1030285042
H	-4.1079370911	0.2122110498	-0.1292829525
H	3.5398568211	-0.6518953921	1.9430158947
H	-2.8096135984	0.4613878803	3.9042722161
H	4.0284624775	-0.7791770375	-0.4257539663
H	-4.5902317403	0.3813983748	2.2489233924
H	-2.4380084126	0.3059610507	-1.8746022427
H	0.1161178017	2.1916186045	1.7879212577
H	-0.0638812837	-2.2722140701	1.8287055773
H	-0.3974731619	2.2352703997	0.1057461278
H	-0.5085296301	-2.2603901759	0.1238084544
H	1.3167024016	2.2756679740	0.5100320847
H	-1.7547779074	-2.3350345904	1.3617444679
H	-1.4452198059	0.0181817238	5.2654691699
H	3.2253929774	0.0109874616	3.7917698085
H	0.3387916337	0.0545767824	6.8974524778
H	2.7020597494	0.0569687185	6.1556048433
C	-0.1100325181	0.0283240300	-3.0830942606
C	-1.0390379898	-0.6112763718	-3.9264994362
C	0.9323631564	0.7128884208	-3.7370830355
C	-0.8975352479	-0.5790690116	-5.2793988448
H	-1.8616450485	-1.1616952301	-3.5121081269
C	1.0178713630	0.7245691421	-5.0949875543
H	1.6494106640	1.2808891336	-3.1773055527
H	-1.5813226378	-1.0812097966	-5.9379126855
H	1.7908655040	1.2602397841	-5.6135165880
N	0.1183570840	0.0821132777	-5.8654141725
C	0.2698178225	0.0653704472	-7.3222488510
H	0.7414140363	0.9840387818	-7.6410536148
H	0.8767369566	-0.7803922583	-7.6228513131
H	-0.7062314595	-0.0052526261	-7.7803155535

SF-TD-BHHLYP/6-311G(d,p) model **BDHPPy^{+*}** S_1 optimized Cartesian coordinates.

C	1.9821133422	-0.5560614411	-1.0823336215
C	-3.2814385032	0.1331207144	0.6161106402
C	2.9640190181	-0.7329415537	-0.1357921436
C	-3.5742018752	0.2209185504	1.9820349381
C	2.7205503025	-0.5431792780	1.2379641432
C	-2.5589916668	0.2025145524	2.9299375616
C	1.5083577700	-0.1342349025	1.6899166463
C	-1.2392500237	-0.0022843437	2.5900737562
C	0.7040269069	-0.1563029660	-0.7251325724
C	-1.9971859147	-0.0727363829	0.1882977749
C	1.1935384859	0.0032103171	3.1065096360
C	-0.1517437395	0.0585697825	3.5444191958
C	-1.6627723411	-0.0104042612	-1.1763663384
C	-0.3681526372	-0.0663635773	-1.6616283146
C	0.4236478266	0.2339511516	0.7118257375
C	-0.9107318408	-0.4101308451	1.1770234523
C	0.3313708491	1.7888158124	0.7342068826
C	-0.7854455470	-1.9639115864	1.1849851541
C	-0.3972115622	0.1669061969	4.9200876027
C	2.2150228772	0.0962637741	4.0548100364
C	0.6263875414	0.2393794001	5.8333688869
C	1.9443769179	0.2122578755	5.3984875850
H	2.1988908920	-0.7993053272	-2.1057384318
H	-4.0662174226	0.2826279661	-0.1061941829
H	3.5067895675	-0.7906330124	1.9265187446
H	-2.8301211700	0.3917694219	3.9515943646
H	3.9320743163	-1.0870628926	-0.4439376692
H	-4.5887312227	0.3820422369	2.2985680528
H	-2.4716232674	0.1318552868	-1.8735967064
H	0.1204705106	2.1491777106	1.7333709489
H	-0.0280365845	-2.2890052377	1.8883756058
H	-0.4501464488	2.1310147081	0.0664698792
H	-0.5246064286	-2.3318130099	0.1996631920
H	1.2744253773	2.2140908006	0.4110435459
H	-1.7334755346	-2.3990651244	1.4769400115
H	-1.4062723727	0.1812062299	5.2853181626
H	3.2396458668	0.1058631454	3.7332208455
H	0.4027614526	0.3201299956	6.8822307320
H	2.7519477959	0.2874095107	6.1046371532
C	-0.1552046358	0.0025846365	-3.1021221049
C	-1.0231423764	-0.6498920793	-4.0081794533
C	0.8790559613	0.7647729679	-3.6904755956
C	-0.8533014878	-0.5416633526	-5.3483309614
H	-1.8217594843	-1.2722938751	-3.6489015066
C	1.0069968905	0.8552478141	-5.0372040702
H	1.5649049018	1.3213711037	-3.0798228087
H	-1.4913549269	-1.0439138196	-6.0503835714
H	1.7713950330	1.4478768276	-5.5025629342
N	0.1499952378	0.2113352166	-5.8802104880
C	0.3594013596	0.2503004741	-7.3185125430
H	0.8115356554	1.1945916515	-7.5906083059
H	1.0064031564	-0.5612391405	-7.6379518137
H	-0.5936114682	0.1664019572	-7.8233813909

SF-TD-BHHLYP/6-311G(d,p) S_0/S_1 MECI optimized Cartesian coordinates for model **BDHPPy$^+$**/**BCPDPy$^+$** photochemical conversion.

C	-1.0389010459	1.9066213225	0.9230284056
C	-0.4097097814	-3.2862462804	-0.5688644930
C	-1.1206360349	2.8862739923	-0.0686960208
C	-0.0498377391	-3.5863892899	-1.8384347183
C	-0.6134177590	2.6604315297	-1.3358874662
C	0.1597386254	-2.5371308181	-2.7780804774
C	0.0485532523	1.4861983041	-1.6273637729
C	-0.1578220153	-1.2485167219	-2.5174004711
C	-0.3484489053	0.7460910977	0.7057380421
C	-0.6474527758	-1.9451843269	-0.1705874693
C	0.3775478053	1.1495352210	-3.0185113978
C	0.1471051786	-0.1643695074	-3.4762994122
C	-0.5974686840	-1.6304818848	1.1749840810
C	-0.3417930023	-0.3554000048	1.6996350342
C	0.2553736042	0.4838115130	-0.5973808140
C	-0.7660918784	-0.8979155363	-1.2137345611
C	1.6840790991	-0.0666742149	-0.5367130816
C	-2.1970976261	-0.3884084411	-1.3583589903
C	0.2686774528	-0.4132018137	-4.8352400836
C	0.7939049644	2.1270828381	-3.9117177093
C	0.6749968533	0.5742261362	-5.7176581327
C	0.9593566530	1.8429001700	-5.2552654062
H	-1.5284547935	2.0705018579	1.8665238409
H	-0.4530613553	-4.0568123385	0.1824488170
H	-0.7813674678	3.3848027396	-2.1122989461
H	0.5987862124	-2.7917421082	-3.7265049681
H	-1.6381584800	3.8065965165	0.1387598988
H	0.1562668078	-4.6009314580	-2.1261068638
H	-0.6017711987	-2.4680535134	1.8509239503
H	2.0456805950	-0.3183824838	-1.5237879149
H	-2.2776222935	0.3954941839	-2.1007102937
H	1.7359745528	-0.9460567119	0.0895834646
H	-2.5943034108	-0.0376885616	-0.4140531453
H	2.3371033369	0.6935599383	-0.1201719219
H	-2.8093723779	-1.2223595535	-1.6869370813
H	0.0275886625	-1.3870687810	-5.2205938391
H	1.0063364431	3.1177612171	-3.5497365841
H	0.7642052846	0.3462717391	-6.7651351621
H	1.2901368498	2.6104137403	-5.9318181555
C	-0.0684515698	-0.1517331090	3.0581325967
C	-0.3109330852	-1.1373093734	4.0665067664
C	0.5050273559	1.0566039082	3.5600179094
C	-0.0094500289	-0.9128272129	5.3618785703
H	-0.7669859337	-2.0770491057	3.8252549813
C	0.7952783440	1.2132841860	4.8703398525
H	0.7486394141	1.8612963946	2.8946143282
H	-0.2057245497	-1.6455164768	6.1226640214
H	1.2459857834	2.1137347026	5.2445861717
N	0.5489363353	0.2479485440	5.7871488700
C	0.7872087035	0.4788930339	7.2060698077
H	1.5667465749	1.2192028970	7.3208793593
H	-0.1148493981	0.8297316632	7.6959624158
H	1.1147508665	-0.4409937409	7.6717249694

SF-TD-BHHLYP/6-311G(d,p) model **BCPDPy+** optimized Cartesian coordinates.

C	-0.9423435496	1.9456176987	0.9225215218
C	0.0182222672	-3.1898672369	-0.4854955593
C	-1.1167543061	2.8985665854	-0.0624756862
C	0.5310928528	-3.4590099647	-1.7386086177
C	-0.6222778524	2.6376611929	-1.3244252544
C	0.4576946227	-2.4730424204	-2.7019865885
C	0.1911385056	1.5330146055	-1.5711163597
C	-0.2935049336	-1.3095393702	-2.5087565109
C	-0.1223422456	0.8440772730	0.7031579836
C	-0.6922329546	-2.0109905140	-0.2526893370
C	0.3579193691	1.1080301789	-2.9956924123
C	-0.0645657322	-0.1693840258	-3.4497792460
C	-0.6758584632	-1.5589765019	1.1358322247
C	-0.2938986798	-0.3365622025	1.6050761805
C	0.6122681364	0.7511705776	-0.4897414428
C	-1.0497954513	-1.1910021588	-1.3442780469
C	1.8163479182	-0.1369191795	-0.6040348519
C	-2.2470545332	-0.2976488704	-1.2683264357
C	-0.0931144510	-0.4079071113	-4.8179628648
C	0.7670133990	2.0439516405	-3.9367038030
C	0.3001718363	0.5476921206	-5.7365141135
C	0.7508213018	1.7737716536	-5.2926336688
H	-1.4932961305	2.0271175956	1.8445860818
H	0.2304250439	-3.8520699257	0.3371281964
H	-0.9110038248	3.2654431139	-2.1492941607
H	1.0085598763	-2.5859738572	-3.6194952158
H	-1.7278433102	3.7651118816	0.1161323734
H	1.0697163405	-4.3698299833	-1.9274575510
H	-0.7370924976	-2.3568467740	1.8593599380
H	2.0998641743	-0.2995016105	-1.6336843382
H	-2.2359870304	0.4791085241	-2.0180343871
H	1.7002524191	-1.0962875442	-0.1199273663
H	-2.3915523956	0.1472218746	-0.2938438846
H	2.6495409250	0.3717934843	-0.1227143246
H	-3.1164916943	-0.9222011044	-1.4656383978
H	-0.4349401456	-1.3664498390	-5.1685649836
H	1.1064582539	3.0062606955	-3.5939586333
H	0.2597475317	0.3286000517	-6.7889232204
H	1.0800643264	2.5211248345	-5.9928530499
C	-0.0815894612	-0.1646860434	3.0312177985
C	-0.6950019941	-0.9633825289	4.0134254861
C	0.7767254870	0.8401819911	3.5089947257
C	-0.4263714302	-0.7726566462	5.3342676280
H	-1.4084456858	-1.7198573328	3.7496098354
C	1.0141911910	0.9816482621	4.8422288951
H	1.2671516898	1.5025162581	2.8219957766
H	-0.8886858566	-1.3650110158	6.1014879798
H	1.6814932630	1.7293718729	5.2280108108
N	0.4239523994	0.1828646180	5.7501122559
C	0.6513125885	0.3977868146	7.1824974628
H	1.6519729958	0.7780949928	7.3292619424
H	-0.0702054600	1.1069117621	7.5698404915
H	0.5529337747	-0.5442823726	7.7022215237

TD-ωB97X-D/6-311G(d,p) model **BDHPPy^{+*}** S_1 optimized Cartesian coordinates.

C	1.928662321211	-0.832585084641	-1.039200721623
C	-3.317359431242	-0.003392628842	0.626897030003
C	2.928875276377	-0.987420129515	-0.083287932506
C	-3.570953113658	0.250149635488	1.947355885636
C	2.698774422669	-0.637252132409	1.252593730864
C	-2.515709222994	0.300924269534	2.896387505969
C	1.507849623977	-0.085186941105	1.655343269713
C	-1.235544536018	-0.033519584625	2.572662885691
C	0.705506238163	-0.273419457215	-0.725609342972
C	-2.006697347668	-0.275714388930	0.186987465647
C	1.211471995834	0.142985964582	3.072551545886
C	-0.125969323360	0.074900419995	3.535161638948
C	-1.703068462633	-0.261124725024	-1.172717146623
C	-0.395099074812	-0.219815436052	-1.678669096090
C	0.428899397206	0.214895989883	0.668441639012
C	-0.918358049152	-0.521752521020	1.189526262318
C	0.174645901535	1.747417822326	0.640316000823
C	-0.654225785405	-2.050728491791	1.255536237875
C	-0.362177316099	0.144130961988	4.909749101790
C	2.235731761390	0.375258873115	3.994862165776
C	0.671285858804	0.343178126750	5.813292251860
C	1.973368762446	0.480990237619	5.351984798590
H	2.103967017846	-1.208487019428	-2.041307411256
H	-4.115271205096	0.062585674835	-0.105031638776
H	3.462928977934	-0.862031077960	1.987004408218
H	-2.753300004989	0.639756771864	3.897036190538
H	3.871968245735	-1.441788494678	-0.361561488175
H	-4.578642577945	0.481342003346	2.270901114470
H	-2.537981096516	-0.143270241266	-1.855925783182
H	-0.062194466626	2.118338604639	1.639049864299
H	0.149793953635	-2.271316811664	1.960579239007
H	-0.654933406318	1.986091976997	-0.028135412113
H	-0.375469804213	-2.428939497061	0.269952660571
H	1.071894244063	2.258245548499	0.285975113621
H	-1.560084755007	-2.562514749449	1.585285529319
H	-1.368360943945	0.020517357304	5.290068563080
H	3.253170449232	0.496271427851	3.643281586119
H	0.457413691228	0.393577949370	6.873916831301
H	2.784571499756	0.663085118450	6.046363802456
C	-0.189127548893	-0.052109284355	-3.083239506718
C	-1.142080401471	-0.473372884618	-4.053338375726
C	0.969630001448	0.579912121921	-3.617334016563
C	-0.935968967603	-0.268698194445	-5.382965368354
H	-2.037130340320	-1.003347407416	-3.758591807771
C	1.117058768167	0.768862911165	-4.957340006163
H	1.736729596408	0.962582100673	-2.958505153614
H	-1.641551660940	-0.601601090654	-6.132670679297
H	1.978881332829	1.271942071655	-5.375755675919
N	0.180019498385	0.351116614669	-5.847375112754
C	0.404763280369	0.494046146133	-7.287836372207
H	1.016043630907	1.376230442161	-7.470805579789
H	0.910704862353	-0.389278819093	-7.681838400183
H	-0.551976436986	0.622469730439	-7.791378951026

TD-ωB97X-D/6-311G(d,p) S_1 transition state optimized Cartesian coordinates for **BDHPPy+**/**BCPDPy+** photochemical conversion.

C	0.982859225977	1.910086507375	0.970885608728
C	-0.592564546824	-3.310963853041	0.268668592410
C	0.006429711115	2.906832739001	1.079799755341
C	-1.889198284276	-3.587969370214	-0.056199115427
C	-1.292601325090	2.677117308946	0.629624610413
C	-2.834334203489	-2.535260646706	-0.201850895554
C	-1.636585171916	1.490284475953	0.019012308108
C	-2.541870059950	-1.247493309197	0.126449759346
C	0.718110337996	0.718060225006	0.342542378788
C	-0.175184366244	-1.984030252663	0.523263575761
C	-3.042176625211	1.176824451795	-0.274200721986
C	-3.506642378639	-0.148886287378	-0.096744330915
C	1.185913550353	-1.678012335591	0.503577411563
C	1.698535213412	-0.387564534061	0.324123402093
C	-0.632438941555	0.431883393882	-0.203548456964
C	-1.195520176124	-0.904334936153	0.673625301776
C	-0.573960258485	0.012785586808	-1.691294468197
C	-1.309693314030	-0.490361653556	2.156511549754
C	-4.876079302667	-0.389210700457	-0.188957948532
C	-3.945120823488	2.176965283849	-0.632239246167
C	-5.767812644625	0.623448521612	-0.521574122110
C	-5.299956965027	1.905190295943	-0.767349845398
H	1.960522834110	2.079367265561	1.408819462289
H	0.150730727641	-4.101276409681	0.271700700803
H	-2.054108466618	3.427005316017	0.808701942611
H	-3.810816637173	-2.781226718869	-0.601544892368
H	0.248272370873	3.844641998409	1.565181271604
H	-2.192481590090	-4.604315082272	-0.274571174034
H	1.864708197126	-2.523721971258	0.462724481773
H	-0.185490170376	0.842694812308	-2.285058928602
H	-2.032594662452	0.317275671643	2.285116468482
H	-1.568391914659	-0.239320513875	-2.060842649902
H	-0.340980761885	-0.170394748568	2.544645986926
H	0.078556682228	-0.850981717684	-1.824254044732
H	-1.647102220147	-1.351485444835	2.736608586455
H	-5.263283544897	-1.378909771702	0.019590845195
H	-3.580936581151	3.179585442103	-0.824138135860
H	-6.826774960558	0.405509509852	-0.588879644769
H	-5.984945799607	2.695099191618	-1.050099856576
C	3.075462355281	-0.173029049517	0.068296573864
C	4.087841862785	-1.129785138909	0.386488775624
C	3.561384030468	1.014439515104	-0.559143892996
C	5.394683306866	-0.902043773907	0.094229375528
H	3.845444388684	-2.048758335757	0.901093738824
C	4.881624658005	1.180022470797	-0.838016989030
H	2.875511147049	1.793982765239	-0.859917838740
H	6.171089306849	-1.612068771739	0.347216667830
H	5.255833718555	2.065057461861	-1.335726448102
N	5.808117225748	0.238143773148	-0.520961982248
C	7.232389480425	0.479884022523	-0.757291895131
H	7.347819046852	1.140076714692	-1.615365068395
H	7.691876805187	0.939018970642	0.120113284511
H	7.727906235600	-0.464635291125	-0.976732183520

Unrestricted B3LYP/6-311G(d,p) **BDHPPy•** optimized Cartesian coordinates in acetonitrile.

C	2.229115917097	-1.129916660037	-0.193062154792
C	-2.069826464515	2.540790662844	-0.262740454753
C	1.992823009606	-2.523694972108	-0.211429947873
C	-3.363098224958	2.108031846414	-0.157543542862
C	0.672774933406	-2.974683039460	-0.009182092490
C	-3.596493937898	0.692639145612	0.047303642824
C	-0.390196980017	-2.128733130700	0.237400952020
C	-2.621782251543	-0.255592963092	0.013201984483
C	1.260990405587	-0.180540730325	0.071529208288
C	-0.956722922906	1.643720512023	-0.234751170055
C	-1.764057672118	-2.597574559402	0.342013117282
C	-2.862709491342	-1.683003560451	0.248090096163
C	0.330041136830	2.094616171339	-0.124583639882
C	1.486996846815	1.250229719573	0.012674569573
C	-0.154475122700	-0.639552137128	0.447107301012
C	-1.206933437226	0.151455751434	-0.391080351242
C	-0.324673474666	-0.358664960519	1.975333800340
C	-1.067251665284	-0.173118478359	-1.912558404704
C	-4.174062108591	-2.198973315473	0.352251045939
C	-2.056386493892	-3.968064224960	0.561029329646
C	-4.425553698270	-3.542823674930	0.556459430503
C	-3.350270700197	-4.437633997414	0.666078257026
H	3.217639599435	-0.784664524575	-0.467311990657
H	-1.845316365089	3.599682550934	-0.322834800664
H	0.473099236645	-4.031219902862	-0.121486105172
H	-4.612412124527	0.392773435651	0.270817433836
H	0.471073853553	3.169030432643	-0.067716330741
H	-1.315660971687	-0.652768384365	2.327360979166
H	-1.264417077099	-1.227335829394	-2.117714281813
H	-0.176704527735	0.699174715508	2.199127883131
H	-0.061781511554	0.068054936184	-2.264686373857
H	0.419176223668	-0.935691067339	2.529436954876
H	-1.783834601517	0.427320682530	-2.477059427235
H	-5.018358266542	-1.528689385686	0.253640647458
H	-1.240477210255	-4.669879673658	0.678073580070
H	-5.446828373223	-3.899204216728	0.629484131882
H	-3.530979185652	-5.492562581389	0.840546658866
C	2.765716015728	1.867313733852	0.143042703942
C	3.033365186175	3.196542260741	-0.342764258623
C	3.889253202541	1.269954399893	0.815686627408
C	4.247225785520	3.793211816724	-0.196652335871
H	2.276784604270	3.746465691658	-0.884470155697
C	5.078289846430	1.916203019282	0.957102148887
H	3.791952255247	0.302350166128	1.285035087343
H	4.455839777582	4.777590140200	-0.592771863956
H	5.909733897153	1.479955470123	1.493993501772
N	5.288061426050	3.176196459952	0.452154152386
C	6.613460318567	3.794564094191	0.506377183991
H	7.120596340552	3.483228026422	1.418840618497
H	7.217307016955	3.502085093351	-0.357025818296
H	6.506206612903	4.878201734775	0.517277882158
C	3.151721921219	-3.479026044629	-0.542195168515
C	3.675656452549	-3.193232569365	-1.971070567432
C	4.304916153543	-3.268249485143	0.468662361944
C	2.734208627477	-4.960330335026	-0.476909864837
H	2.886572434008	-3.350546902727	-2.711926572416
H	4.033956132027	-2.166249734216	-2.073599050867
H	4.507364230538	-3.862967876595	-2.212679034795
H	3.970031285433	-3.475814089296	1.489057190102
H	5.137468693704	-3.941415805156	0.240598385862
H	4.687955083395	-2.245551795458	0.441365626376
H	3.601220287282	-5.593213458529	-0.684983527159
H	2.354443082608	-5.231230776599	0.512226255061
H	1.966278038801	-5.202427569570	-1.216484685142
C	-4.575267979956	3.051171663803	-0.174140449711
C	-5.540406907537	2.628316524148	-1.307927322714
C	-5.319314013637	2.974386360832	1.181227963257
C	-4.172787836026	4.517396197377	-0.413832842069
H	-5.047376514270	2.695540415585	-2.281967309485
H	-5.894946551944	1.602769137092	-1.180645031917
H	-6.416229790791	3.284429108437	-1.324128795727
H	-4.663953833931	3.282096048765	2.000964249442
H	-6.188542554756	3.639325650497	1.172709582000
H	-5.677556443262	1.965200497999	1.397027186486
H	-5.068946193075	5.143177765986	-0.441897294160
H	-3.528832447278	4.898061384154	0.383492558490
H	-3.648875083436	4.642320648007	-1.365403331605

Unrestricted B3LYP/6-311G(d,p) **BCPDPy•** optimized Cartesian coordinates in acetonitrile.

C	-0.870040245493	-1.941122931374	0.996176338389
C	0.475566767417	3.248329051568	-0.175211306159
C	0.136905189073	-2.892196971869	1.201988680160
C	1.736475494452	3.565729087505	-0.674207221874
C	1.391100409041	-2.581113465109	0.672513975737
C	2.718592489445	2.565062242790	-0.569684485765
C	1.594060766130	-1.486264593376	-0.184450875687
C	2.523814716635	1.410755504387	0.201060393430
C	-0.707556226665	-0.842821966558	0.142836012437
C	0.218393648986	2.052620319813	0.536389637050
C	3.016066618298	-1.042613454582	-0.387055209744
C	3.471013676268	0.264829846097	-0.013480380183
C	-1.169252567837	1.595802493319	0.582945667776
C	-1.662134956489	0.316040744143	0.279209817723
C	0.480616690137	-0.737144574065	-0.617657146068
C	1.335188633181	1.277374110975	0.951420950183
C	0.541633867704	0.107959867066	-1.865334205286
C	1.289409720457	0.427569118355	2.194951991533
C	4.851546329908	0.510725341522	-0.018149773681
C	3.969560892164	-1.987403777558	-0.790997792089
C	5.778983204732	-0.453888112447	-0.403546832829
C	5.333664273246	-1.706892580377	-0.810759714757
H	-1.800285161185	-2.017984511491	1.547716528393
H	-0.372094305282	3.887380340270	-0.393647429865
H	2.258027065468	-3.160680054450	0.962080949898
H	3.641864532515	2.657742476582	-1.129396646652
H	-1.897444168502	2.401463120061	0.561238121586
H	1.518821411017	0.561045537970	-2.025452972555
H	2.126990720141	-0.267093346353	2.246010877847
H	-0.213455874539	0.891623916622	-1.868334660460
H	0.362692545402	-0.129559169661	2.313638790674
H	0.342761145408	-0.543519381076	-2.725390906709
H	1.375288932826	1.097850570425	3.060082248035
H	5.202269565472	1.489246150095	0.290311937115
H	3.625863732791	-2.969821116442	-1.095424211312
H	6.838113164355	-0.222355655238	-0.389749543667
H	6.038010341095	-2.465445401209	-1.133135932859
C	-3.066364907632	0.079725378015	0.145931016196
C	-4.078370603757	1.009471360298	0.589151010498
C	-3.598142247594	-1.091986487191	-0.493026794849
C	-5.402572288626	0.778886019320	0.393752112813
H	-3.806183284214	1.909187510596	1.123356228003
C	-4.933245048886	-1.278958700197	-0.675429232984
H	-2.927532532257	-1.851508767884	-0.871596144490
H	-6.163912918577	1.464281264025	0.740989702082
H	-5.335101765426	-2.152569882565	-1.170051224636
N	-5.863184249899	-0.345762693268	-0.254775857305
C	-7.292971669891	-0.625571596218	-0.355298181619
H	-7.498165377998	-1.146635223458	-1.290711296586
H	-7.636596503834	-1.244767642789	0.479400943741
H	-7.846190864779	0.312732036130	-0.351607872813
C	-0.127043776774	-4.132390948385	2.073971092250
C	-0.509965311202	-3.700493890449	3.508780757545
C	-1.293135568779	-4.941502347974	1.459596710313
C	1.102713126618	-5.054805134662	2.161690578504
H	0.297870998099	-3.126292516364	3.971125020821
H	-1.411710003874	-3.083896783957	3.520022564504
H	-0.700061076665	-4.580639225633	4.130984110190
H	-1.044324126032	-5.276888292036	0.448587792620
H	-1.504271168713	-5.825997956778	2.068652526737
H	-2.208431006331	-4.347646679372	1.401340947053
H	0.852988927173	-5.942591845532	2.749039317990
H	1.428485580971	-5.389289133930	1.172877464100
H	1.947627901894	-4.563662051280	2.651861241128
C	2.042843723080	4.877854498373	-1.418525068167
C	3.232786010384	5.584954376821	-0.728349616045
C	2.414918503600	4.580206708091	-2.889843356485
C	0.844337819392	5.844398429432	-1.413162048013
H	2.993534561806	5.822897617958	0.312197566145
H	4.129004552280	4.960104673477	-0.732590323474
H	3.471860496076	6.520179350027	-1.244456073649
H	1.589318219065	4.082638016616	-3.406613644560
H	2.636024796305	5.511441603571	-3.420962295361
H	3.295420763110	3.937825723475	-2.964437115540
H	1.123829097254	6.777172845700	-1.910663815664
H	-0.015534757831	5.431394925927	-1.947390161268
H	0.528283444690	6.090038435745	-0.395537689496

Unrestricted B3LYP/6-311G(d,p) transition state optimized Cartesian coordinates in acetonitrile for **BDHPPy•/BCPDPy•** thermal conversion.

C	-0.907728155419	-1.948954980418	0.965028079962
C	0.545247543957	3.356837096986	0.016142829505
C	0.079860636910	-2.937993790602	1.163980868357
C	1.832032991442	3.676146934388	-0.341482319685
C	1.371435210305	-2.645000163491	0.695201602584
C	2.786270553733	2.597494185149	-0.389314955095
C	1.654223904309	-1.504807667882	-0.050516117726
C	2.532407504760	1.343196917577	0.103512336325
C	-0.697671694609	-0.800887457466	0.217234147839
C	0.160044199628	2.033957641386	0.407367954886
C	3.069868862704	-1.117272006673	-0.284362386926
C	3.503752901495	0.230716608262	-0.090897316302
C	-1.181399189961	1.651511977791	0.341475382415
C	-1.685460269407	0.319129562103	0.225108591648
C	0.599855694049	-0.580765499004	-0.397584230351
C	1.228462904778	1.064353945076	0.710835813841
C	0.619345878003	0.006338623586	-1.804001122710
C	1.253333372933	0.526263483381	2.132129588065
C	4.881085828339	0.494885771892	-0.150459110416
C	4.025753174457	-2.092623296126	-0.604310444636
C	5.814160721724	-0.494339535754	-0.449092720256
C	5.382949373118	-1.795672691884	-0.693519618864
H	-1.872639133938	-2.070632773160	1.444747920443
H	-0.245570181391	4.096061509819	-0.052248665413
H	2.195305697856	-3.295325903435	0.959840125695
H	3.741775000788	2.780844840909	-0.867273842572
H	-1.887838842428	2.461068370003	0.183418964398
H	0.239438805314	-0.746228955741	-2.504035849192
H	1.983347003420	-0.275013507334	2.256214288285
H	1.628808073598	0.272167531106	-2.118330712100
H	0.273421577462	0.161356446007	2.443073524919
H	-0.013295800447	0.889685768393	-1.885717982796
H	1.536394348443	1.340330701472	2.810341547888
H	5.230422651551	1.499660231253	0.056367296922
H	3.691050140237	-3.106641229075	-0.792494227793
H	6.869223638966	-0.247247354326	-0.487425457477
H	6.093966120534	-2.575489484064	-0.942150165741
C	-3.067772992176	0.092503703498	0.073574161758
C	-4.074823373453	1.086411557265	0.370462529459
C	-3.620480140779	-1.142670498995	-0.427796004568
C	-5.399770808479	0.851717947115	0.183509049569
H	-3.798205540121	2.041586818911	0.793497651793
C	-4.956220339459	-1.320709564384	-0.604456397805
H	-2.964842952401	-1.954488616904	-0.707434116432
H	-6.152236719314	1.591702017235	0.420995672855
H	-5.365448542624	-2.238741229235	-1.004045882023
N	-5.871307224406	-0.344370295901	-0.295413246857
C	-7.296788846681	-0.536915160093	-0.567273632251
H	-7.546171286377	-0.231753267326	-1.587209528220
H	-7.551729136424	-1.587703469400	-0.435956921250
H	-7.881662845528	0.054646494304	0.135805581765
C	-0.258699513644	-4.214163216142	1.950006209144
C	-0.676834448750	-3.848329957150	3.394623294245
C	-1.430801158540	-4.948001050565	1.255371704855
C	0.932581766726	-5.186920168551	2.028063573328
H	0.136282491314	-3.335695141066	3.916349097596
H	-1.552152709596	-3.194731663317	3.409705700593
H	-0.925495961021	-4.752978475338	3.958640197501
H	-1.159127511055	-5.236729749625	0.235929827191
H	-1.690808145001	-5.856317793212	1.808054929283
H	-2.325007573081	-4.322636336555	1.201261910555
H	0.630101449962	-6.093836520271	2.558647273930
H	1.279566460203	-5.481953746567	1.033905825506
H	1.777658315393	-4.755102136690	2.570887778985
C	2.271774870355	5.081148238653	-0.780371192375
C	3.457868543652	5.545750804788	0.098419455381
C	2.719588519752	5.059080482292	-2.261730396851
C	1.140816625225	6.115798896322	-0.639277028849
H	3.164428310377	5.595132121996	1.151039754966
H	4.310972430034	4.867533232027	0.019127712764
H	3.793008768794	6.541670100521	-0.208454816846
H	1.896725954702	4.745934436610	-2.910677215136
H	3.040537159615	6.057137972365	-2.576803605511
H	3.556148323344	4.375547211813	-2.425317992399
H	1.509236905937	7.104339041955	-0.927190374865
H	0.290365727587	5.879780857264	-1.284621885424
H	0.779837598725	6.180589292249	0.390935166715

10. References

(1) Roldan, D.; Cobo, S.; Lafolet, F.; Vilà, N.; Bochot, C.; Bucher, C.; Saint-Aman, E.; Boggio-Pasqua, M.; Garavelli, M.; Royal, G. A Multi-Addressable Switch Based on the Dimethyldihydropyrene Photochrome with Remarkable Proton-Triggered Photo-Opening Efficiency. *Chem. - Eur. J.* **2015**, *21* (1), 455–467.

(2) Mitchell, R. H.; Ward, T. R. The Synthesis of Benz-, Naphth-, and Anth-Annelated Dihydropyrenes as Aids to Measuring Aromaticity by NMR. *Tetrahedron* **2001**, *57* (17), 3689–3695.

(3) Sheepwash, M. A. L.; Mitchell, R. H.; Bohne, C. Mechanistic Insights into the Photochromism of *Trans*-10b,10c-Dimethyl-10b,10c-Dihydropyrene Derivatives. *J. Am. Chem. Soc.* **2002**, *124* (17), 4693–4700.
